# Lower blood pH as a strong prognostic factor for fatal outcomes in critically ill COVID-19 patients at an intensive care unit: A multivariable analysis

**DOI:** 10.1371/journal.pone.0258018

**Published:** 2021-09-29

**Authors:** Martin Kieninger, Annemarie Sinning, Timea Vadász, Michael Gruber, Wolfram Gronwald, Florian Zeman, Dirk Lunz, Thomas Dienemann, Stephan Schmid, Bernhard Graf, Matthias Lubnow, Thomas Müller, Thomas Holzmann, Bernd Salzberger, Bärbel Kieninger

**Affiliations:** 1 Department of Anesthesiology, University Medical Center Regensburg, Regensburg, Germany; 2 Department of Infection Prevention and Infectious Diseases, University Medical Center Regensburg, Regensburg, Germany; 3 Institute of Functional Genomics, University of Regensburg, Regensburg, Germany; 4 Center for Clinical Studies, University Medical Center Regensburg, Regensburg, Germany; 5 Department of Surgery, University Medical Center Regensburg, Regensburg, Germany; 6 Department of Internal Medicine I, University Medical Center Regensburg, Regensburg, Germany; 7 Department of Internal Medicine II, University Medical Center Regensburg, Regensburg, Germany; Heidelberg University Hospital, GERMANY

## Abstract

**Background:**

Data of critically ill COVID-19 patients are being evaluated worldwide, not only to understand the various aspects of the disease and to refine treatment strategies but also to improve clinical decision-making. For clinical decision-making in particular, prognostic factors of a lethal course of the disease would be highly relevant.

**Methods:**

In this retrospective cohort study, we analyzed the first 59 adult critically ill Covid-19 patients treated in one of the intensive care units of the University Medical Center Regensburg, Germany. Using uni- and multivariable regression models, we extracted a set of parameters that allowed for prognosing in-hospital mortality.

**Results:**

Within the cohort, 19 patients died (mortality 32.2%). Blood pH value, mean arterial pressure, base excess, troponin, and procalcitonin were identified as highly significant prognostic factors of in-hospital mortality. However, no significant differences were found for other parameters expected to be relevant prognostic factors, like low arterial partial pressure of oxygen or high lactate levels. In the multivariable logistic regression analysis, the pH value and the mean arterial pressure turned out to be the most influential prognostic factors for a lethal course.

## Introduction

Severe acute respiratory syndrome coronavirus type 2 (SARS-CoV-2) is a new beta coronavirus that was identified as the cause of coronavirus disease 2019 (COVID-19) in early 2020. In the spring and summer of 2020, about 10% of those infected with SARS-CoV-2 in Germany required hospital treatment, of whom 14% had to be treated at an intensive care unit (ICU). The mortality rate of ICU patients with COVID-19 was 47% [1, 2]. Although originally assumed to be a ‘lung disease,’ COVID-19 has a broad organotropism and causes neurological, nephrological, cardiological, and hematological problems. Typical of severe courses are dramatic inflammatory reactions of the monocyte-macrophage system similar to those observed in severe acute respiratory syndrome (SARS) and Middle East respiratory syndrome (MERS) [3, 4].

Three recently published studies that included more than 15000 patients with COVID-19 from South Korea, France, Belgium, Switzerland, Denmark, and Great Britain have shown that advanced age as well as pre-existing comorbidities were significantly more common in non-survivors than in survivors [5–7]. The complication rate depended on both age and co-morbidities and characterized the patients who required intensive care treatment [8]. For patients with COVID-19 who require ICU treatment, various parameters have already been suggested as prognostic factors of outcome. However, no consistent overall picture has yet been drawn. Choron et al. found the course of fever to be an important prognostic factor [9]. Pan et al. included lymphocyte percentage, prothrombin time, lactate dehydrogenase, total bilirubin, eosinophil percentage, creatinine, neutrophil percentage, and the albumin level in their model [10]. Thomson et al. found age, obesity, the lowest Horowitz index (P/F ratio) on the first day of admission, and paCO_2_ to be independently associated with ICU death [11]. Mesas et al. identified Interleucin 6 (IL-6) together with pre-existing diseases as prognostic factors of a lethal course in patients under the age of 60 and elevated bilirubin and liver enzymes together with decreased albumin in patients older than 60 years [12]. Mennuni et al. found that the dosage of low molecular weight heparin hardly affected outcome [13].

## Material and methods

### Aim of the study

Our study focused on COVID-19 patients who already required high-care intensive medicine. The aim was to identify prognostic factors for fatal outcomes in this population. We evaluated 103 different parameters assumed to be crucial for ICU treatment and performed further analysis using logistic regression models. The study included all patients who had to be treated at the ICUs of the University Medical Center Regensburg, Germany, during the first wave of the COVID-19 pandemic.

### Ethics approval and consent to participate

The study was approved by and conducted according to the guidelines of the Ethics Committee of the University of Regensburg (approval number 20-1790-104, ‘S1 Appendix’). In accordance with European law, consent to participate was not required due to retrospective analysis of anonymized patient data. All data were anonymized before being accessed.

### Patients and settings

This retrospective study included 59 adult critically ill Covid-19 patients (40 men, 19 women) who had been admitted to one of the ICUs at the University Medical Center Regensburg between 14 March 2020 and 2 June 2020. All patients were treated according to our in-house standard described in ‘S1 Table.’

**Fig 1:**
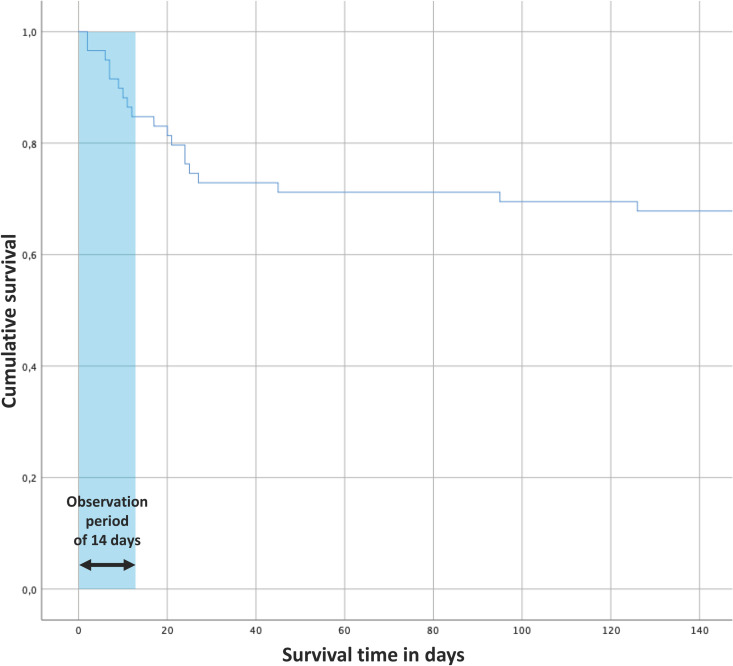
Kaplan-Meier estimator for all patients. Out of 59 patients included in the study, 19 did not survive. Nine patients died within the observation period of the first 14 days of intensive care treatment. One patient spent 126 days in intensive care before dying. Forty patients were able to leave the intensive care unit alive.

The cohort consisted of patients who were either directly admitted to the University Medical Center Regensburg or who were transferred from a non-tertiary hospital in the surrounding area for higher-care therapy. Three patients were transferred from the Bergamo area in Italy. There were no standardized criteria for admission of the patients to the ICU at the University Medical Center Regensburg from which the present study’s data has been drawn. Forty patients were discharged from the ICU alive; 19 had died (mortality 32.2%). Fig 1 shows the Kaplan-Meier estimator for all patients.

### Data collection

In each case, we examined the first two weeks of ICU treatment or the time until the patient died or was discharged from the ICU. For the investigated period, we collected parameters from the following categories: (1) baseline and demographic data, (2) pre-medication, (3) pre-existing comorbidities, (4) vital signs, (5) dosage of catecholamines, (6) dosage of analgosedation, (7) anticoagulation and antithrombotic medication, (8) laboratory blood diagnostics and microbiological diagnostics, (9) extracorporeal membrane oxygenation (ECMO), prone positioning, and ICU scores, (10) airway, respirator therapy, and pulmonary gas exchange and (11) complications during ICU treatment. A complete list of all the parameters we examined is provided in ‘S2 Table.’ Some parameters were recorded daily. It should be noted that not all patients survived the observation period and that some patients were discharged from the ICU before the end of the observation period; therefore, not all data sets are complete. Complete data sets consisted of 883 values per patient. Data were extracted from the in-house patient data management systems of the ICU (MetaVisionSuite®, version V6.9.0.23, iMDsoft®, Tel Aviv, Israel; SAP® Enterprise resource planning, version 6.0 EHP7 SP21, SAP SE, Walldorf, German; SWISSLAB® Laborinformationssysteme, version 2.18.3.00, NEXUS SWISSLAB GmbH, Berlin, Germany). The patients were grouped according to their outcome (‘died:’ death in intensive care, ‘survived:’ transferred to a rehabilitation or normal care unit); the outcome was determined on the day of discharge from the ICU.

### Statistical analysis

Statistical analysis was conducted using IBM SPSS Statistics^TM^ 26 (IBM, Armonk, USA). Statistical tests were two-sided, and the level of significance was set to p<0.050. Categorical parameters are presented as absolute and relative frequencies and illustrated by bar charts. Survivors and non-survivors were compared by using the Chi-square test of independence. Continuous data are shown as median and minimum/maximum, and differences between survivors and non-survivors were assessed with the Mann-Whitney-U Test. Continuous parameters with values for each day of examination were analyzed in two steps. First, we investigated the course of the parameters within the first 14 days of ICU treatment. We calculated the median and interquartile range for survivors and non-survivors on a daily basis and compared both groups using the two-sided Mann-Whitney-U Test. The results are visualized by box plots. Second, we investigated the parameters with apparently relevant differences between the two groups on several days during the observation period. For this purpose, we calculated mean values and, if reasonable from a clinical point of view, minimum and maximum values for each patient over the course of time, leading to a total of 92 parameters, which were then compared between survivors and non-survivors using the Mann-Whitney-U Test. To account and correct for multiple comparisons, Bonferroni correction was applied and a new significance level p* = 5.43*10^−4^ was determined. In a next step, all parameters with p<p* in the univariable analysis were extracted for further examination.

In the univariable regression models a cut-off value for the probability of 0.5 for non-survival was calculated for each parameter. Subsequently, patients with below-cut-off values (or above the cut-off for troponin Tmean) were assigned to a subgroup, and their relative mortalities were calculated.

Finally, a multivariable logistic regression model was calculated. Multicollinearity between these parameters was assessed by using Pearson’s correlation coefficient. Odds ratios and 95%-confidence intervals-p-values and c-indices are reported for all logistic regression models. Receiver operating characteristics (ROC) curves were used for evaluation of the model. Finally, a leave-one-out cross validation (LOOCV) was carried out to determine the model accuracy.

## Results

### Statistical analysis

#### Baseline and demographic data (‘S1 Fig’)

Patient age differed significantly (p = 0.010) between survivors (patients who were discharged from the ICU alive) (median age 56 years, minimum 21 years, maximum 73 years) and non-survivors (patients who died at the ICU) (median age 66 years, minimum 44 years, maximum 76 years). Female and male patients were distributed equally in the two groups (13 women and 27 men survived, 6 women and 13 men died, p = 1.000). The two groups differed neither in body mass index (survivors: median 27.8 kg/m^2^, minimum 20.8 kg/m^2^, maximum 40.4 kg/m^2^; non-survivors: median 29.3 kg/m^2^, minimum 19.2 kg/m^2^, maximum 46.8 kg/m^2^; p = 0.608) nor in the distribution of blood group characteristics (AB0 system: p = 0.764, Rhesus system: p = 0.582). Forty-one patients had already been treated at an ICU in a non-tertiary hospital. Duration of ICU treatment prior to the transfer to our hospital did not differ between the two groups (survivors: median 2.5 days, maximum 21 days; non-survivors: median 4 days, maximum 23 days; p = 0.803).

#### Pre-medication and pre-existing comorbidities (‘S2 Fig’)

Pre-medication and pre-existing comorbidities were recorded according to the classification provided in S2 Table. The frequencies for all drug classes of pre-medication and all types of pre-existing comorbidities were compared between the two groups using pairwise comparison. No statistically significant differences were found. The percentages and p-values for each pairwise comparison are provided in S2 Fig. The rate of patients without any pre-medication did not differ between the two groups (survivors: 17, non-survivors: 6; p = 0.570).

#### Vital signs (‘S3 Fig’)

**Fig 2:**
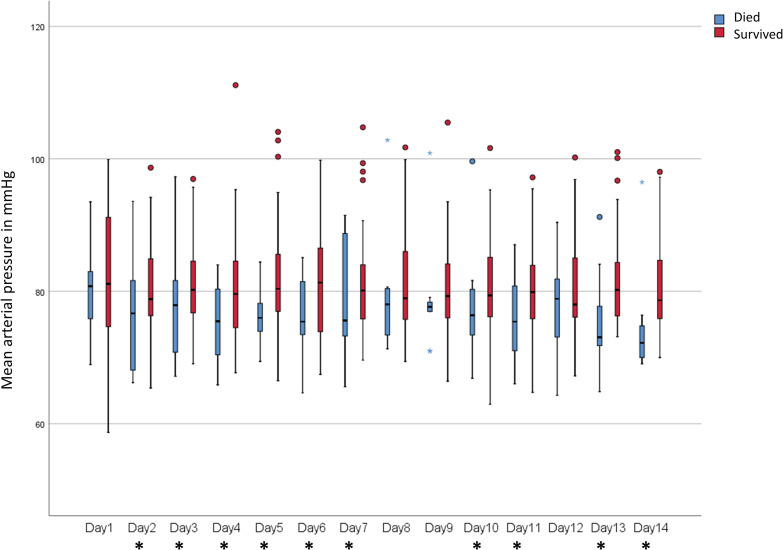
Daily mean arterial pressure (MAP) within the first 14 days of intensive care treatment: non-survivors had lower MAP values on most days than survivors. Significant differences are marked with an asterisk in the legend of the x-axis.

Body temperature was recorded daily as a categorical variable with the two possible values ‘fever’ (daily temperature peaks of ≥38°C) and ‘no fever’ (otherwise). The frequencies of patients with fever did not differ between the two groups over the entire observation period. Regarding the daily mean values for heartrate (HR) and oxygen saturation (SpO_2_), we only found a statistically significant difference in SpO_2_ on one day. In contrast, the daily mean values for mean arterial pressure (MAP) were significantly lower in non-survivors on most of the first 14 days of ICU treatment (Fig 2). The comparison of the overall mean values for the daily mean values for MAP for each patient showed a highly significant difference between the two groups, with lower MAP values for non-survivors (survivors: median 81.3 mmHg, range 72.2–99.2 mmHg; non-survivors: median 74.6 mmHg, range 62.3–87.9 mmHg; p<0.001).

#### Dosage of catecholamines and analgosedation, anticoagulation and antithrombotic medication (‘S4 Fig’)

A comparison of the two groups regarding the daily mean hourly dosage of noreprinephine yielded a p-value of <0.050 on 6 out of 14 days, with higher values in the group of non-survivors. The overall mean values and the maximum for the daily mean hourly dosage rates for each patient showed highly significant differences between survivors and non-survivors (mean values: p = 0.002, maximum values: p = 0.001). The mean daily dosage of sufentanil, midazolam, and ketamine did not differ significantly between the two groups; however, non-survivors had received higher hourly dosages of propofol on most days of the second week. The comparison of the personal mean values for the mean daily hourly dosages of sufentanil, propofol, midazolam, and ketamine did not yield any significant differences between survivors and non-survivors (sufentanil: p = 0.612, propofol: p = 0.168, midazolam: p = 0.831, and ketamine: p = 0.431). Higher than prophylactical dosages of unfractionated (UFH) or low molecular weight heparin (LMWH) were administered more often to survivors. A significant difference was noted on 8 days of the 14-day observation period. Thirty-seven out of 40 survivors (92.5%) received half-therapeutic or therapeutic doses of heparin on more than 50% of the days observed, in contrast to only 11 out of 19 patients in the group of non-survivors (57%, p = 0.003) (patients who were in the ICU for 14 days or more therefore received heparin on at least 7 days; if a patient died during the observed 14 days, the 50% or more days of receipt of heparin is in reference to the survival time in the ICU in days). Acetylsalicylic acid (ASA) was administered sporadically in both groups during the first 7 days of ICU treatment. However, there was a trend of more patients of the survivors group receiving ASA at the end of the second week of the observation period.

#### Laboratory blood diagnostics and microbiological diagnostics (‘S5 Fig’)

**Fig 3:**
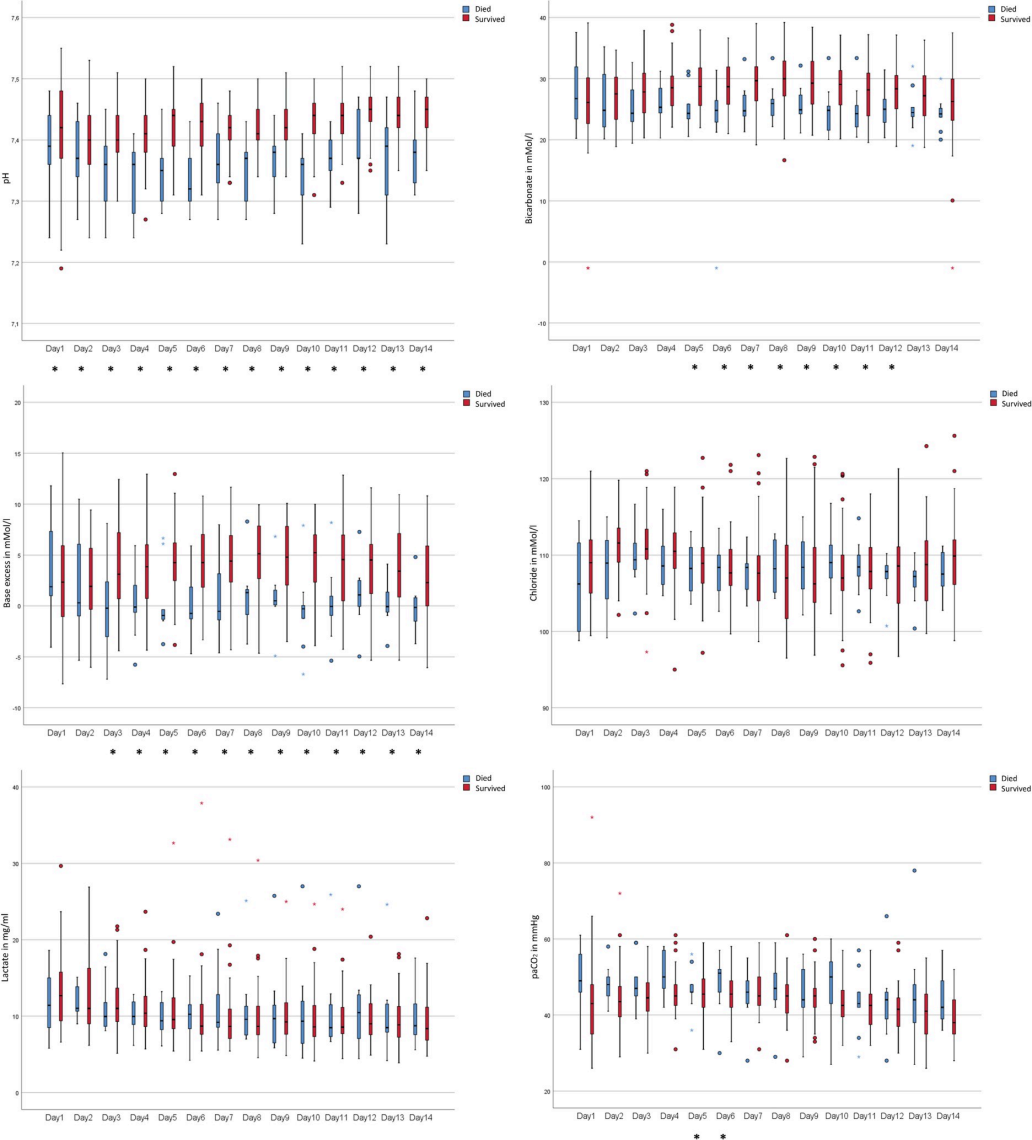
Daily mean values of blood pH, bicarbonate, base excess, chloride, lactate, and arterial partial pressure of carbon dioxide (paCO_2_) during the 14-day observation period for survivors and non-survivors: Values for pH, bicarbonate, and base excess were lower in non-survivors. Significant differences between the two groups are marked with an asterisk in the legend of the x-axis.

Mean daily blood pH values were significantly lower in non-survivors than in survivors on each of the 14 days (all p-values <0.050, most of them <0.010). The comparison of the overall mean daily pH values as well as the maximum and the minimum per patient yielded a significant difference of p<0.001 between the two groups. In line with these findings, base excess (BE) and bicarbonate values were also significantly lower in non-survivors for most of the days. Again, highly significant differences between the overall mean daily values and the maximum and minimum values per patient of BE were found (mean: p<0.001, maxima: p<0.001, minima: p = 0.001). The two groups also differed significantly in mean daily values and maximum values of bicarbonate (mean: p = 0.009, maximum: p = 0.003, minimum: p = 0.151). In contrast, blood lactate and chloride values did not differ between the two groups. For arterial partial pressure of carbon dioxide (paCO_2_), significant differences between survivors and non-survivors were only found at the very beginning (day 1 and day 2) and in the middle of the observation period (day 8). The results of the acid-base state are summarized in Fig 3. Mean daily values for troponin T also differed significantly between the two groups, with higher values for non-survivers on almost all days of the observation period. The comparision of the overall mean values and the maximum values per patient showed a highly significant difference between survivors and non-survivors, with p-values of ≤0.001. Noticeable differences between the two groups were also found for procalcitonin (PCT), again with higher values for non-survivors on almost all days of the observation period; the overall mean values for each patient differed highly significantly, with p<0.001. In addition, non-survivors showed a lower estimated glomerular filtration rate (eGFR) and a higher serum creatinine concentration on most days, and overall mean values for each patient differed significantly (eGFR: p = 0.009, creatinine: p = 0.014). All other parameters of laboratory blood diagnostics did not yield a consistent picture; significant differences between the two groups were found on less than 7 days of the observation period.

The proportion of patients with a proven high viral load >1*10^6^ copies at least once in the observation period hardly differed between the two groups (non-survivors: 10 out of 19, survivors: 17 out of 40, p = 0.579). Microbiological diagnostics were checked for further viral, bacterial, as well as fungal infections. Three temporal categories were formed: positive results at admission, positive results on days 1 to 7, and positive results on days 8 to 14. The number of patients per group was determined in each case. However, no significant differences could be found between the two groups.

#### ECMO, prone positioning, and ICU scores (‘S6 Fig’)

26% of non-survivors and 30% of survivors received ECMO therapy. For further examination, patients were divided into the following categories: cannulation start of ECMO prior to admission to our hospital, start of ECMO on day 1 to 7, start of ECMO on day 8 to 14, duration of ECMO treatment <5 days, and duration of ECMO treatment ≥5 days. The comparison of survivors and non-survivors showed that outcomes were neither influenced by the day of starting the therapy nor by the length of ECMO treatment. Therapy with prone positioning was recorded as metric data in hours per day. The duration of prone positioning did not differ between the two groups over the entire observation period.

The therapeutic intervention scoring system (TISS) score and the simplified acute physiology score (SAPS) were calculated daily for each patient. Although survivors and non-survivors differed in the TISS score on less than 7 days of the observation period, SAPS values were lower in survivors from day 2 to day 14. The comparison of the overall mean values for TISS and SAPS yielded a highly significant difference between the two groups (p<0.001).

#### Airway and respiratory therapy (‘S7 Fig’)

None of the patients underwent extubation during the first week of the observation period; however, 20% of survivors could be extubated in the second week. The two groups did not differ in the timing of tracheostomy. Interestingly, fraction of inspired oxygen (FiO_2_), positive endexpiratory pressure (PEEP), and tidal volume did not differ at all between survivors and non-survivors. Driving pressure was significantly lower in survivors only on two days of the first week (day 3 and day 4) but did not differ in the other days of the observation period. The P/F ratio never differed between the two groups.

#### Complications during ICU treatment (‘S8 Fig’)

The rate of serious complications within the first two weeks of ICU treatment was rather low for both survivors and non-survivors, except for acute kidney injury (AKI). Seventeen out of 19 non-survivors and 23 out of 40 survivors developed AKI (p = 0.017). Renal replacement therapy (RRT) was required in 63% of the non-survivors and in 30% of the survivors, corresponding to a p-value of 0.023. RRT was therefore significantly more often necessary in non-survivors. Twelve out of 19 non-survivors and only 7 out of 40 survivors had required RRT for at least 5 days (p = 0.001).

A notable finding was that intracerbral hemorrhage (ICH) was diagnosed in 4 non-survivors and in 1 survivor during the first two weeks of ICU treatment. Other bleeding complications occurred in 2 non-survivors and 3 survivors. No further statistical analyses could be performed due to the heterogeneity of the events (see ‘S3 Table’) and the low number of cases.

### Calculation of a model for prognosing fatal outcome

#### Selection of the parameters for the model

All parameters with explicit differences between survivors and non-survivors with p<5.43*10^−4^ (‘S4 Table’) were screened regarding their usefulness of inclusion in a model for prognosing fatal outcomes in critically ill COVID-19 ICU patients. SAPS and TISS values, which would both have met this criterion of usefulness, were excluded because both scores are already a combination of several parameters. PCT was also not considered because very high values of >100 ng/mL are only reported as ‘ = 100 ng/mL’ by the laboratory system, which factually excludes this metric parameter from use in a mathematical model.

#### Univariable logistic regression model

The following parameters were identified as highly significant prognostic factors of survival or death and used for the calculation of logistic regression models and the estimation of eligibility for multivariable model calculation: (1) mean MAP during the 14-day observation period for each patient, (2) mean, maximum, and minimum blood pH during the 14-day observation period for each patient, (3) mean, maximum, and minimum BE during the 14-day observation period for each patient, and (4) mean troponin T during the 14-day observation period for each patient. The best result could be achieved with the mean pH value over time (pHmean). The calculation provides the following formula of the probability of non-survival:

$$Probability\;of\;non\;survival=\frac{1}{1+e^{-x}}$$

withx=350.366−47.502*bloodpHmean

Characteristics of the calculation for univariable analysis can be found in ‘S5 Table’.

Table 1 shows the relative mortalities corresponding to the individual parameters identified as highly significant prognostic factors for non-surviving.

**Table 1:** Relative mortalities.

	Cut-off	Absolute mortality within patients with values below (MAP, pH, BE)/above (Troponin Tmean) cut-off	Absolute mortality within patients with values above (MAP, pH, BE)/below (Troponin Tmean) cut-off	Relative mortality
**MAPmean**	75 mmHg	9/15 patients (60.0%)	10/44 patients (22.7%)	2.64
**pHmean**	7.38	15/19 patients (78.9%)	4/40 patients (10.0%)	7.89
**pHmax**	7.44	8/10 patients (80.0%)	11/49 patients (22.4%)	3.57
**pHmin**	7.28	12/15 patients (80.0%)	7/44 patients (15.9%)	5.03
**BEmean**	-0.59 mmol/L	8/13 patients (61.5%)	11/46 patients (23.9%)	2.57
**BEmax**	2.68 mmol/L	10/15 patients (66.7%)	9/44 patients (20.5%)	3.25
**Troponin Tmean**	97.4 ng/L	6/8 patients (75.0%)	13/50 patients (26.0%)	2.88

MAPmean, mean MAP during the 14-day observation period for each patient; pHmean/pHmax/pHmin, mean, maximum and minimum blood pH during the 14-day observation period for each patient; BEmean/BEmax, mean, maximum BE during the 14-day observation period for each patient; Troponin Tmean, mean troponin T during the 14-day observation period for each patient.

#### Multivariable logistic regression model

We identified blood pH as a minimum value per patient (pHmin) and MAP as an averaged value per patient (MAPmean) as the best set of parameters for a logistic regression model. Following these determinations, the following formula for calculating the probability for fatal outcome resulted:

$$Probability\;of\;non\;survival=\frac{1}{1+e^{-x}}$$

withx=225.508−28.435*pHmin−0.238*MAPmean

**Fig 4:**
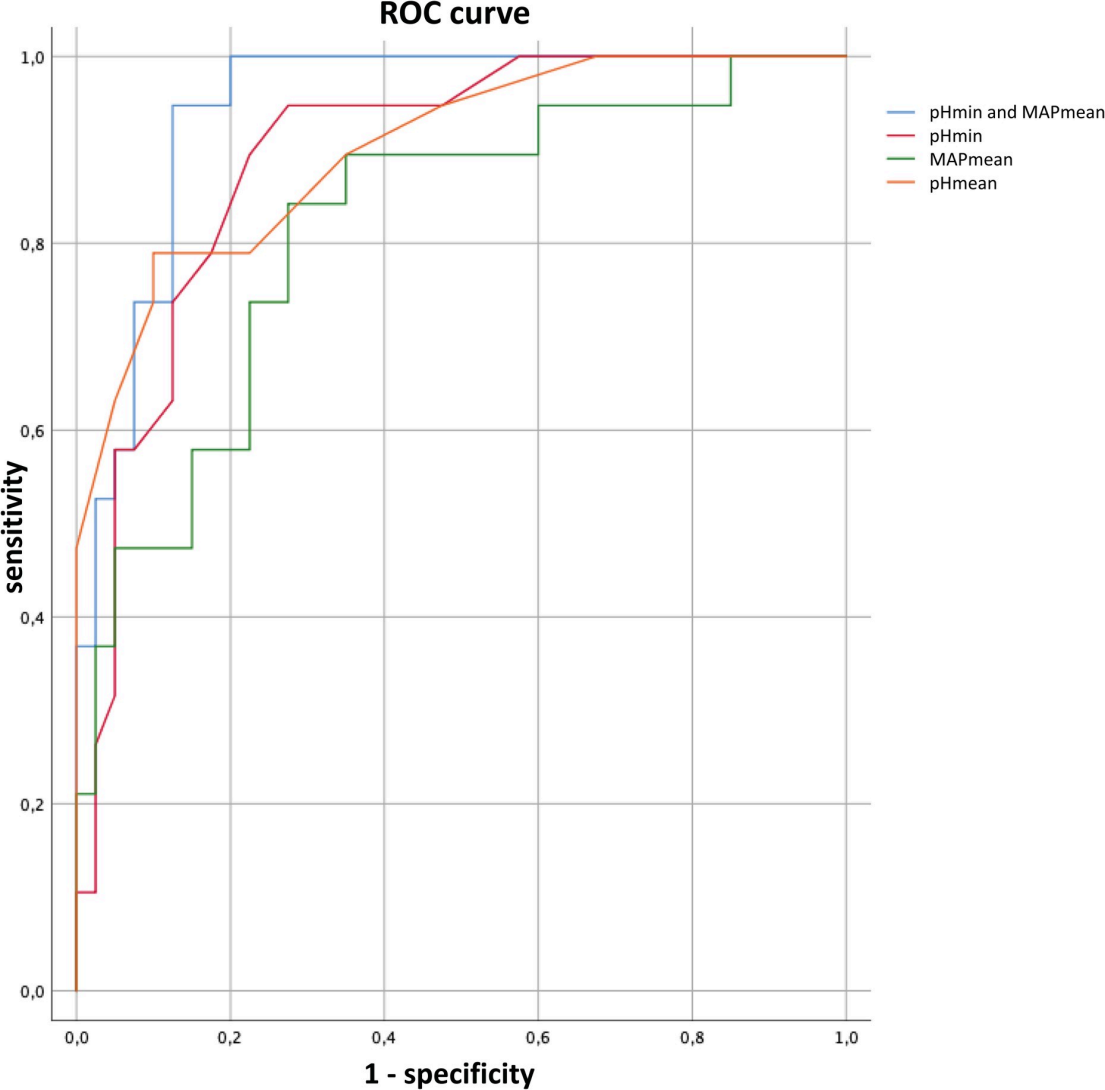
Comparison of receiver operating characteristics (ROC) curves. The calculated model with pHmin and MAPmean allows the best prognosis of outcome (area under the curve (AUC) = 0.945). Models with pHmin, MAPmean, and pHmean alone led to values of 0.893, 0.816, and 0.901. For better comparison, pHmin, MAPmean, and pHmean were multiplied by a factor of -1 when generating the ROC curve. MAPmean, mean MAP during the 14-day observation period for each patient; pHmean/pHmin, mean/minimum blood pH during the 14-day observation period for each patient.

A model accuracy of 84.7% was calculated by LOOCV. Fig 4 shows the comparison of ROC analysis for the multivariable model using MAPmean and pHmin and with the univariable models using MAPmean, pHmin and pHmean. Further parameters of the calculation for the multivariable analysis can be found in ‘S6 Table.’

## Discussion

The aim of the present study was to find prognosis factors for fatal outcomes in critically ill COVID-19 patients by investigating the first two weeks of treatment at a high-care ICU of a university medical center. For this purpose, we first screened the current literature for any parameters to be used as possible prognosis factors of good or bad outcomes in patients with COVID-19; furthermore, we added parameters that we considered crucial in everyday intensive care treatment. Consequently, a panel of 883 distinct parameters was generated for each patient if a complete dataset was available. The number of patients to be included was rather low, because we focused on those COVID-19 patients who had required the highest level of ICU therapy (41 out of 59 patients included in the present study had been transferred from an ICU of a non-tertiary hospital to an ICU at our university medical center). Despite this special selection, the cohort of patients included in the present study did not differ to the extent that could have been expected in terms of baseline and demographic data as well as pre-existing comorbidities [1, 5].

### Univariable analysis

In a first step, we checked all parameters suitable for prognosing patient outcome. Some results were in line with published data, and some results were unexpected. For example, in contrast to data published by Choron et al., fever was not a predictor of higher mortality in our study [9]. A possible reason for this apparent discrepancy may be different definitions of fever. We classified each day with a peak temperature of ≥ 38°C to be a ‘fever day,’ while Choron et al. had considered the absolute peak temperature levels for comparing survivors and non-survivors.

In the context of vital signs, MAP was a highly significant prognosis factor of patient outcome with lower values for non-survivors in our study. This finding is per se not surprising and in line with an obviously higher need for cardiocirculatory support of non-survivors who also required higher dosages of norepinephrine.

Serious courses of COVID-19 seem to be associated with a higher risk of venous thrombosis [13, 14]. In our cohort, higher prophylactical dosages of unfractionated or low molecular weight heparin were more often administered to survivors than to non-survivors. However, the heterogeneity of different dosages of UFH and LMWH in the study’s context with the overall small number of patients in our cohort does not allow any further conclusions on specific therapy recommendations regarding the intensity of anticoagulation.

Initial recommendations [15, 16] suggested that COVID-19 patients should not be treated with steroids. This guideline was applied when generating an initial in-house standard for the ICU treatment of COVID-19 patients during the first wave of the disease (‘S1 Table’). As the patients included in the present study had been treated according to this initial standard and had therefore hardly received any steroids, we did not consider treatment with steroids to be a reasonable parameter for prognosing outcomes in our cohort. Meanwhile, the efficacy of dexamethasone in the treatment of COVID-19 patients with respiratory support has been demonstrated [17].

Analysis of the laboratory diagnostics showed lower blood pH, BE, and bicarbonate values for non-survivors. Lactate, chloride, paO_2_ and paCO_2_, however, did not significantly differ in a relevant manner between the two groups. Choron et al. found acidosis to be a predictor of mortality in mechanically ventilated COVID-19 patients [9].

### Multivariable analysis and development of a model for prognostication of fatal outcomes

Calculation of the daily medians of metric parameters and the presentation as box plots for the 14-day observation period gives a visual impression of the question of whether there is a difference between survivors and non-survivors. Such daily comparisons, however, may be falsified, because patients were admitted to our ICU at different stages of their course of disease. ‘Day 1’ may be the day at which a patient first required ICU therapy but could also be the first day at our ICU after many days of ICU treatment in an external hospital. Therefore, mean or extreme values for each patient should be more conveniently considered in a multivariable analysis. In addition, the use of mean or extreme values does not imply that patients who died earlier than 14 days after ICU admission must be excluded from further calculation.

To account for the rather small size (59 patients) of our cohort for multivariable analysis and to avoid overfitting, the number of parameters selected for multivariable analysis was deliberately kept very low. We therefore only considered parameters that showed highly significant differences between the two groups for further examination. The derived formula correctly prognoses fatal outcome with a very high model accuracy of 84.7%, which underlines the practicality of the chosen approach. Fig 2 (for MAP) and Fig 3 (for pH) show the remarkable difference in both parameters between survivors and non-survivors over the entire observation period, indicating the high stability of the model. MAPmean and pHmin do not reflect any progressive divergence of the two parameters over time caused by the degeneration of patient status in non-survivors or the improvement of patient status in survivors.

Calculation of the linear regression model with only one parameter for pHmin, MAPmean and pHmean showed a highly prognostic value for pHmean that almost reached the level of the above model including MAPmean and pHmin (AUC 0.901 for pHmean alone vs. AUC 0.945 for MAPmean and pHmin). This finding was not expected and underlines the predictive strength of the pH value.

Nine patients from the non-survivors died during the 2-week observation period. One could assume that a relevant drop in blood pH value and MAP could have occurred within the last days before death due to multiple organ failure, thus causing explicit differences in pHmin and MAPmean between both groups and falsification of the model. However, exclusion of the last 3 days before death in all non-survivors for calculation does not result in relevant differences regarding the prognostication of fatal outcomes (‘S7 Table’).

### Limitations

The overall number of patients included in the present study is rather low, thus making multivariable analysis difficult. To avoid overfitting, we considered only parameters for calculation that had clearly yielded highly significant differences between the two groups over time and restricted the number of parameters included in the model.

We used a classical significance and regression analysis to examine parameters important in intensive care after an observation period of 2 weeks. The aim was to identify parameters that could help discriminate the survivor group from the non-survivor group in the further course. However, a multimodal prognostication that not only considers the results of statistical calculations but also performs a critical evaluation of the previous course of ICU treatment and the results of different kinds of diagnostics will be indispensable prior to withdrawal of life-sustaining treatment.

As the cohort of patients included in the present study was treated with the highest level of ICU therapy at a university hospital, our findings will possibly not be transferrable one to one to situations for patients treated at ICUs at smaller hospitals with more limited resources and logistical options.

We could only include 59 patients in our study, which may explain why some anticipated associations, such as the association between co-morbidities and ICU mortality, were not found.

Our study cohort consisted exclusively of patients from the first wave of the COVID-19 pandemic. Patients from the second and third wave may have different characteristics from those of the patients included in the present study. We have begun a follow-up study that additionally includes the patients of the second and third wave in Germany for reevaluation of our findings.

## Conclusions

A large number of parameters were screened as possible prognostic factors of fatal outcomes within a highly selective population of critically ill COVID-19 patients who required high-care ICU therapy in the first wave of the pandemic. MAP and blood pH were identified as prognostic factors because both parameters showed highly significant differences between survivors and non-survivors over the entire observation period. Both parameters can be easily derived from patient data management systems. Other parameters which were expected to be crucial for the further course of ICU treatment, like low paO_2_ or high lactate values, were not identified as relevant for the prognostication of a fatal outcome. A follow-up study including a higher number of patients and patients from the second and third pandemic waves will be necessary to reevaluate our findings.

## Supporting information

S1 Appendix Ethics approval document. (PDF)

S1 Table In-house standard for intensive care treatment of COVID-19 patients. (DOCX)

S2 Table List of all observed parameters. (DOCX)

S3 Table Overview of patients with intracerebral hemorrhage or other bleeding complications. (DOCX)

S4 Table Overview of all analyzed parameters that were recorded on a daily basis. (DOCX)

S5 Table Univariable logistic regression models on non-surviving. (DOCX)

S6 Table Multivariable logistic regression model on non-surviving. (DOCX)

S7 Table Calculation of the probability for a fatal outcome. (DOCX)

S1 Fig Visualization of baseline and demographic data. (DOCX)

S2 Fig Visualization of pre-medication and pre-existing comorbidities. (DOCX)

S3 Fig Visualization of vital signs. (DOCX)

S4 Fig Dosage of catecholamines, analgosedation, anticoagulation and antithrombotic medication. (DOCX)

S5 Fig Visualization of laboratory blood diagnostics and microbiological diagnostics. (DOCX)

S6 Fig Extracorporeal membrane oxygenation (ECMO), prone positioning, and ICU scores. (DOCX)

S7 Fig Airway and respiratory therapy. (DOCX)

S8 Fig Complications during ICU treatment. (DOCX)
