# Supplementary material for: Lower blood pH as a strong prognostic factor for fatal outcomes in critically ill COVID-19 patients at an intensive care unit: A multivariable analysis
Source: PLoS One. 2021 Sep 29;16(9):e0258018. doi: 10.1371/journal.pone.0258018 (PMC8480873; doi:10.1371/journal.pone.0258018)
Supplement: S1 Appendix — (PDF) [file pone.0258018.s001.pdf]

Universität Regensburg

Ethikkommission
an der Universität Regensburg

Ethikkommission · Universität Regensburg · 93040 Regensburg

Universitätsklinikum Regensburg
Klinik für Anästhesiologie
Priv.-Doz. Dr. Martin Kieninger
Franz-Josef-Strauß-Allee 11
93053 Regensburg

Prof. Edward K. Geissler, PhD, Vorsitzender

Ass. jur. Jan von Hassel, Geschäftsführer

Geschäftsstelle:
Telefon +49 941 943-5370
Telefax +49 941 943-5369
Postanschrift:
Universität Regensburg
ETHIKKOMMISSION
D-93040 Regensburg

ethikkommission@klinik.uni-regensburg.de
http://ethikkommission.uni-regensburg.de

14.04.2020

Unser Zeichen: 20-1790-104

Beratung nach § 15 Abs. 1 Berufsordnung für die Ärzte Bayerns

für das retrospektive Forschungsvorhaben mit dem Titel:

Course of Intensive Care Therapy in Patients with Severe Acute Respiratory Distress Syndrome following COVID-19

Antragsteller: Priv.-Doz. Dr. Martin Kieninger
Einrichtung: Universitätsklinikum Regensburg, Klinik für Anästhesiologie

Die Ethikkommission an der Universität Regensburg hat im vereinfachten Verfahren für das oben genannte Forschungsvorhaben eine Beratung gemäß § 15 der Berufsordnung für die Ärzte Bayerns durchgeführt und erhebt nach kursorischer Sach- und Rechtsprüfung und Abwägung des Nutzen-Risiko-Verhältnisses

keine berufsethischen oder rechtlichen Bedenken gegen die Durchführung dieses Forschungsvorhabens.

Der Entscheidung lag zugrunde das Antragsformular mit kurzer textlicher Projektbeschreibung vom 12.04.2020.

Hinweise:

1. Die ärztliche und juristische Verantwortung für die Durchführung dieses Forschungsvorhabens und die Richtigkeit der Angaben in diesem Beratungsverfahren verbleibt immer bei dem oder den jeweiligen, von der Kommission beratenen Forscher(n). Auf die Pflicht zur selbständigen Einhaltung einschlägiger Gesetze und Rechtsvorschriften wird hingewiesen.

2. Die Ethikkommission an der Universität Regensburg bestätigt, dass Sie auf der Grundlage der geltenden Gesetze, Vorschriften und der GCP/ICH-Richtlinien in der jeweils gültigen Fassung arbeitet. An der Studie Beteiligte haben nicht an der Entscheidung mitgewirkt.

3. Die Entscheidung erging im vereinfachten Verfahren, da nach Angaben des Antragsstellers nicht unter Einsatz von Arzneimitteln und/oder Medizinprodukten, nicht mit Strahlen, ohne studienbedingten Patienten- oder Probandenkontakt, rein retrospektiv und ohne Zugang externer Personen zu Quelldaten, ohne Datenerhebung außerhalb der eigenen Einrichtung des Forschers, ohne zu erwartende unmittelbare Konsequenzen aus den Forschungsergebnissen für konkrete Patienten, ohne klinische Interventionen an Patienten und/oder Probanden, ohne Datenerhebungen, welche über die Auswertung der Krankenakte nebst deren Anlagen hinausgehen, ohne externe Projektpartner, nicht im Rahmen eines Auftragsforschungsverhältnisses und ohne die Verwendung von Körpermaterialien geforscht werden soll.

4. Im vereinfachten Verfahren gelten folgende weitere Besonderheiten, auf die der Antragsteller hiermit hingewiesen wird:

   - Es können keine nachträglichen Änderungen bearbeitet werden. Gegebenenfalls wäre ein neuer Antrag zu stellen.

   - Es können keine nachträglichen Bestätigungen oder Kopien dieses Schreibens erstellt werden. Dieses Originalschreiben ist daher besonders sorgfältig zu verwahren.

5. Für dieses Verfahren werden keine Kosten erhoben.

Prof. Edward K. Geissler, PhD
Vorsitzender
