# Supplementary material for: Lower blood pH as a strong prognostic factor for fatal outcomes in critically ill COVID-19 patients at an intensive care unit: A multivariable analysis
Source: PLoS One. 2021 Sep 29;16(9):e0258018. doi: 10.1371/journal.pone.0258018 (PMC8480873; doi:10.1371/journal.pone.0258018)
Supplement: S1 Table — (DOCX) [file pone.0258018.s002.docx]

| **Isolation:**  In cases of suspected coronavirus disease 2019 (COVID-19), treatment in a single-bed box; in cases of confirmed COVID-19, cohort isolation in a 2-or 4-bed box |
| --- |
| **Virological or microbiological diagnostics:**   - Obtaining endotracheal aspirate and (1) RT-PCR testing for SARS-CoV-2 and (2) a routine bacteriological culture on admission to the ICU - Two pairs of blood cultures on admission to the ICU |
| **Laboratory testing (on admission to the ICU and frequent follow-ups):**   - Differential blood count - CRP, PCT, and IL-6 - Ferritin - LDH - Creatinine, urea, and phosphate - ASAT, ALAT, gamma-GT, alkaline phosphatase, bilirubin, cholinesterase, and albumin - Creatine kinase, troponin, and NTproBNP - INR, PTT, fibrinogen, and D-dimer |
| **Instrumental diagnostics:**   - Orienting echocardiography on admission to the ICU, repetition in the case of hemodynamic decline - Lung ultrasonography on demand - 12-channel ECG on admission to the ICU - Bedside chest X-ray on admission to the ICU and in the case of respiratory decline - CT of the chest and abdomen with a contrast agent on admission and, if necessary, in the case of respiratory decline (rationale: searching for a secondary infectious focus, aspect and extension of pulmonary lesions, and exclusion of pulmonary embolism) |
| **Antiviral agents:** Treatment not recommended because of the lack of evidence  **Antibacterial agents:** No prophylactic empiric antimicrobials, in the case of elevated PCT on admission and suspected bacterial superinfection, empirical initial therapy with piperacillin and tazobactam  **Antifungal agents:** Treatment only in the case of confirmed infection |
| **Systemic corticosteroids:** No routine use, but treatment is indicated in patients with suspected septic shock and insufficient response to vasopressor and fluid therapy |
| **Anticoagulation:**   - Consequent prophylactic SC administration of enoxaparin (40 mg 0-0-1) - In the case of suspected activation of blood clotting (elevated values of D-dimer), lack of clinical bleeding diathesis, and normal renal function, increased dosage of enoxaparin (40 mg 1-0-1) - In the case of AKI and during ECMO therapy, anticoagulation with continuous IV infusion of heparin with PTT target 50-60 s |
| **Sedation:**   - During the initial period, deep sedation with midazolam, ketamine, and sufentanil - If necessary, continuous administration of rocuronium |
| **Hemodynamics:**   - Conservative fluid strategy - Norepinephrine as a first-line vasoactive agent and, if necessary, vasopressin |
| **Ventilation:**   - PRVC mode for initial treatment - Low VT (6-8 mL/kg of predicted body weight) - Pplat < 30 cm H_2_O, driving pressure < 16 cm H_2_O - PEEP according to the ARDSNet protocol:   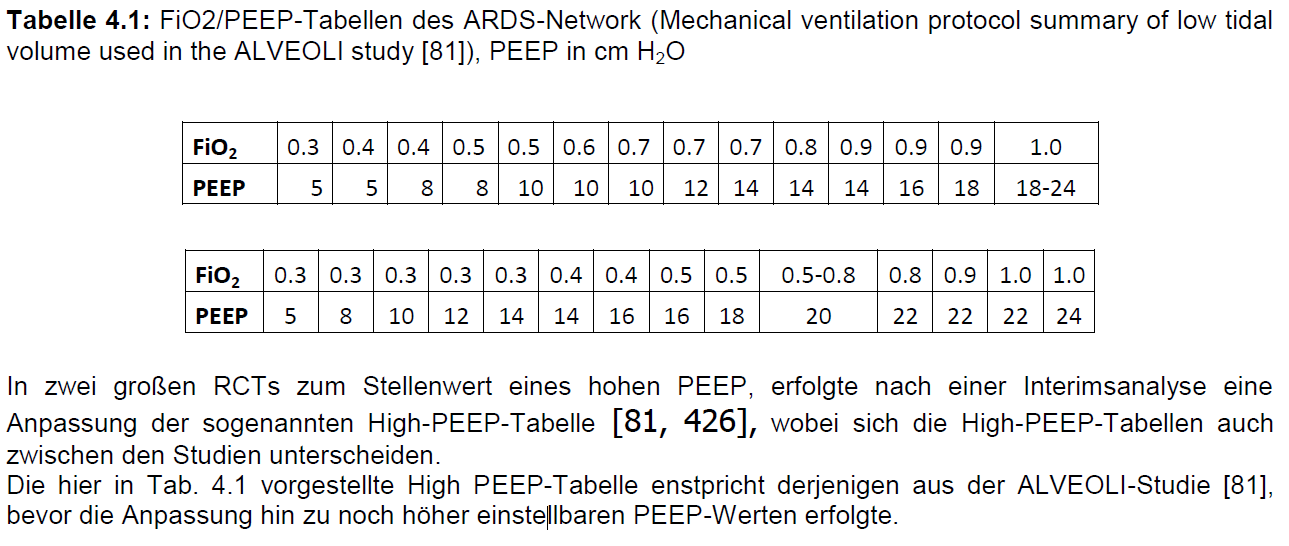   - PaO_2_ 65-80 mm Hg - PaCO_2_ 40-60 mm Hg - pH > 7.25 - In the case of PaO_2_/FiO_2_ < 150 mm Hg, consequent prone ventilation for at least 16 hours/day during the early course of the disease as long as improved pulmonary gas exchange is evident during proning - Inhaled ß2-mimetics only in the presence of obstructive ventilation disorder - Consider inhaled pulmonary vasodilators (iNO and iloprost) in the case of persisting hypoxemia despite optimized ventilation or in proven pulmonary hypertension |
| **ECMO:**   - Consider VV-ECMO in the case of persisting hypoxemia (PaO_2_/FiO_2_ 60-80 mm Hg at PEEP > 15 cm H_2_O) despite optimized ventilation, ineffective proning, or refractory severe hypercapnia with pH < 7.15 - Contraindications: age > 70 years or severe co-morbidities |
| Consider development of **secondary hemophagocytic lymphohistiocytosis** in the case of considerably high levels of ferritin and fever or acute worsening of the overall situation |

*SOP for the treatment of intubated patients with confirmed or suspected COVID-19 RT-PCR, reverse transcription polymerase chain reaction; ICU, intensive care unit; CRP, c reactive protein; PCT, procalcitonin; IL-6, interleukin-6; LDH, lactate dehydrogenase; ASAT, aspartate aminotransferase; ALAT, alanine aminotransferase; gamma-GT, gamma-glutamyl transferase; NTproBNP, N-terminal prohormone brain natriuretic peptide; INR, international normalized ratio; PTT, partial thromboplastin time; ECG, electrocardiogram; CT, computed tomography; SC, subcutaneous; AKI, acute kidney injury; IV, intravenous; PRVC, pressure regulated volume control; VT, tidal volume; Pplat, plateau pressure; PEEP, positive end expiratory pressure; PaO_2,_ arterial partial pressure of oxygen; PaCO_2_, arterial partial pressure of carbon dioxide; FiO_2_, fraction of inspired oxygen; iNO, inhaled nitrogen monoxide; VV-ECMO, venovenous extracorporeal membrane oxygenation*
