# Supplementary material for: Lower blood pH as a strong prognostic factor for fatal outcomes in critically ill COVID-19 patients at an intensive care unit: A multivariable analysis
Source: PLoS One. 2021 Sep 29;16(9):e0258018. doi: 10.1371/journal.pone.0258018 (PMC8480873; doi:10.1371/journal.pone.0258018)
Supplement: S2 Table — (DOCX) [file pone.0258018.s003.docx]

| **1 Base and demographic data** | | | |
| --- | --- | --- | --- |
| Age at admission to the ICU | metric | numeric | y |
| Sex | categorical | m = male  f = female |  |
| BMI | metric | numeric | kg/m² |
| Blood type | categorical | A  B  O  AB |  |
| Rh factor | categorical | p = positive  n = negative |  |
| Days on external ICU before ICU at UKR | categorical | integer | d |

| **2 Pre-medication** | | | |
| --- | --- | --- | --- |
| Antihypertensive drugs | categorical | 0 = no  1 = yes |  |
| Diuretics | categorical | 0 = no  1 = yes |  |
| Phosphodiesterase-5-inhibitors | categorical | 0 = no  1 = yes |  |
| Antidiabetics | categorical | 0 = no  1 = yes |  |
| L-Thyroxin | categorical | 0 = no  1 = yes |  |
| Thrombocytes-aggregation-inhibitors TAI | categorical | 0 = no  1 = yes |  |
| Anticoagulants | categorical | 0 = no  1 = yes |  |
| Opioids | categorical | 0 = no  1 = yes |  |
| Non-opioid analgesics | categorical | 0 = no  1 = yes |  |
| Steroids | categorical | 0 = no  1 = yes |  |
| Bronchospasmolytic drugs | categorical | 0 = no  1 = yes |  |
| Antidepressants | categorical | 0 = no  1 = yes |  |
| Dopamine agonists | categorical | 0 = no  1 = yes |  |
| Anticonvulsants | categorical | 0 = no  1 = yes |  |
| Lipidreducers | categorical | 0 = no  1 = yes |  |
| Proton-pump-inhibitors PPI | categorical | 0 = no  1 = yes |  |
| Immunosuppressive drugs | categorical | 0 = no  1 = yes |  |
| Supplements | categorical | 0 = no  1 = yes |  |
| Ursodeoxycholic acids | categorical | 0 = no  1 = yes |  |
| Alpha-5 reductase inhibitors | categorical | 0 = no  1 = yes |  |
| Uricostatic drugs | categorical | 0 = no  1 = yes |  |
| Other | categorical | 0 = no  1 = yes |  |

| **3 Pre-existing comorbidities** | | | |
| --- | --- | --- | --- |
| Cardiovascular | categorical | 0 = no  1 = yes |  |
| Pneumological | categorical | 0 = no  1 = yes |  |
| Autoimmune | categorical | 0 = no  1 = yes |  |
| Oncological | categorical | 0 = no  1 = yes |  |
| Neurological | categorical | 0 = no  1 = yes |  |
| Infectious | categorical | 0 = no  1 = yes |  |
| Nephrological | categorical | 0 = no  1 = yes |  |
| Degenerative | categorical | 0 = no  1 = yes |  |
| Obesity | categorical | 0 = no  1 = yes |  |
| Diabetes mellitus | categorical | 0 = no  1 = yes |  |
| Other metabolical diseases | categorical | 0 = no  1 = yes |  |
| Allergies | categorical | 0 = no  1 = yes |  |
| Noxious substances | categorical | 0 = no  1 = yes |  |
| Others | categorical | 0 = no  1 = yes |  |

| **4 Vital signs** | | | |
| --- | --- | --- | --- |
| Fever, daily peak value^A^ | categorical | 0 = daily peak value < 38°C  1 = daily peak value ≥ 38°C |  |
| Heartrate HR, daily average^A^ | metric | numeric | bpm |
| Mean arterial pressure MAP, daily average^A^ | metric | numeric | mmHg |
| Oxygen saturation SpO_2_, daily average^A^ | metric | numeric | % |

| **5 Dosage of catecholamines** | | | |
| --- | --- | --- | --- |
| Norepinephrine, daily average^A^ | metric | numeric | mg/h |

| **6 Dosage of analgosedation** | | | |
| --- | --- | --- | --- |
| Sufentanil, daily average^A^ | metric | numeric | μg/h |
| Propofol, daily average^A^ | metric | numeric | mg/h |
| Midazolam, daily average^A^ | metric | numeric | mg/h |
| Ketamin, daily average^A^ | metric | numeric | mg/h |

| **7 Anticoagulation and antithrombotic mediation** | | | |
| --- | --- | --- | --- |
| Unfractionated heparin or low molecular weight heparin in increased dose^AB^ | categorical | 0 = not received  1 = received |  |
| Acetylsalicylic acid^A^ | categorical | 0 = not received  1 = received |  |

| **8 Laboratory blood diagnostics and microbiological diagnostics** | | | |
| --- | --- | --- | --- |
| pH, daily average^A^ | metric | numeric |  |
| Bicarbonate ion HCO_3_^-^, daily average^A^ | metric | numeric | mMol/L |
| Base excess BE, daily average^A^ | metric | numeric | mMol/L |
| Cloride Cl^-^, daily average^A^ | metric | numeric | mMol/L |
| Partial pressure of oxygen paO_2_, daily average^A^ | metric | numeric | mmHg |
| Partial pressure of carbon dioxide paCO_2_, daily average^A^ | metric | numeric | mmHg |
| Hemoglobin Hb, daily average^A^ | metric | numeric | g/dL |
| Lactate, daily average^A^ | metric | numeric | mg/mL |
| Troponin^A^ | metric | numeric | ng/L |
| Glomerular filtration rate GFR^A^ | metric | numeric | mL/min/KOF |
| Creatinine^A^ | metric | numeric | mg/dL |
| Urea^A^ | metric | numeric | mg/dL |
| Aspartate transaminase AST^A^ | metric | numeric | U/L |
| Alanine transaminase ALT^A^ | metric | numeric | U/L |
| International Normalized Ratio INR^A^ | metric | numeric |  |
| Lactate dehydrogenase LDH^A^ | metric | numeric | U/L |
| C-reactive protein CRP^A^ | metric | numeric | mg/L |
| Procalcitonin PCT^A^ | metric | numeric | ng/mL |
| White blood cells WBC^A^ | metric | numeric | n/nL |
| Ferritin^A^ | metric | numeric | ng/mL |
| D-dimers^A^ | metric | numeric | mg/L |
| Platelets^A^ | metric | numeric | n/nL |
| Lymphocytes (absolute value) ^A^ | metric | numeric | x10ᶾ/μL |
| Interleukin 6^A^ | metric | numeric | pg/mL |
| Viral load SARS-CoV-2^D^ | categorical | h = high  l = low | copies/mL |
| Microbiological diagnostics (bacterial infection) ^A^ | categorical | 0 = positive result not received  1 = positive result received |  |
| Microbiological diagnostics (fungal infection) ^A^ | categorical | 0 = positive result not received  1 = positive result received |  |
| Microbiological diagnostics (viral infection)^A^ | categorical | 0 = positive result not received  1 = positive result received |  |

| **9 Treatment with extracorporeal membrane oxygenation, prone position, and ICU scores** | | | |
| --- | --- | --- | --- |
| Extracorporeal membrane oxygenation ECMO^A^ | categorical | 0 = no ECMO  1 = ECMO |  |
| Prone position^A^ | metric | numeric | h/d |
| Therapeutic intervention scoring system TISS^A^ | categorical | integer |  |
| Simplified Acute Physiology Score SAPS^A^ | categorical | integer |  |

| **10 Airway, respiratory therapy, and pulmonary gas exchange** | | | |
| --- | --- | --- | --- |
| Airway management^A^ | categorical | 0 = not made  1 = made |  |
| Attempted extubation^A^ | categorical | 0 = not made  1 = made |  |
| Tracheotomy^A^ | categorical | 0 = not made  1 = made |  |
| Fraction of inspired oxygen FiO_2_^A^ | metric | numeric | % |
| Positive endexpiratory pressure PEEP, daily average^A^ | metric | numeric | mmHg |
| Driving Pressure, daily average^A^ | metric | numeric | mmHg |
| Tidal volume VT, daily average^A^ | metric | numeric | mL |
| Oxygenation ratio (Horovitz), daily average^A^ | metric | numeric |  |

| **11 Complications during ICU treatment and renal replacement therapy (RRT)** | | | |
| --- | --- | --- | --- |
| Pulmonary embolism PE^A^ | categorical | 0 = PE not diagnosed  1 = PE diagnosed |  |
| Acute kidney injury AKI^A^ | categorical | 0 = AKI not diagnosed  1 = AKI diagnosed |  |
| Intracerebral hemorrhage ICH^A^ | categorical | 0 = ICH not diagnosed  1 = ICH diagnosed |  |
| Rhythm disturbances^A^ | categorical | 0 = Rhythm disturbances not diagnosed  1 = Rhythm disturbances diagnosed |  |
| Cardiac arrest^A^ | categorical | 0 = Cardiac arrest not diagnosed  1 = Cardiac arrest diagnosed |  |
| Cerebral ischemia^A^ | categorical | 0 = Cerebral ischemia not diagnosed  1 = Cerebral ischemia diagnosed |  |
| Bleeding complications^A^ | categorical | 0 = Bleeding complications not diagnosed  1 = Bleeding complications diagnosed |  |
| Pneumothorax^A^ | categorical | 0 = Pneumothorax not diagnosed  1 = Pneumothorax diagnosed |  |
| Pleural effusion^A^ | categorical | 0 = Pleural effusion not diagnosed  1 = Pleural effusion diagnosed |  |
| Renal replacement therapy RRT^A^ | categorical | 0 = no RRT  1 = RRT |  |

^A^ Determined for day 1 to 14 individually

^B^ Not received, if less than 400 IE heparin and less than 40 mg enoxaparin were given on the day under consideration; received, else

^C^ Given at least once in the two weeks under consideration

^D^ High, if the virus load is higher than 1x10^6^ at least once in the two weeks under consideration; low, else

*List of all observed parameters divided into categories*
