# Supplementary material for: Lower blood pH as a strong prognostic factor for fatal outcomes in critically ill COVID-19 patients at an intensive care unit: A multivariable analysis
Source: PLoS One. 2021 Sep 29;16(9):e0258018. doi: 10.1371/journal.pone.0258018 (PMC8480873; doi:10.1371/journal.pone.0258018)
Supplement: S3 Table — (DOCX) [file pone.0258018.s004.docx]

| **Patient number** | **Event** | **Day of occurrence after admission to ICU** | **Outcome** | **Description of the event** |
| --- | --- | --- | --- | --- |
| #20 | ICH | Day 1 | Non-survivor (died day 7) | Severely cardiac pre-diseased patient (post ACVB), initial admission with suspected mitral valve endocarditis, manifestation of Covid-19 during treatment at IMC, ICH during treatment at IMC, admission to ICU after craniotomy. |
| #32 | ICH | Day 10 | Non-survivor (died day 10) | Large ICH with infaust prognosis, at the time of occurrence therapeutic anticoagulation with UFH 1,500 IU/h (PTT 65.7 s). |
| #45 | ICH | Day 1 | Non-survivor (died day 2) | Initially admitted already with large ICH with infaust prognosis. |
| #53 | ICH | Day 7 | Non-survivor (died day 7) | Small ICH, at the time of occurrence therapeutic anticoagulation with UFH 1,550 IU/h (PTT 75.2 s). Patient did not die due to ICH. |
| #52 | ICH | Day 2 | Survivor | Implantation of vvECMO in external hospital, subsequently therapeutic anticoagulation with UFH (dosage not known), on admission PTT 55.6 s, on CT scan bihemispheric minor hemorrhages. |
| #26 | Other bleeding complication | Day 7 | Non-survivor (died day 24) | Diffuse intra-abdominal hemorrhage under therapeutic anticoagulation with UFH 1,200 IU/h (PTT 74.4 s). |
| #47 | Other bleeding complication | Day 3 | Non-survivor (died day 21) | Esophageal variceal hemorrhage in decompensated liver cirrhosis CHILD C in known alcohol abuse with coagulopathy. |
| #1 | Other bleeding complication | Day 14 | Survivor | Diffuse but well-controlled bleeding in the oral-nasal cavity under therapeutic anticoagulation with LMWH. |
| #27 | Other bleeding complication | Day 5 | Survivor | Severely pre-diseased patient, post orthotopic heart transplant, chronic renal failure. Well controllable epistaxis with only prophylactic anticoagulation with UFH. |
| #44 | Other bleeding complication | Day 8 | Survivor | Pre-existing use of DOAK for previous pulmonary embolism. Acute lower gastrointestinal bleeding under UFH 1,300 IU/h (PTT 37.6 s). |

*Description of concrete events in patients suffering from intracerebral hemorrhage (ICH) or other bleeding complications. ACVB, aortocoronary venous bypass; IMC, intermediate care unit; ICU, intensive care unit; UFH; unfractured heparin; LMWH, low molecular weight heparin; PTT, partial thromboplastin time; DOAK, direct oral anticoagulants.*
