# Supplementary material for: Lower blood pH as a strong prognostic factor for fatal outcomes in critically ill COVID-19 patients at an intensive care unit: A multivariable analysis
Source: PLoS One. 2021 Sep 29;16(9):e0258018. doi: 10.1371/journal.pone.0258018 (PMC8480873; doi:10.1371/journal.pone.0258018)
Supplement: S4 Table — (DOCX) [file pone.0258018.s005.docx]

|  | survivors | | non-survivors | | 95% confidence intervall of mean difference | | | p-value |
| --- | --- | --- | --- | --- | --- | --- | --- | --- |
|  | mean value | standard deviation | mean value | standard deviation | mean | minimum | maximum |  |
| Heartrate HR (mean) | 86,565 | 9,666 | 91,048 | 11,188 | -4,483 | -10,573 | 1,608 | 2,305E-01 |
| Mean arterial pressure MAP (mean) | 81,665 | 6,331 | 74,124 | 5,867 | 7,541 | 4,145 | 10,936 | 4,884E-05* |
| Oxygen saturation SpO_2_ (mean) | 95,595 | 1,051 | 94,642 | 2,930 | 0,953 | -0,490 | 2,396 | 1,811E-01 |
| Norepinephrine (mean) | 0,265 | 0,235 | 0,612 | 0,513 | -0,347 | -0,603 | -0,091 | 2,279E-03 |
| Sufentanil (mean) | 60,317 | 28,893 | 57,419 | 26,680 | 2,898 | -12,561 | 18,358 | 6,123E-01 |
| Propofol (mean) | 76,355 | 67,102 | 117,011 | 89,299 | -40,656 | -87,913 | 6,601 | 1,676E-01 |
| Midazolam (mean) | 7,373 | 8,118 | 5,570 | 3,448 | 1,803 | -1,217 | 4,822 | 8,311E-01 |
| Ketamin (mean) | 85,697 | 76,141 | 113,947 | 98,550 | -28,250 | -80,670 | 24,170 | 4,313E-01 |
| pH (mean) | 7,422 | 0,029 | 7,344 | 0,055 | 0,079 | 0,051 | 0,106 | 5,473E-08* |
| Bicarbonate ion HCO_3_^-^ (mean) | 27,943 | 3,527 | 25,248 | 3,380 | 2,695 | 0,759 | 4,630 | 9,367E-03 |
| Base excess BE (mean) | 3,449 | 3,265 | -0,286 | 3,364 | 3,735 | 1,849 | 5,621 | 2,164E-04* |
| Chloride Cl^-^ (mean) | 108,568 | 3,675 | 108,898 | 4,567 | -0,330 | -2,779 | 2,118 | 7,689E-01 |
| Partial pressure of oxygen paO_2_ (mean) | 88,263 | 10,653 | 92,105 | 17,629 | -3,842 | -12,904 | 5,220 | 5,359E-01 |
| Partial pressure of carbon dioxid paCO_2_ (mean) | 43,895 | 5,224 | 49,158 | 8,030 | -5,263 | -9,432 | -1,094 | 9,141E-03 |
| Hemoglobin Hb (mean) | 9,821 | 1,295 | 9,234 | 1,102 | 0,587 | -0,069 | 1,244 | 6,717E-02 |
| Lactate (mean) | 10,792 | 3,308 | 15,899 | 14,507 | -5,107 | -12,161 | 1,947 | 2,061E-01 |
| Troponin (mean) | 35,387 | 45,641 | 246,863 | 730,710 | -211,476 | -563,906 | 140,954 | 2,781E-04* |
| Glomeralur filtration rate GFR (mean) | 76,100 | 30,325 | 50,368 | 26,056 | 25,732 | 10,254 | 41,210 | 3,615E-03 |
| Creatinine (mean) | 1,383 | 1,275 | 1,715 | 0,706 | -0,332 | -0,850 | 0,186 | 1,418E-02 |
| Urea (mean) | 68,500 | 30,113 | 87,053 | 41,895 | -18,553 | -40,554 | 3,449 | 7,617E-02 |
| Aspartate transaminase AST (mean) | 101,974 | 55,881 | 228,895 | 279,870 | -126,920 | -262,751 | 8,910 | 3,873E-02 |
| Alanine transaminase ALT (mean) | 81,775 | 62,083 | 91,263 | 79,669 | -9,488 | -51,934 | 32,958 | 8,439E-01 |
| International Normalized Ratio INR (mean) | 1,139 | 0,180 | 1,206 | 0,265 | -0,068 | -0,206 | 0,070 | 1,889E-01 |
| Lactate dehydrogenase LDH (mean) | 441,150 | 177,502 | 450,611 | 156,297 | -9,461 | -103,297 | 84,374 | 7,359E-01 |
| C-reactive protein CRP (mean) | 152,033 | 75,630 | 207,695 | 96,378 | -55,662 | -107,079 | -4,245 | 3,630E-02 |
| Procalcitonin PCT (mean) | 2,637 | 7,856 | 9,932 | 15,868 | -7,294 | -15,265 | 0,677 | 1,309E-05* |
| White blood cells WBC (mean) | 11,466 | 6,464 | 19,906 | 21,965 | -8,441 | -19,184 | 2,303 | 8,053E-03 |
| Ferritin (mean) | 2626,425 | 2427,846 | 4357,632 | 4719,596 | -1731,207 | -4109,717 | 647,304 | 1,181E-01 |
| D-dimers (mean) | 7,103 | 7,609 | 5,232 | 4,477 | 1,871 | -1,300 | 5,042 | 6,963E-01 |
| Platelets (mean) | 299,825 | 130,229 | 265,526 | 157,358 | 34,299 | -50,561 | 119,158 | 1,631E-01 |
| Lymphocytes (mean) | 1,592 | 2,146 | 6,413 | 23,268 | -4,821 | -16,052 | 6,410 | 3,077E-01 |
| Interleucin 6 (mean) | 289,310 | 389,256 | 652,974 | 879,755 | -363,663 | -802,324 | 74,998 | 2,971E-03 |
| Prone position (mean) | 4,125 | 3,321 | 5,765 | 4,261 | -1,640 | -3,910 | 0,630 | 1,736E-01 |
| Therapeutic intervention scoring system TISS (mean) | 14,282 | 3,748 | 17,579 | 3,372 | -3,297 | -5,277 | -1,317 | 1,888E-04* |
| Simplified Acute Physiology Score SAPS (mean) | 38,769 | 9,189 | 50,421 | 11,848 | -11,652 | -17,974 | -5,330 | 1,448E-04* |
| Fraction of inspired oxygen FiO_2_ (mean) | 49,981 | 8,193 | 56,491 | 17,000 | -6,510 | -15,058 | 2,039 | 1,042E-01 |
| Positive endexpiratory pressure PEEP (mean) | 12,681 | 1,840 | 12,879 | 2,894 | -0,198 | -1,699 | 1,302 | 2,801E-01 |
| Driving Pressure (mean) | 12,892 | 2,159 | 13,803 | 2,078 | -0,911 | -2,166 | 0,345 | 7,057E-02 |
| Tidal volume VT (mean) | 452,934 | 122,136 | 468,771 | 128,427 | -15,837 | -88,231 | 56,557 | 4,922E-01 |
| Oxygenation ratio (Horovitz) (mean) | 189,405 | 39,489 | 190,789 | 81,752 | -1,384 | -42,500 | 39,732 | 6,037E-01 |
| Heartrate HR (minimum) | 72,158 | 10,874 | 77,883 | 13,530 | -5,724 | -12,977 | 1,528 | 1,335E-01 |
| Heartrate HR (maximum) | 102,362 | 8,966 | 105,175 | 12,535 | -2,813 | -9,391 | 3,764 | 5,679E-01 |
| Mean arterial pressure MAP (minimum) | 92,772 | 9,447 | 84,242 | 9,215 | 8,530 | 3,281 | 13,778 | 3,147E-03 |
| Oxygen saturation SpO_2_ (minimum) | 93,406 | 1,371 | 91,286 | 5,486 | 2,119 | -0,553 | 4,791 | 2,536E-01 |
| Norepinephrine (maximum) | 0,731 | 0,528 | 1,504 | 0,997 | -0,773 | -1,277 | -0,269 | 8,810E-04 |
| Sufentanil (maximum) | 89,908 | 33,788 | 82,988 | 25,165 | 6,920 | -8,910 | 22,749 | 2,336E-01 |
| Propofol (maximum) | 156,890 | 97,489 | 214,018 | 132,337 | -57,128 | -126,916 | 12,661 | 2,182E-01 |
| Midazolam (maximum) | 11,791 | 9,992 | 12,092 | 5,365 | -0,301 | -4,312 | 3,711 | 7,262E-01 |
| Ketamin (maximum) | 165,846 | 106,680 | 185,087 | 118,751 | -19,241 | -84,488 | 46,007 | 4,067E-01 |
| pH (minimum) | 7,350 | 0,055 | 7,250 | 0,059 | 0,100 | 0,067 | 0,132 | 1,297E-07* |
| pH (maximum) | 7,487 | 0,031 | 7,422 | 0,065 | 0,064 | 0,032 | 0,097 | 4,231E-05* |
| Bicarbonate ion HCO_3_^-^ (minimum) | 23,245 | 4,227 | 21,942 | 3,311 | 1,302 | -0,736 | 3,341 | 1,513E-01 |
| Bicarbonate ion HCO_3_^-^ (maximum) | 32,184 | 4,164 | 28,369 | 4,426 | 3,814 | 1,354 | 6,275 | 2,884E-03 |
| Base excess BE (minimum) | -0,834 | 3,600 | -4,424 | 3,252 | 3,590 | 1,692 | 5,488 | 6,829E-04 |
| Base excess BE (maximum) | 7,480 | 3,839 | 3,325 | 4,176 | 4,156 | 1,848 | 6,463 | 3,317E-04* |
| Chloride Cl^-^ (minimum) | 102,344 | 3,633 | 103,756 | 5,253 | -1,412 | -4,154 | 1,330 | 6,788E-01 |
| Chloride Cl^-^ (maximum) | 115,188 | 5,230 | 113,542 | 4,293 | 1,646 | -0,948 | 4,240 | 1,191E-01 |
| Partial pressure of oxygen paO_2_ (minimum) | 72,026 | 7,134 | 74,526 | 18,524 | -2,500 | -11,667 | 6,667 | 1,751E-01 |
| Partial pressure of oxygen paO_2_ (maximum) | 119,053 | 35,069 | 120,579 | 36,494 | -1,526 | -22,079 | 19,026 | 9,032E-01 |
| Partial pressure of carbon dioxid paCO_2_ (minimum) | 35,053 | 5,671 | 39,211 | 8,087 | -4,158 | -8,406 | 0,090 | 3,507E-02 |
| Partial pressure of carbon dioxid paCO_2_ (maximum) | 52,684 | 9,242 | 62,947 | 19,665 | -10,263 | -20,121 | -0,405 | 5,540E-03 |
| Hemoglobin Hb (minimum) | 8,529 | 1,355 | 8,003 | 0,980 | 0,526 | -0,099 | 1,150 | 4,759E-02 |
| Lactate (maximum) | 17,334 | 6,173 | 27,935 | 26,034 | -10,601 | -23,269 | 2,067 | 3,314E-01 |
| Troponin (maximum) | 66,410 | 96,616 | 369,579 | 1035,340 | -303,169 | -802,942 | 196,605 | 1,412E-03 |
| Glomeralur filtration rate GFR (minimum) | 57,875 | 32,832 | 31,000 | 20,347 | 26,875 | 12,871 | 40,879 | 2,645E-03 |
| Creatinine (maximum) | 2,166 | 2,812 | 2,674 | 1,366 | -0,508 | -1,597 | 0,581 | 1,062E-02 |
| Urea (maximum) | 113,300 | 61,853 | 138,263 | 80,073 | -24,963 | -67,553 | 17,627 | 2,306E-01 |
| Aspartate transaminase AST (maximum) | 187,205 | 138,808 | 403,368 | 427,628 | -216,163 | -426,080 | -6,246 | 1,113E-01 |
| Alanine transaminase ALT (maximum) | 167,625 | 174,179 | 180,684 | 173,216 | -13,059 | -111,149 | 85,030 | 9,647E-01 |
| International Normalized Ratio INR (minimum) | 1,000 | 0,151 | 1,049 | 0,177 | -0,049 | -0,145 | 0,047 | 7,304E-02 |
| International Normalized Ratio INR (maximum) | 1,383 | 0,410 | 1,515 | 0,669 | -0,131 | -0,475 | 0,212 | 1,464E-01 |
| Lactate dehydrogenase LDH (maximum) | 696,400 | 432,036 | 694,944 | 470,866 | 1,456 | -264,545 | 267,456 | 6,982E-01 |
| C-reactive protein CRP (maximum) | 293,660 | 124,261 | 330,105 | 112,476 | -36,445 | -102,052 | 29,161 | 2,338E-01 |
| Procalcitonin PCT (maximum) | 6,491 | 19,687 | 18,545 | 29,211 | -12,053 | -27,241 | 3,134 | 4,056E-05* |
| White blood cells WBC (minimum) | 6,973 | 3,442 | 12,653 | 14,553 | -5,680 | -12,761 | 1,401 | 1,876E-02 |
| White blood cells WBC (maximum) | 16,997 | 10,326 | 28,191 | 30,943 | -11,194 | -26,392 | 4,004 | 1,562E-02 |
| Ferritin (maximum) | 5158,400 | 6759,063 | 7340,789 | 8908,861 | -2182,389 | -6905,168 | 2540,389 | 1,771E-01 |
| D-dimers (maximum) | 13,454 | 12,075 | 9,793 | 9,491 | 3,661 | -2,175 | 9,496 | 2,920E-01 |
| Platelets (minimum) | 195,450 | 106,256 | 173,368 | 129,747 | 22,082 | -47,734 | 91,897 | 2,062E-01 |
| Platelets (maximum) | 426,025 | 163,656 | 355,737 | 182,338 | 70,288 | -29,875 | 170,452 | 4,406E-02 |
| Lymphocytes (maximum) | 2,298 | 2,417 | 8,483 | 30,329 | -6,186 | -20,820 | 8,448 | 2,535E-01 |
| Interleucin 6 (maximum) | 1224,228 | 2439,065 | 1416,984 | 1854,478 | -192,756 | -1355,450 | 969,938 | 7,898E-02 |
| Therapeutic intervention scoring system TISS (maximum) | 20,300 | 6,988 | 22,579 | 4,925 | -2,279 | -5,456 | 0,898 | 9,715E-02 |
| Simplified Acute Physiology Score SAPS (maximum) | 47,150 | 13,114 | 61,158 | 14,901 | -14,008 | -22,155 | -5,861 | 1,714E-04* |
| Fraction of inspired oxygen FiO_2_ (minimum) | 35,946 | 4,808 | 44,553 | 17,702 | -8,607 | -17,255 | 0,042 | 8,044E-02 |
| Fraction of inspired oxygen FiO_2_ (maximum) | 74,409 | 12,993 | 75,408 | 17,540 | -0,999 | -10,324 | 8,327 | 4,922E-01 |
| Positive endexpiratory pressure PEEP (minimum) | 9,069 | 2,754 | 10,928 | 3,315 | -1,858 | -3,663 | -0,054 | 3,509E-02 |
| Positive endexpiratory pressure PEEP (maximum) | 15,303 | 1,369 | 15,112 | 2,911 | 0,191 | -1,270 | 1,652 | 6,279E-01 |
| Driving Pressure (maximum) | 15,406 | 2,430 | 16,290 | 2,883 | -0,884 | -2,537 | 0,769 | 3,070E-01 |
| Tidal volume VT (minimum) | 355,006 | 102,420 | 383,503 | 112,642 | -28,496 | -91,211 | 34,219 | 3,186E-01 |
| Tidal volume VT (maximum) | 591,697 | 182,256 | 552,357 | 155,911 | 39,340 | -54,835 | 133,516 | 8,103E-01 |
| Oxygenation ratio (Horovitz) (minimum) | 120,108 | 35,385 | 131,000 | 52,628 | -10,892 | -38,409 | 16,626 | 7,739E-01 |

*Overview of all analyzed parameters that were recorded on a daily basis: mean, maximum and minimum refer to all values per patient during the observation period. Significant differences between the two groups are marked with an asterisk.*
