# Supplementary material for: Lower blood pH as a strong prognostic factor for fatal outcomes in critically ill COVID-19 patients at an intensive care unit: A multivariable analysis
Source: PLoS One. 2021 Sep 29;16(9):e0258018. doi: 10.1371/journal.pone.0258018 (PMC8480873; doi:10.1371/journal.pone.0258018)
Supplement: S5 Table — (DOCX) [file pone.0258018.s006.docx]

|  | OR (95%-CI) | p-value | c-index (AUC) |
| --- | --- | --- | --- |
| MAPmean | 1.264 (1.100 – 1.453) | 0.001 | 0.816 |
| pHmean | 1.608 (1.253 – 2.064) | <0.001 | 0.901 |
| pHmax | 1.403 (1.148 – 1.714) | 0.001 | 0.816 |
| pHmin | 1.318 (1.148 – 1.514) | <0.001 | 0.893 |
| BEmean | 1.408 (1.144 – 1.733) | 0.001 | 0.790 |
| BEmax | 1.305 (1.101 – 1.547) | 0.002 | 0.782 |
| Troponin Tmean | 1.106 (1.001 – 1.031) | 0.040 | 0.787 |

***Univariable logistic regression models on non-surviving.*** *MAPmean, mean MAP during the 14-day observation period for each patient; pHmean/pHmax/pHmin, mean, maximum and minimum blood pH during the 14-day observation period for each patient; BEmean/BEmax, mean, maximum BE during the 14-day observation period for each patient; Troponin Tmean, mean troponin T during the 14-day observation period for each patient; OR, odds ratio; CI confidence interval; AUC, area under the curve. For better comparability of OR and c-index between the single models all parameters except Troponin Tmean were multiplied with -1; in addition, pHmean, pHmax and pHmin were multiplied with 100 for calculation of the regression model.*
