# Supplementary material for: Lower blood pH as a strong prognostic factor for fatal outcomes in critically ill COVID-19 patients at an intensive care unit: A multivariable analysis
Source: PLoS One. 2021 Sep 29;16(9):e0258018. doi: 10.1371/journal.pone.0258018 (PMC8480873; doi:10.1371/journal.pone.0258018)
Supplement: S6 Table — (DOCX) [file pone.0258018.s007.docx]

|  | OR (95%-CI) | p-value | c-index (AUC) |
| --- | --- | --- | --- |
| MAPmean | 1.269 (1.062 – 1.516) | 0.009 | 0.945 |
| pHmin | 1.329 (1.131 – 1.562) | 0.001 |  |

***Multivariable*** ***logistic regression model on non-surviving.*** *MAPmean, mean MAP during the 14-day observation period; pHmin, minimum blood pH during the 14-day observation period for each patient; OR, odds ratio; CI confidence interval; AUC, area under the curve.*
