# Supplementary material for: Lower blood pH as a strong prognostic factor for fatal outcomes in critically ill COVID-19 patients at an intensive care unit: A multivariable analysis
Source: PLoS One. 2021 Sep 29;16(9):e0258018. doi: 10.1371/journal.pone.0258018 (PMC8480873; doi:10.1371/journal.pone.0258018)
Supplement: S7 Table — (DOCX) [file pone.0258018.s008.docx]

|  |  |  |  | Calculation with all available values | | |  | Calculation without values for the last 3 days before death in non-survivors who had died during the observation period | | |
| --- | --- | --- | --- | --- | --- | --- | --- | --- | --- | --- |
| Patient | Outcome | Time of death (days since admission) |  | MAPmean | pHmin | P_all_ |  | MAPmean | pHmin | P_-3_ |
| #1 | 0 |  |  | 77,09 | 7,44 | 0,01 |  | 77,09 | 7,44 | 0,01 |
| #2 | 0 |  |  | 75,53 | 7,22 | 0,9 |  | 75,53 | 7,22 | 0,9 |
| #3 | 0 |  |  | 77,96 | 7,39 | 0,04 |  | 77,96 | 7,39 | 0,04 |
| #4 | 0 |  |  | 80,12 | 7,3 | 0,24 |  | 80,12 | 7,3 | 0,24 |
| #5 | 0 |  |  | 82,01 | 7,19 | 0,82 |  | 82,01 | 7,19 | 0,82 |
| #6 | 0 |  |  | 84,91 | 7,38 | 0,01 |  | 84,91 | 7,38 | 0,01 |
| #7 | 1 | 10 |  | 71,63 | 7,21 | 0,97 |  | 75,79 | 7,21 | 0,92 |
| #8 | 0 |  |  | 86,72 | 7,31 | 0,05 |  | 86,72 | 7,31 | 0,05 |
| #9 | 0 |  |  | 86,13 | 7,35 | 0,02 |  | 86,13 | 7,35 | 0,02 |
| #10 | 1 | 11 |  | 77,28 | 7,31 | 0,32 |  | 81,41 | 7,43 | 0,01 |
| #11 | 0 |  |  | 74,43 | 7,36 | 0,18 |  | 74,43 | 7,36 | 0,18 |
| #12 | 1 | 95 |  | 82,62 | 7,23 | 0,56 |  | 82,62 | 7,23 | 0,56 |
| #13 | 1 | 12 |  | 70,4 | 7,2 | 0,98 |  | 73,34 | 7,24 | 0,9 |
| #14 | 1 | 27 |  | 76,21 | 7,23 | 0,86 |  | 76,21 | 7,23 | 0,86 |
| #15 | 1 | 6 |  | 73,79 | 7,12 | 1 |  | 78,28 | 7,36 | 0,08 |
| #16 | 0 |  |  | 87,95 | 7,3 | 0,05 |  | 87,95 | 7,3 | 0,05 |
| #17 | 0 |  |  | 82,93 | 7,35 | 0,04 |  | 82,93 | 7,35 | 0,04 |
| #18 | 0 |  |  | 69,96 | 7,31 | 0,73 |  | 69,96 | 7,31 | 0,73 |
| #19 | 1 | 45 |  | 73,6 | 7,24 | 0,89 |  | 73,6 | 7,24 | 0,89 |
| #20 | 1 | 7 |  | 62,32 | 7,32 | 0,93 |  | 66,43 | 7,33 | 0,78 |
| #21 | 1 | 17 |  | 79,03 | 7,29 | 0,36 |  | 79,03 | 7,29 | 0,36 |
| #22 | 0 |  |  | 77,91 | 7,38 | 0,05 |  | 77,91 | 7,38 | 0,05 |
| #23 | 0 |  |  | 75,76 | 7,41 | 0,04 |  | 75,76 | 7,41 | 0,04 |
| #24 | 0 |  |  | 84,08 | 7,38 | 0,01 |  | 84,08 | 7,38 | 0,01 |
| #25 | 0 |  |  | 76,74 | 7,4 | 0,04 |  | 76,74 | 7,4 | 0,04 |
| #26 | 1 | 24 |  | 77,12 | 7,27 | 0,61 |  | 77,12 | 7,27 | 0,61 |
| #27 | 0 |  |  | 99,24 | 7,32 | 0 |  | 99,24 | 7,32 | 0 |
| #28 | 1 | 25 |  | 69,34 | 7,3 | 0,81 |  | 69,34 | 7,3 | 0,81 |
| #29 | 1 | 126 |  | 76,53 | 7,31 | 0,36 |  | 76,53 | 7,31 | 0,36 |
| #30 | 0 |  |  | 82,4 | 7,35 | 0,04 |  | 82,4 | 7,35 | 0,04 |
| #31 | 0 |  |  | 85,12 | 7,41 | 0 |  | 85,12 | 7,41 | 0 |
| #32 | 1 | 9 |  | 74,57 | 7,28 | 0,68 |  | 77,4 | 7,28 | 0,52 |
| #33 | 0 |  |  | 83,46 | 7,37 | 0,02 |  | 83,46 | 7,37 | 0,02 |
| #34 | 0 |  |  | 94,07 | 7,43 | 0 |  | 94,07 | 7,43 | 0 |
| #35 | 0 |  |  | 83,7 | 7,35 | 0,03 |  | 83,7 | 7,35 | 0,03 |
| #36 | 0 |  |  | 79,95 | 7,32 | 0,16 |  | 79,95 | 7,32 | 0,16 |
| #37 | 0 |  |  | 81,26 | 7,34 | 0,07 |  | 81,26 | 7,34 | 0,07 |
| #38 | 0 |  |  | 80,06 | 7,34 | 0,09 |  | 80,06 | 7,34 | 0,09 |
| #39 | 0 |  |  | 81,37 | 7,26 | 0,43 |  | 81,37 | 7,26 | 0,43 |
| #40 | 0 |  |  | 80,26 | 7,27 | 0,42 |  | 80,26 | 7,27 | 0,42 |
| #41 | 0 |  |  | 90,57 | 7,27 | 0,06 |  | 90,57 | 7,27 | 0,06 |
| #42 | 0 |  |  | 92,98 | 7,38 | 0 |  | 92,98 | 7,38 | 0 |
| #43 | 1 | 20 |  | 87,94 | 7,22 | 0,33 |  | 87,94 | 7,22 | 0,33 |
| #44 | 0 |  |  | 85,93 | 7,34 | 0,03 |  | 85,93 | 7,34 | 0,03 |
| #45 | 1 | 2 |  | 69,71 | 7,25 | 0,94 |  |  |  |  |
| #46 | 0 |  |  | 78,14 | 7,36 | 0,09 |  | 78,14 | 7,36 | 0,09 |
| #47 | 1 | 32 |  | 75 | 7,36 | 0,16 |  | 75 | 7,36 | 0,16 |
| #48 | 1 | 2 |  | 70,34 | 7,16 | 0,99 |  |  |  |  |
| #49 | 0 |  |  | 90,26 | 7,39 | 0 |  | 90,26 | 7,39 | 0 |
| #50 | 0 |  |  | 75,1 | 7,4 | 0,06 |  | 75,1 | 7,4 | 0,06 |
| #51 | 0 |  |  | 74,18 | 7,37 | 0,15 |  | 74,18 | 7,37 | 0,15 |
| #52 | 0 |  |  | 74,05 | 7,42 | 0,04 |  | 74,05 | 7,42 | 0,04 |
| #53 | 1 | 7 |  | 65,03 | 7,21 | 0,99 |  | 65,15 | 7,25 | 0,98 |
| #54 | 0 |  |  | 74,08 | 7,36 | 0,2 |  | 74,08 | 7,36 | 0,2 |
| #55 | 0 |  |  | 72,16 | 7,4 | 0,11 |  | 72,16 | 7,4 | 0,11 |
| #56 | 0 |  |  | 80,33 | 7,33 | 0,12 |  | 80,33 | 7,33 | 0,12 |
| #57 | 0 |  |  | 82,35 | 7,38 | 0,02 |  | 82,35 | 7,38 | 0,02 |
| #58 | 0 |  |  | 85,34 | 7,36 | 0,02 |  | 85,34 | 7,36 | 0,02 |
| #59 | 1 | 24 |  | 75,9 | 7,24 | 0,83 |  | 75,9 | 7,24 | 0,83 |

*Calculation of the probability P for a fatal outcome: P_all_: All available values for all patients are considered; P_-3_: Values for the last 3 days before death are excluded in non-survivors who had died within the observation period. P values are marked orange in cases of P>0.5 despite surviving and blue in cases of P<0.5 despite non-surviving. P_-3_ cannot be calculated in two cases as these had already died on the 2nd day of observation. 0, survived; 1, died. MAPmean, mean MAP during the 14-day observation period for each patient; pHmin, minimum blood pH during the 14-day observation period for each patient.*
