# Supplementary material for: Lower blood pH as a strong prognostic factor for fatal outcomes in critically ill COVID-19 patients at an intensive care unit: A multivariable analysis
Source: PLoS One. 2021 Sep 29;16(9):e0258018. doi: 10.1371/journal.pone.0258018 (PMC8480873; doi:10.1371/journal.pone.0258018)
Supplement: S1 Fig — (DOCX) [file pone.0258018.s009.docx]

*Distribution of age*

*Distribution of body-massindex (BMI)*


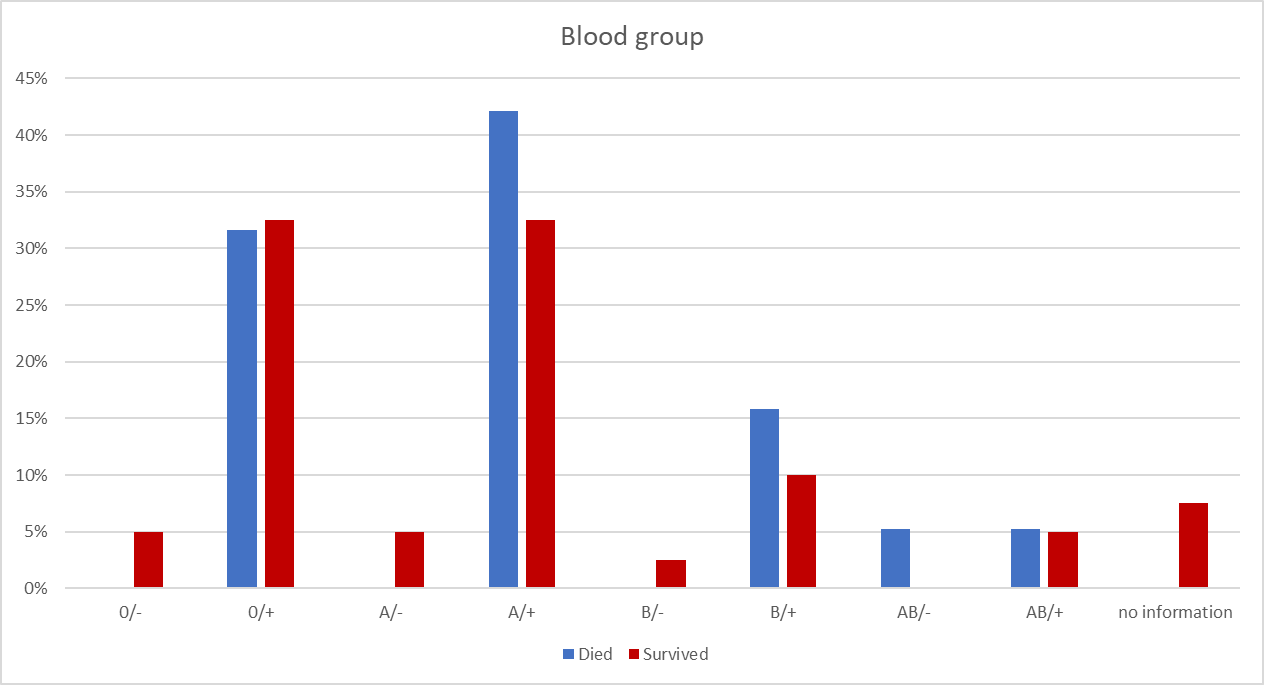


*Distribution of the blood groups*


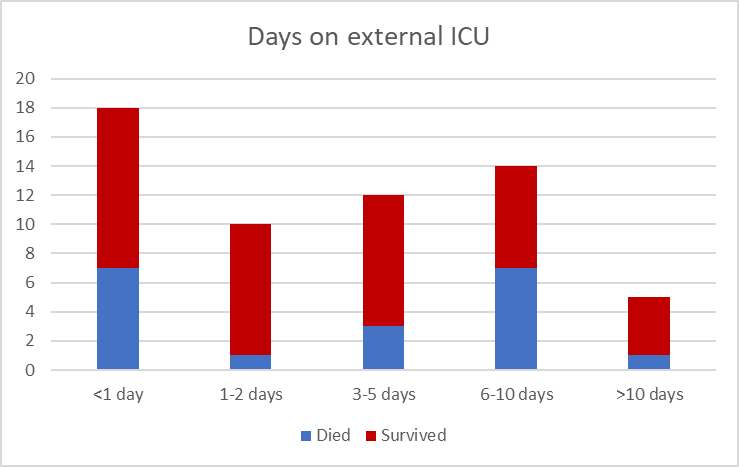


*Number of days treated at an external intensive care unit (ICU) before transfer to an ICU at the University Medical Center Regensburg*
