# Supplementary material for: Lower blood pH as a strong prognostic factor for fatal outcomes in critically ill COVID-19 patients at an intensive care unit: A multivariable analysis
Source: PLoS One. 2021 Sep 29;16(9):e0258018. doi: 10.1371/journal.pone.0258018 (PMC8480873; doi:10.1371/journal.pone.0258018)
Supplement: S3 Fig — (DOCX) [file pone.0258018.s011.docx]

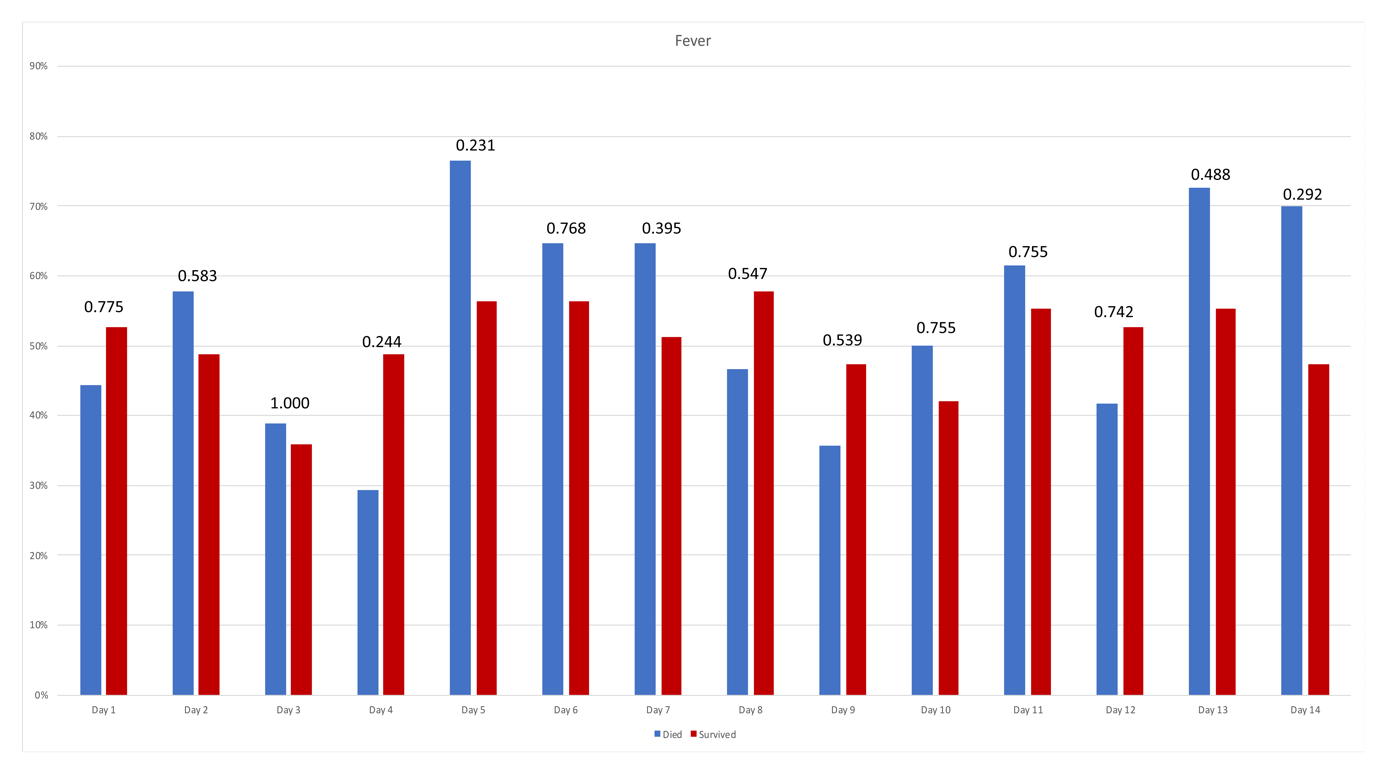


*Frequencies of ‘days of fever’ (daily peak temperature ≥38°C) during the observation period; p-values for comparison between the two groups are stated above the bars.*

***
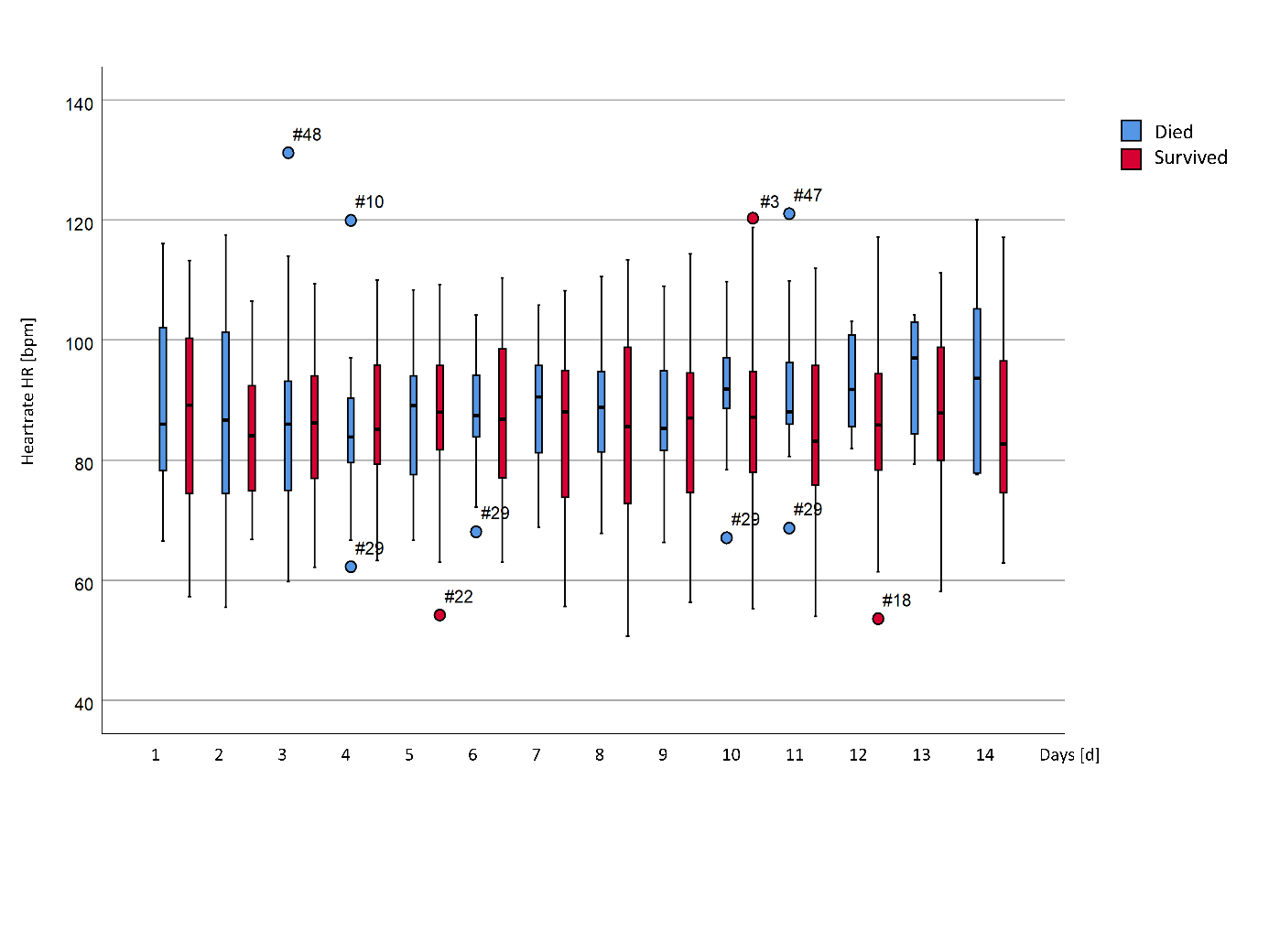
****Daily mean heartrate (HR). Significant differences between the two groups are marked with an asterisk in the legend of the x-axis.*


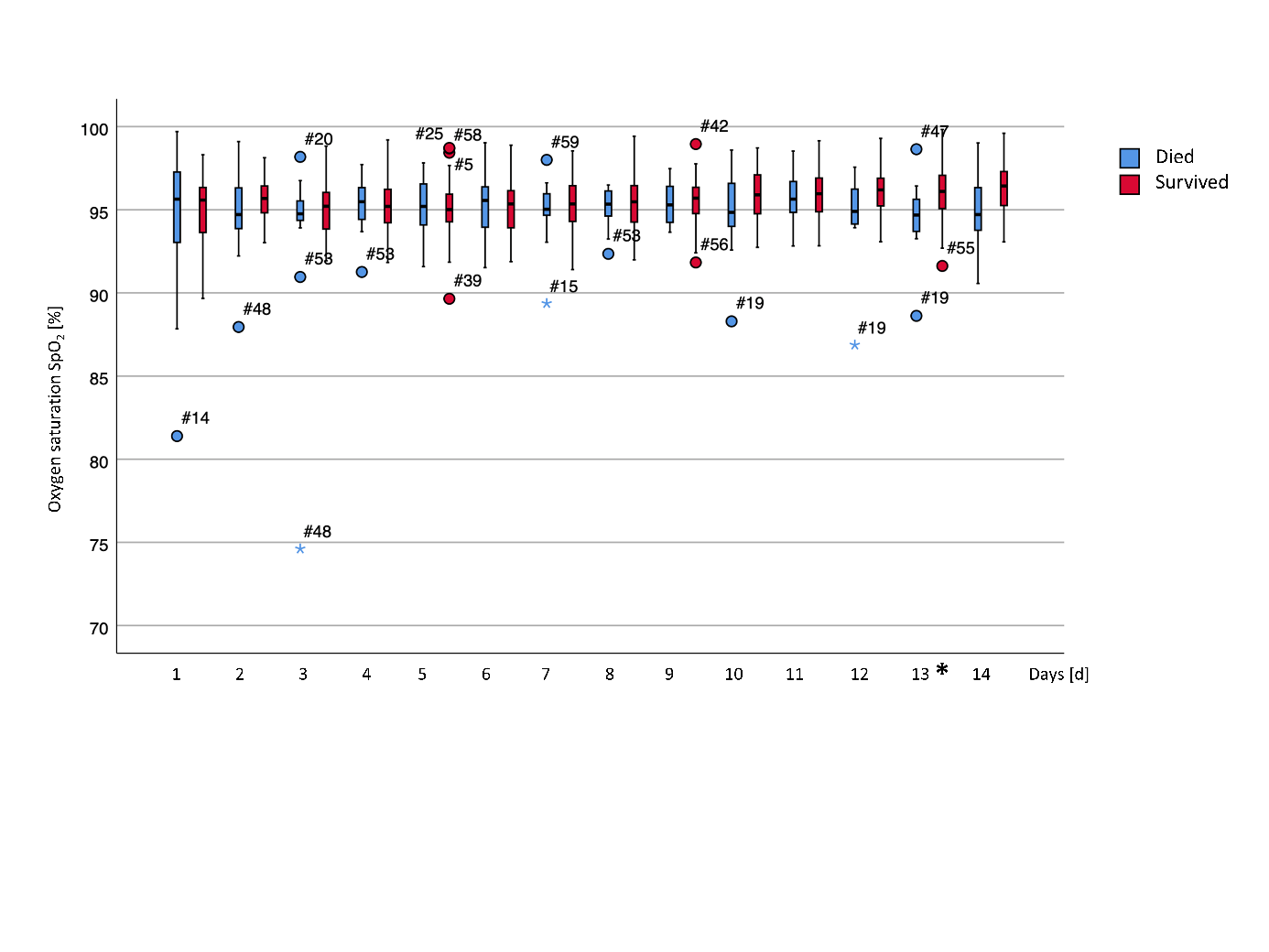


*Daily mean oxygen saturation (SpO_2_). Significant differences between the two groups are marked with an asterisk in the legend of the x-axis.*


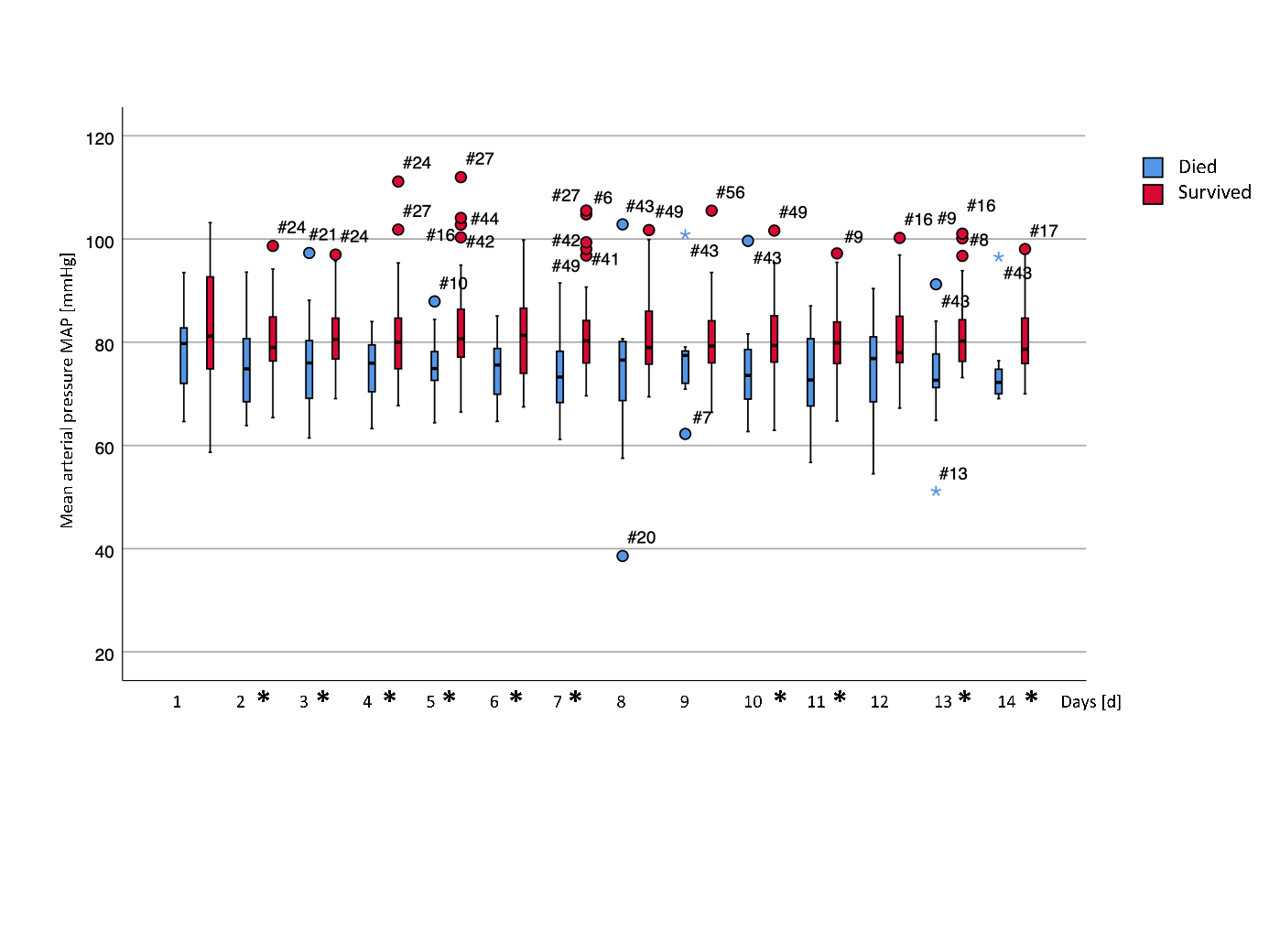


*Daily mean MAP (mean arterial pressure). Significant differences between the two groups are marked with an asterisk in the legend of the x-axis.*
