# Supplementary material for: Lower blood pH as a strong prognostic factor for fatal outcomes in critically ill COVID-19 patients at an intensive care unit: A multivariable analysis
Source: PLoS One. 2021 Sep 29;16(9):e0258018. doi: 10.1371/journal.pone.0258018 (PMC8480873; doi:10.1371/journal.pone.0258018)
Supplement: S4 Fig — (DOCX) [file pone.0258018.s012.docx]

**
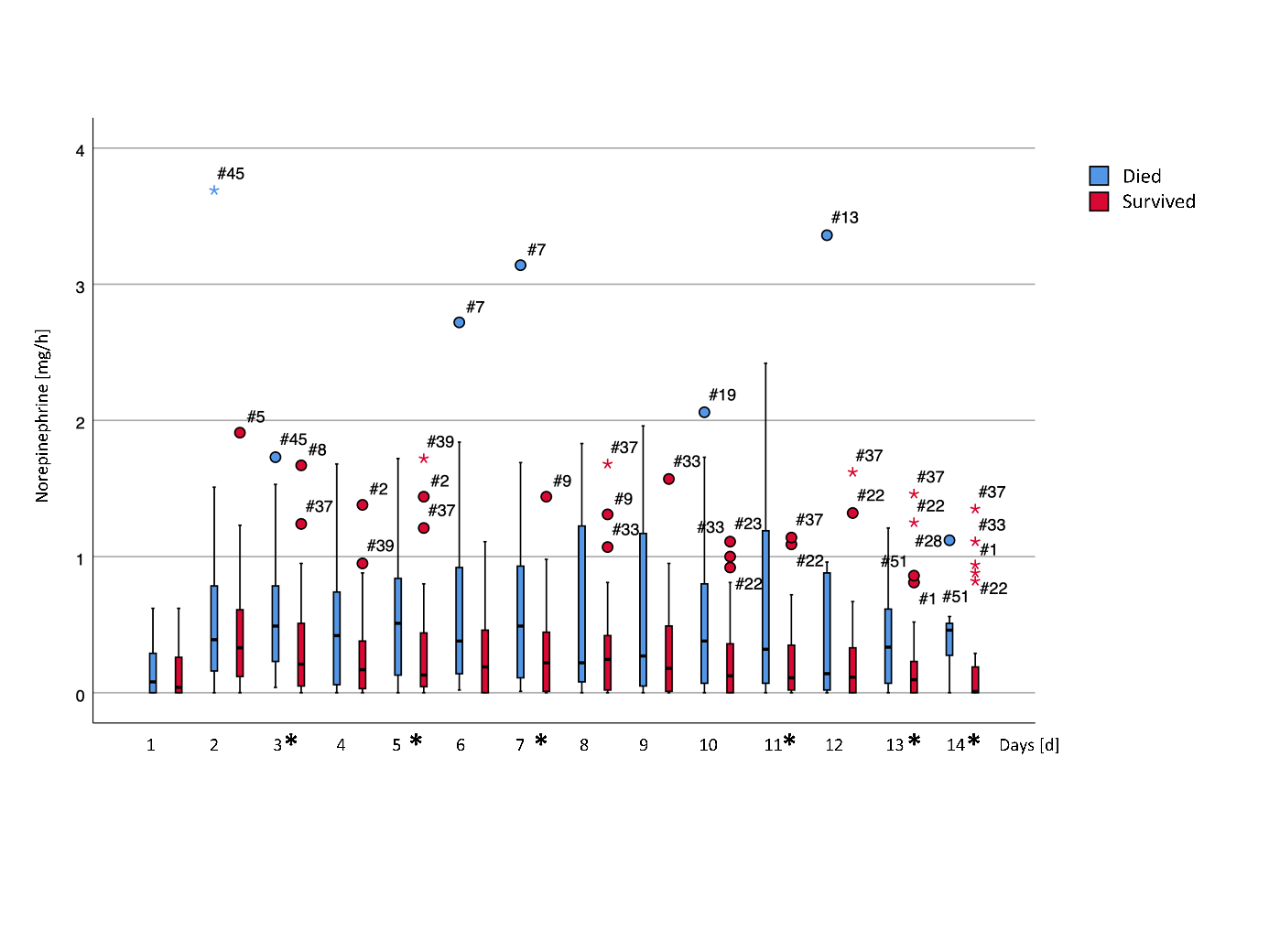
**

*Daily mean hourly dosage of norepinephrine. Significant differences between the two groups are marked with an asterisk in the legend of the x-axis.*


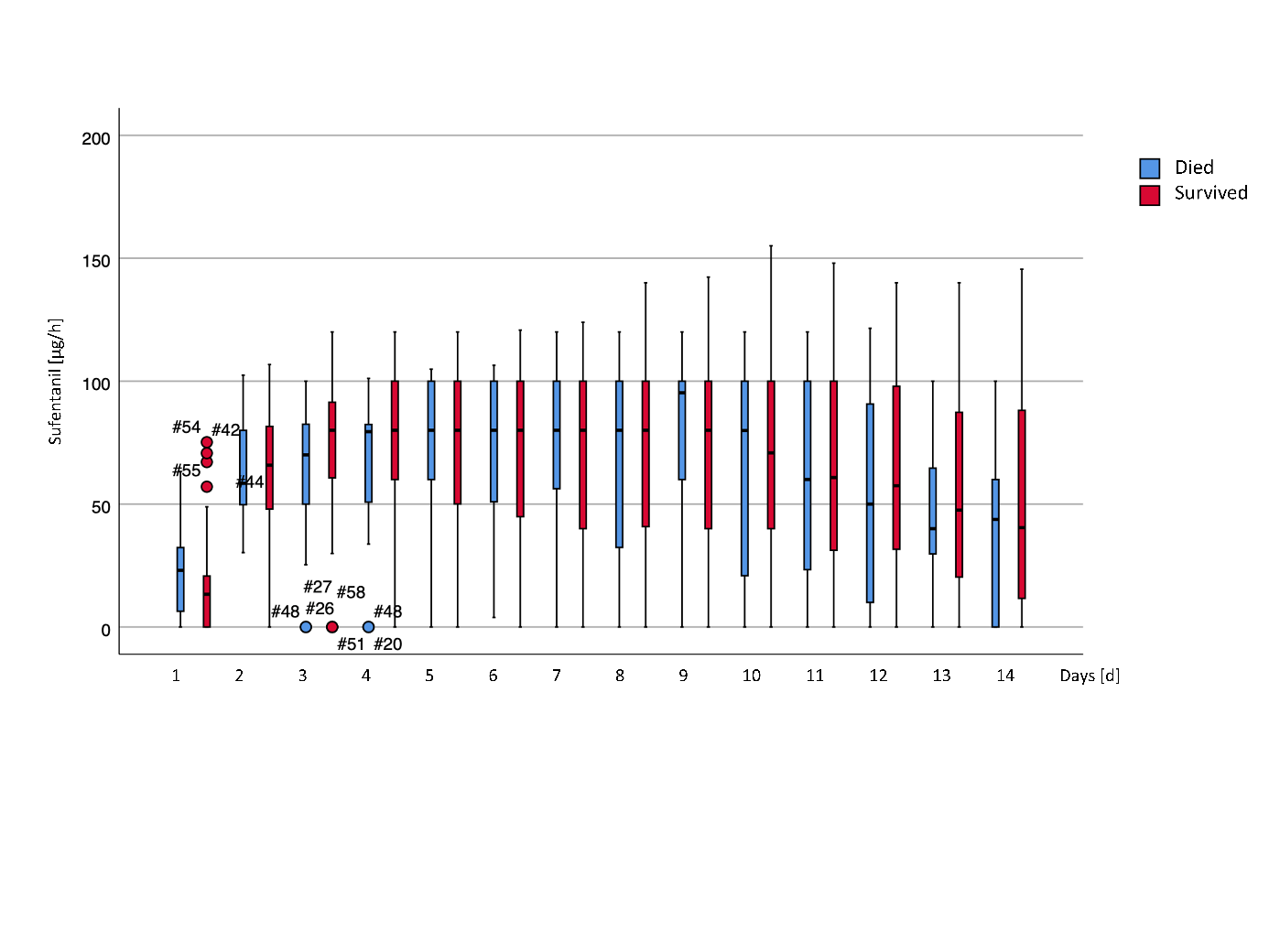


*Daily mean hourly dosage of sufentanil. Significant differences between the two groups are marked with an asterisk in the legend of the x-axis.*


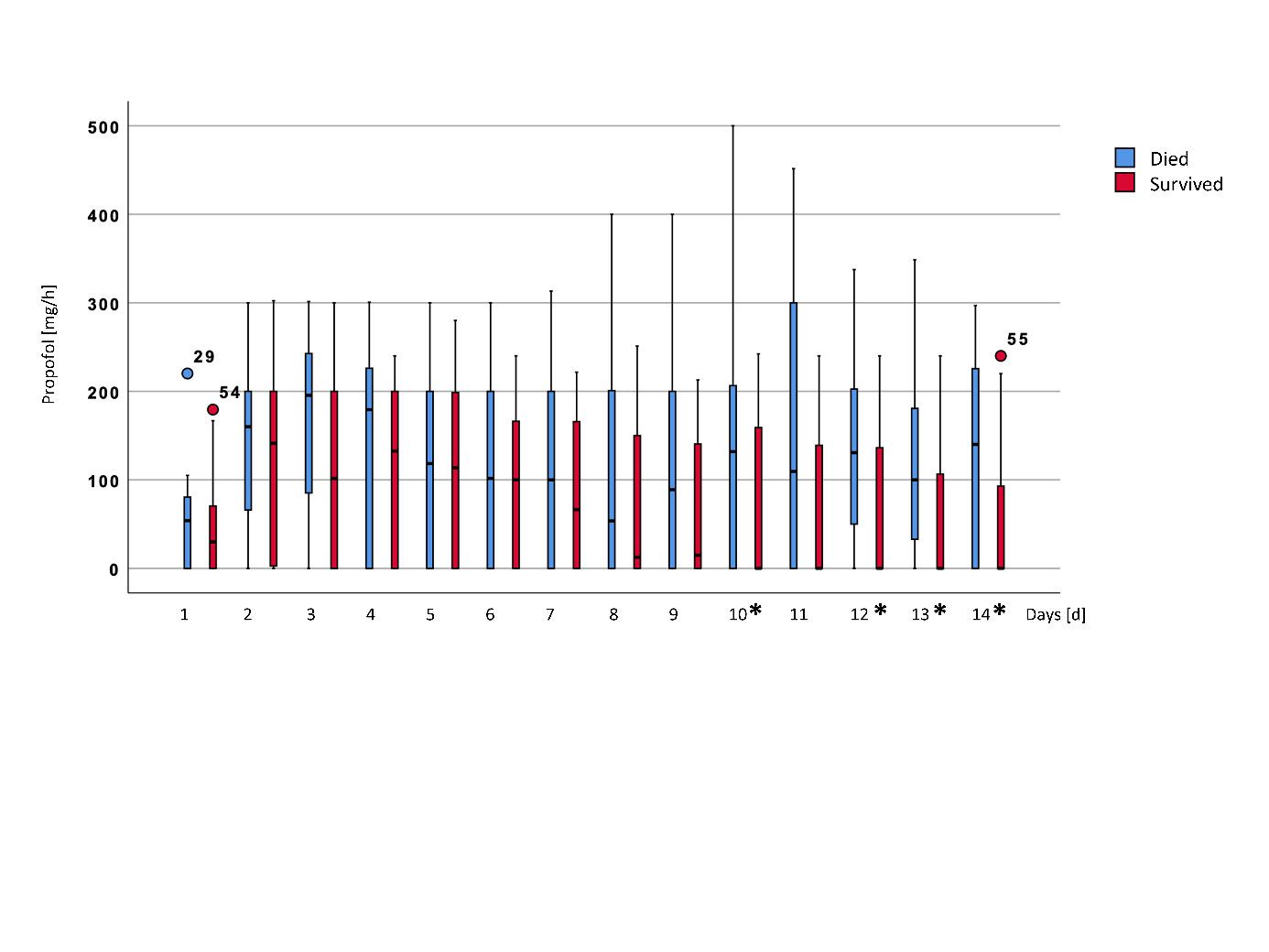


*Daily mean hourly dosage of propofol. Significant differences between the two groups are marked with an asterisk in the legend of the x-axis.*


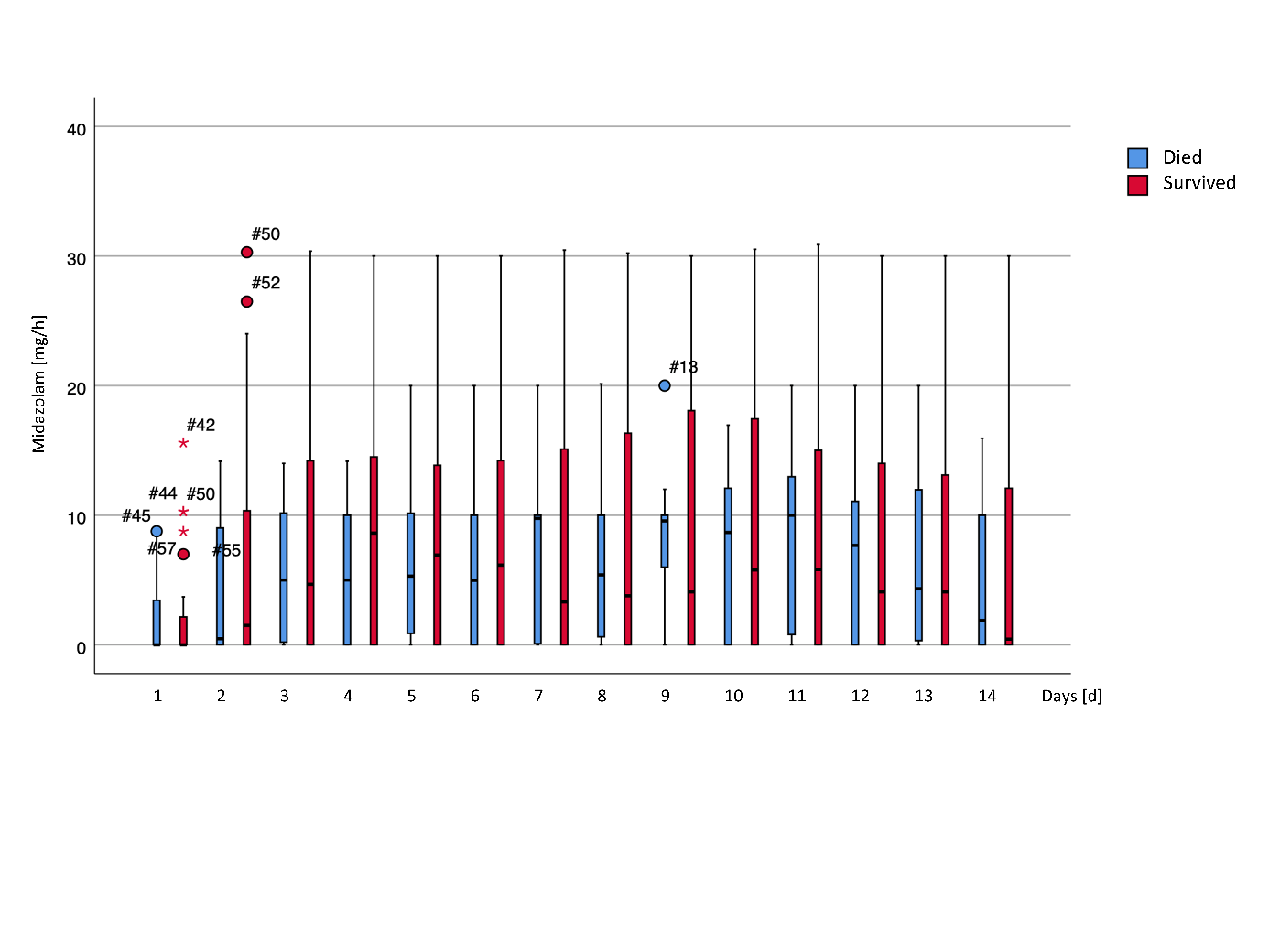


*Daily mean hourly dosage of midazolam. Significant differences between the two groups are marked with an asterisk in the legend of the x-axis.*


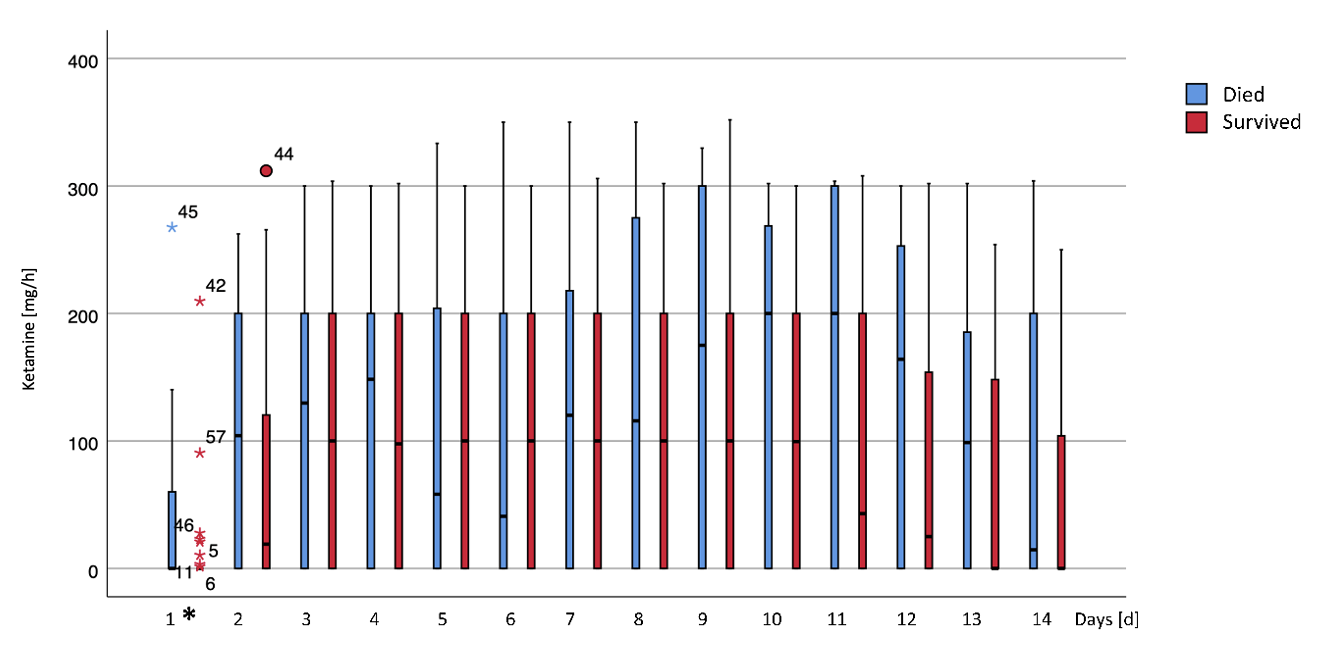


*Daily mean hourly dosage of ketamine. Significant differences between the two groups are marked with an asterisk in the legend of the x-axis.*


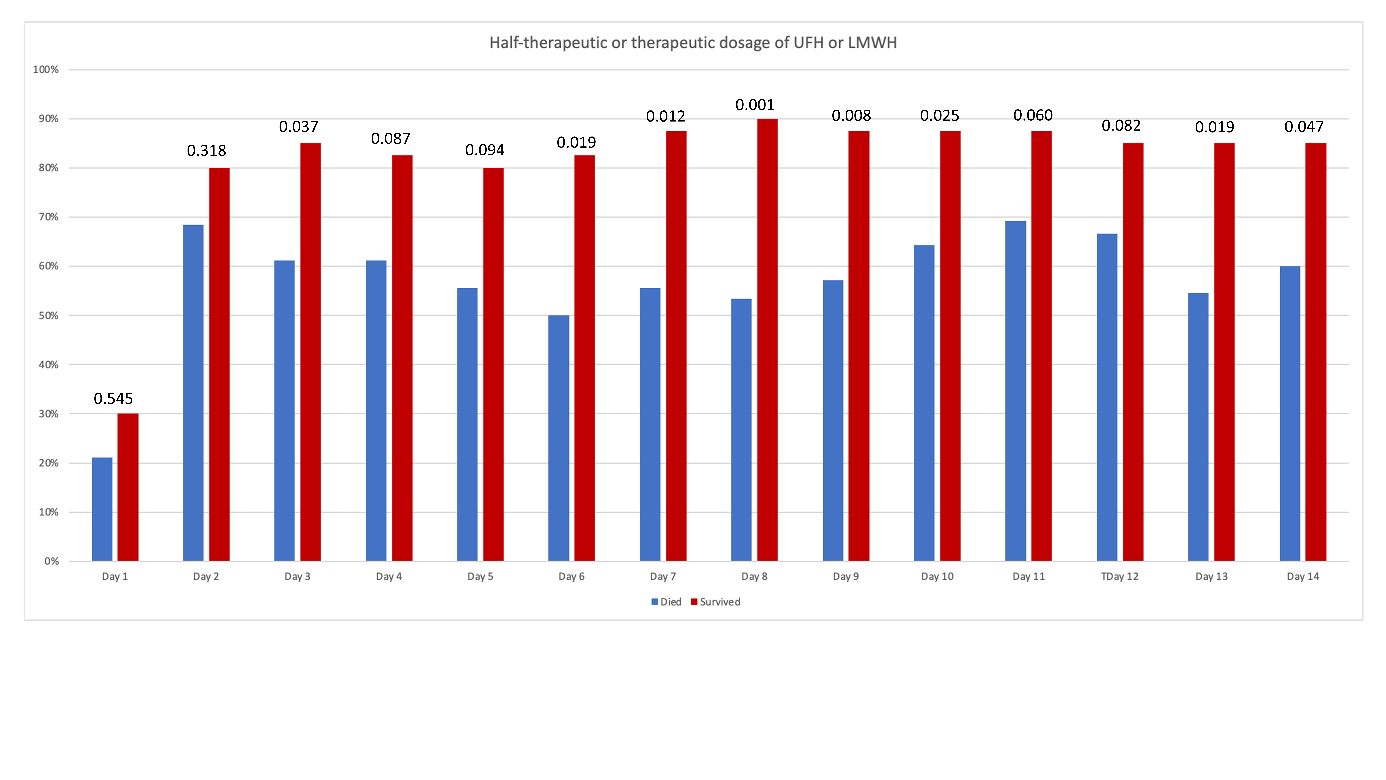


*Frequencies of more than prophylactical doses of unfractionated heparin (UFH) or low molecular weight heparin (LMWH) during the observation period; p-values for comparison between the two groups are stated above the bars.*


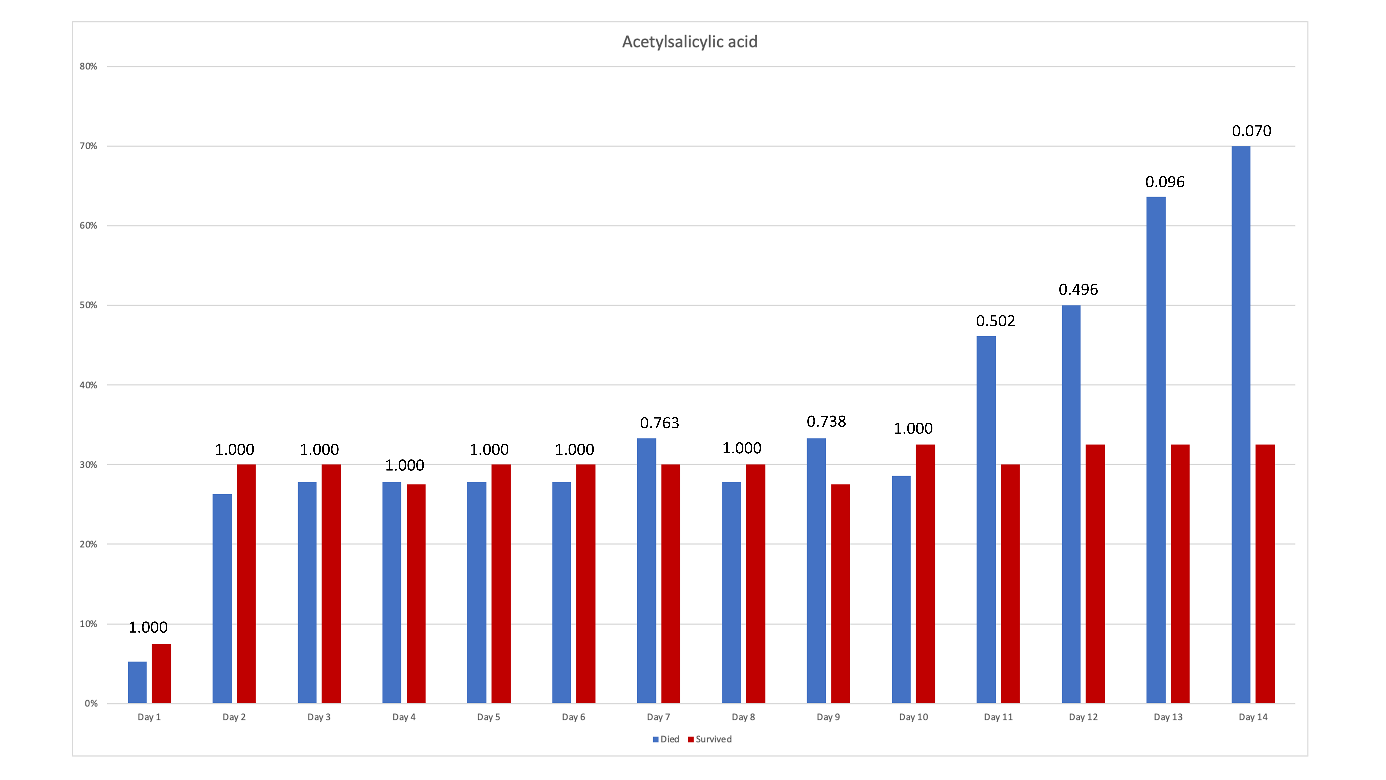


*Frequencies of administration of acetylsalicylic acid during the observation period; p-values for comparison between the two groups are stated above the bars.*
