# Supplementary material for: Lower blood pH as a strong prognostic factor for fatal outcomes in critically ill COVID-19 patients at an intensive care unit: A multivariable analysis
Source: PLoS One. 2021 Sep 29;16(9):e0258018. doi: 10.1371/journal.pone.0258018 (PMC8480873; doi:10.1371/journal.pone.0258018)
Supplement: S5 Fig — (DOCX) [file pone.0258018.s013.docx]

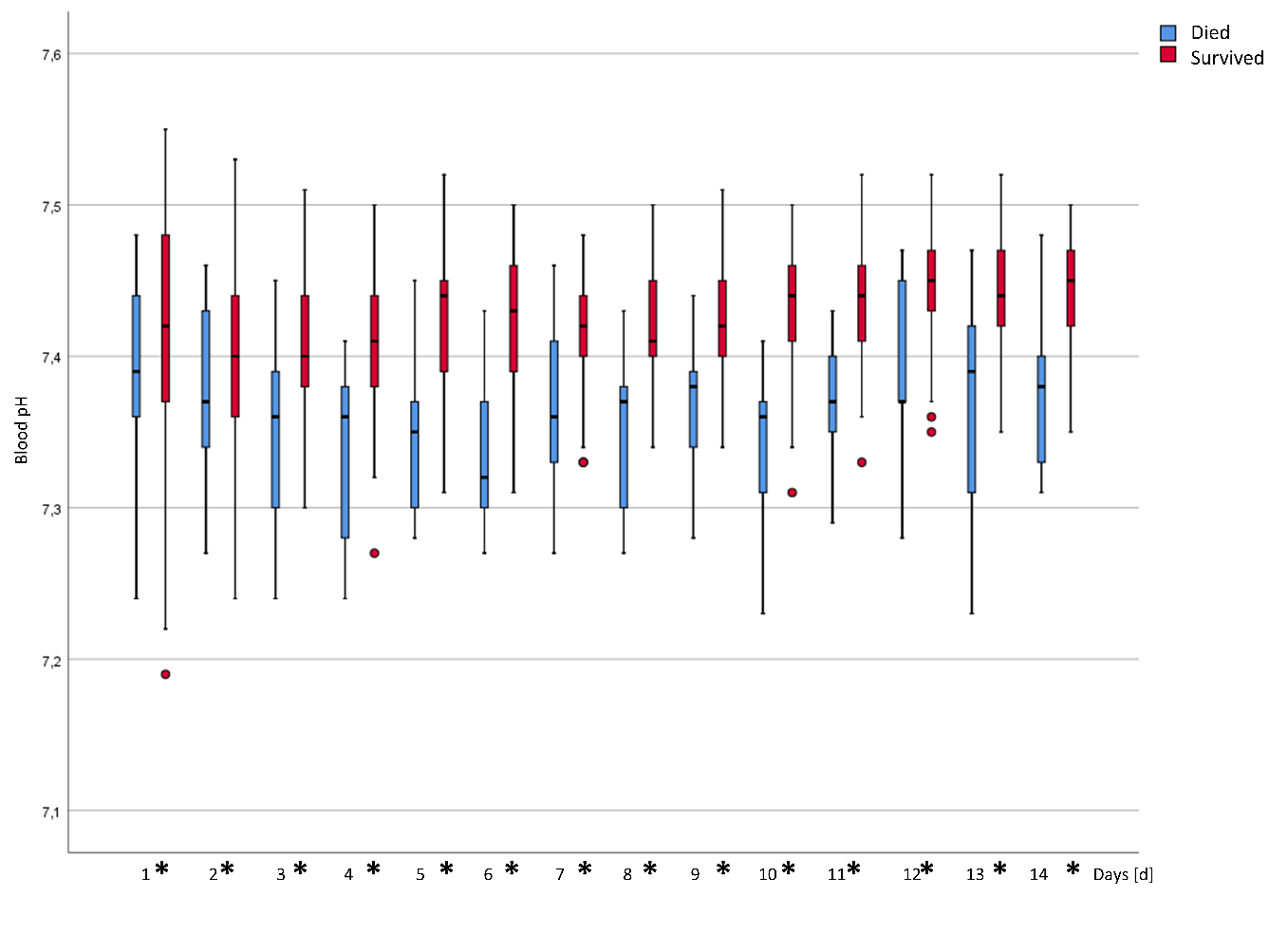


*Daily mean blood pH values. Significant differences between the two groups are marked with an asterisk in the legend of the x-axis.*


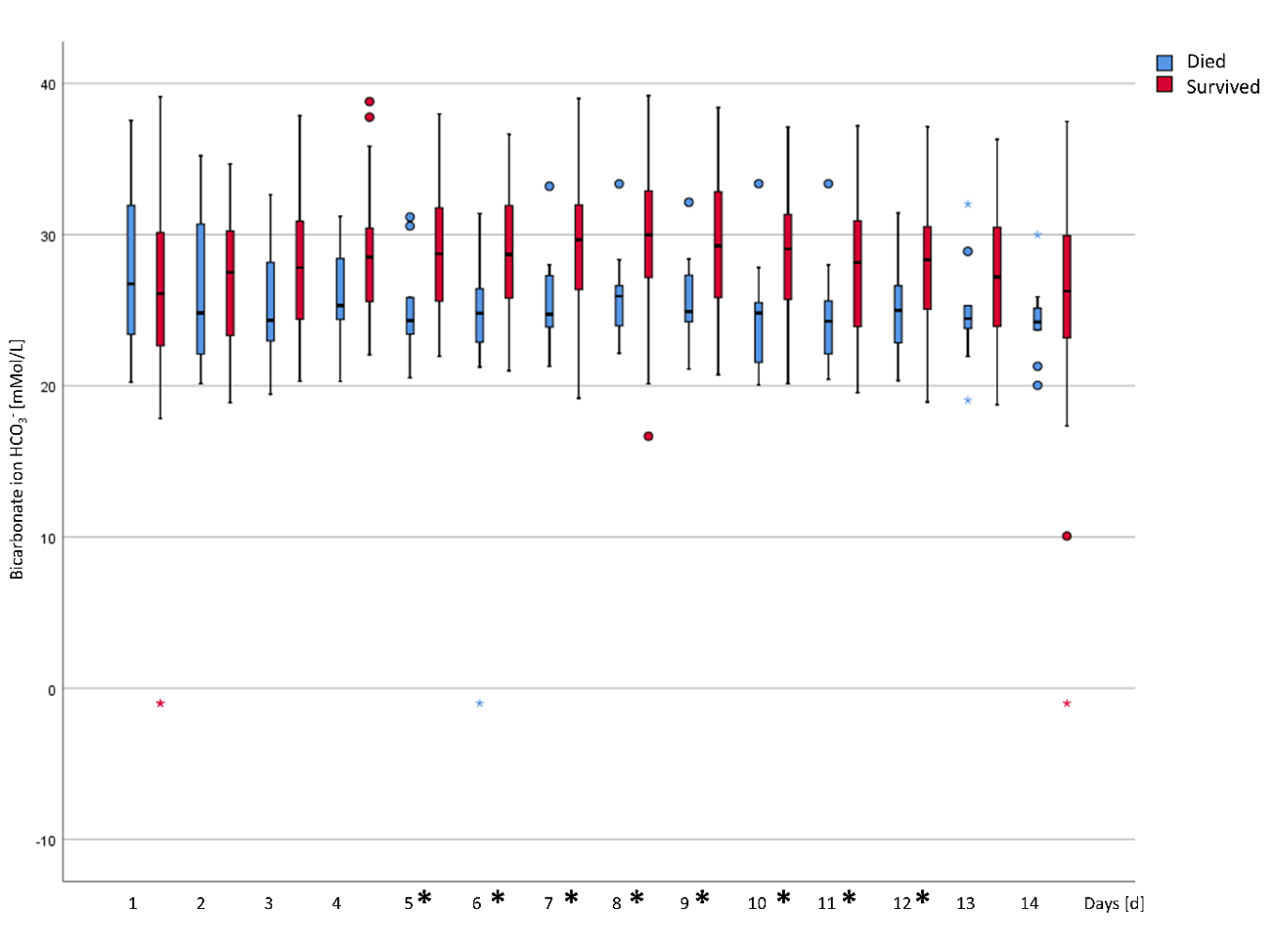


*Daily mean standard bicarbonate values. Significant differences between the two groups are marked with an asterisk in the legend of the x-axis.*


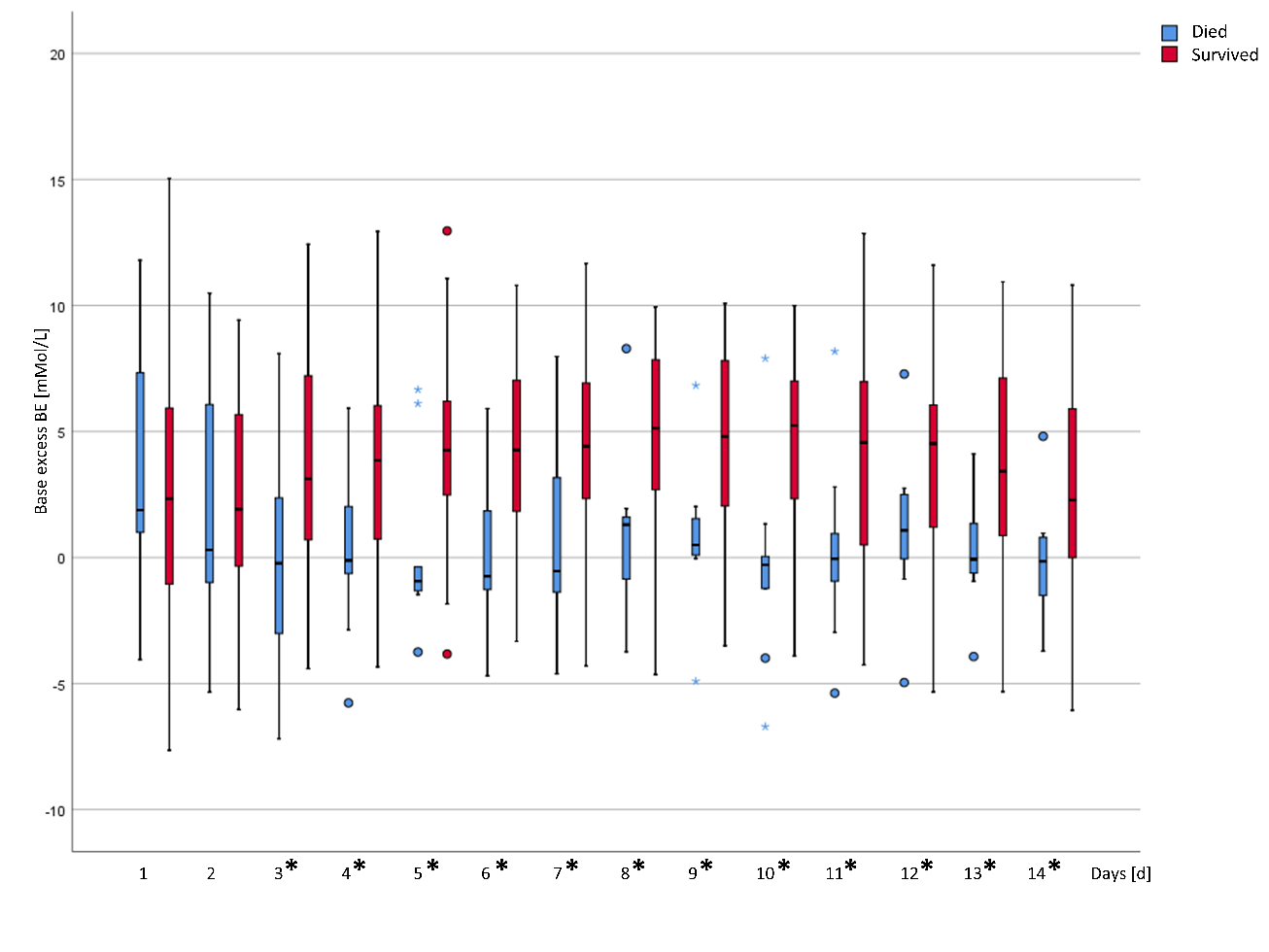


*Daily mean base excess (BE) values. Significant differences between the two groups are marked with an asterisk in the legend of the x-axis*


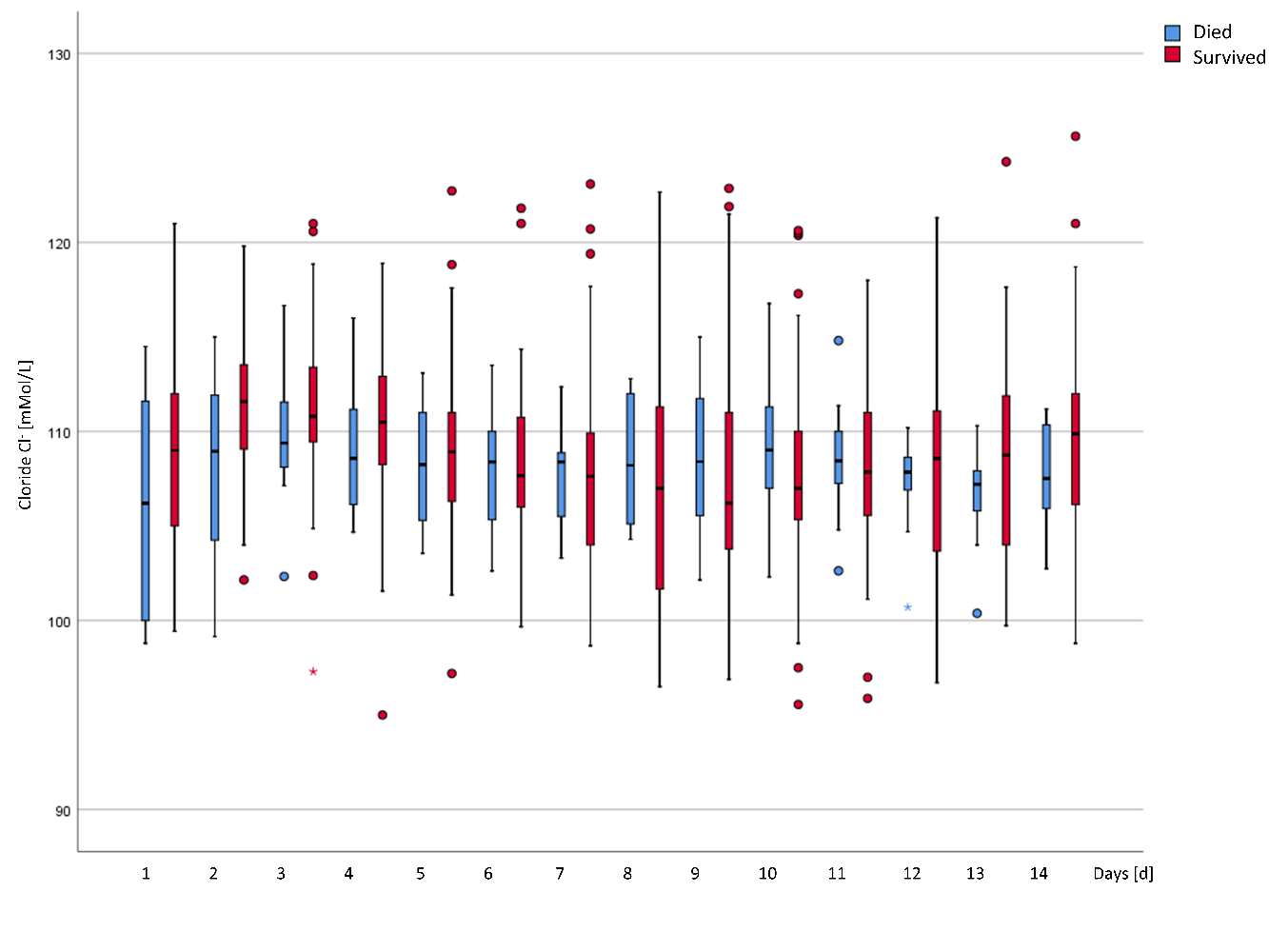


*Daily mean blood chloride values. Significant differences between the two groups are marked with an asterisk in the legend of the x-axis.*


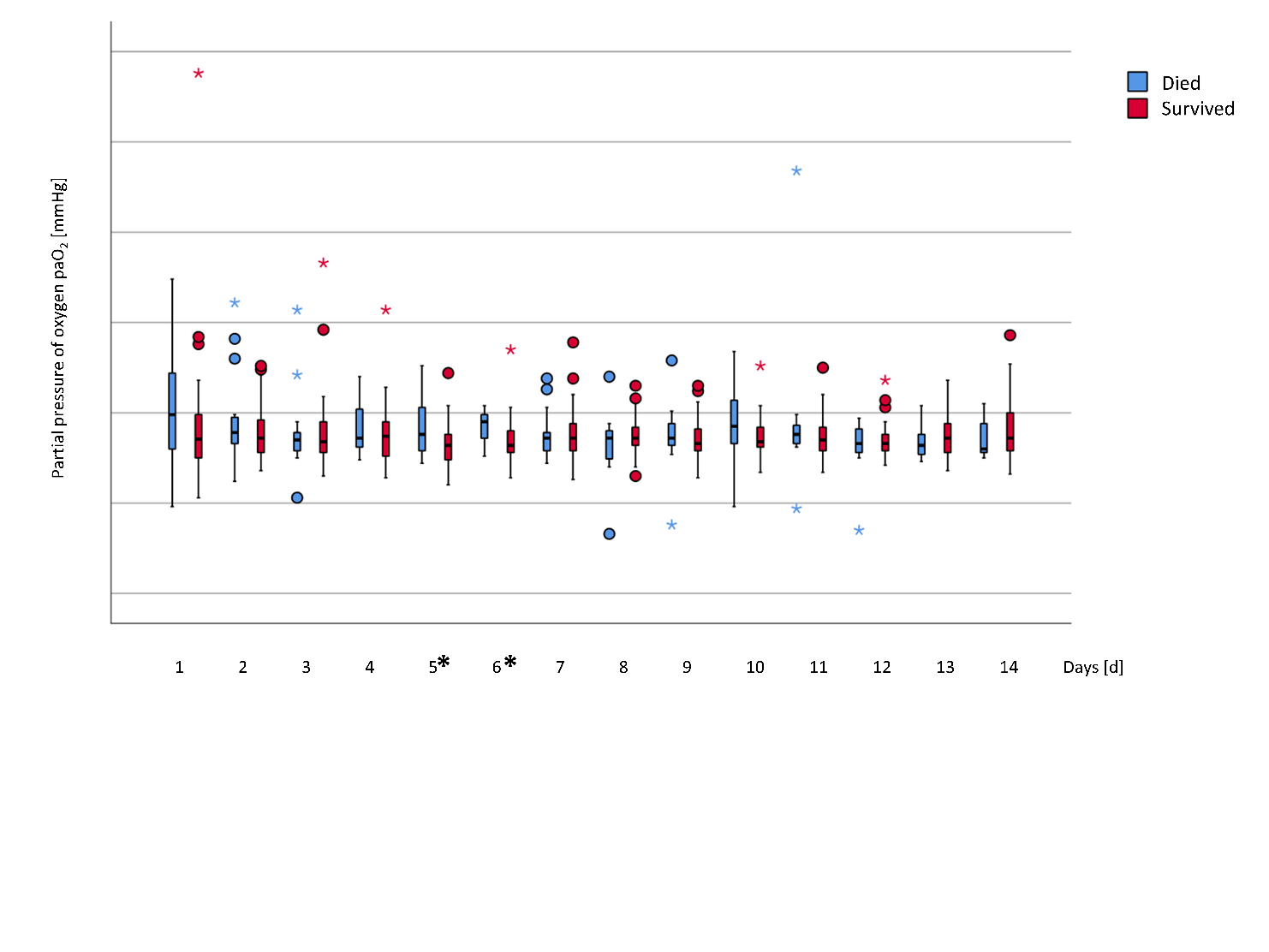


*Daily mean values for arterial partial pressure of oxygen (paO_2_). Significant differences between the two groups are marked with an asterisk in the legend of the x-axis.*


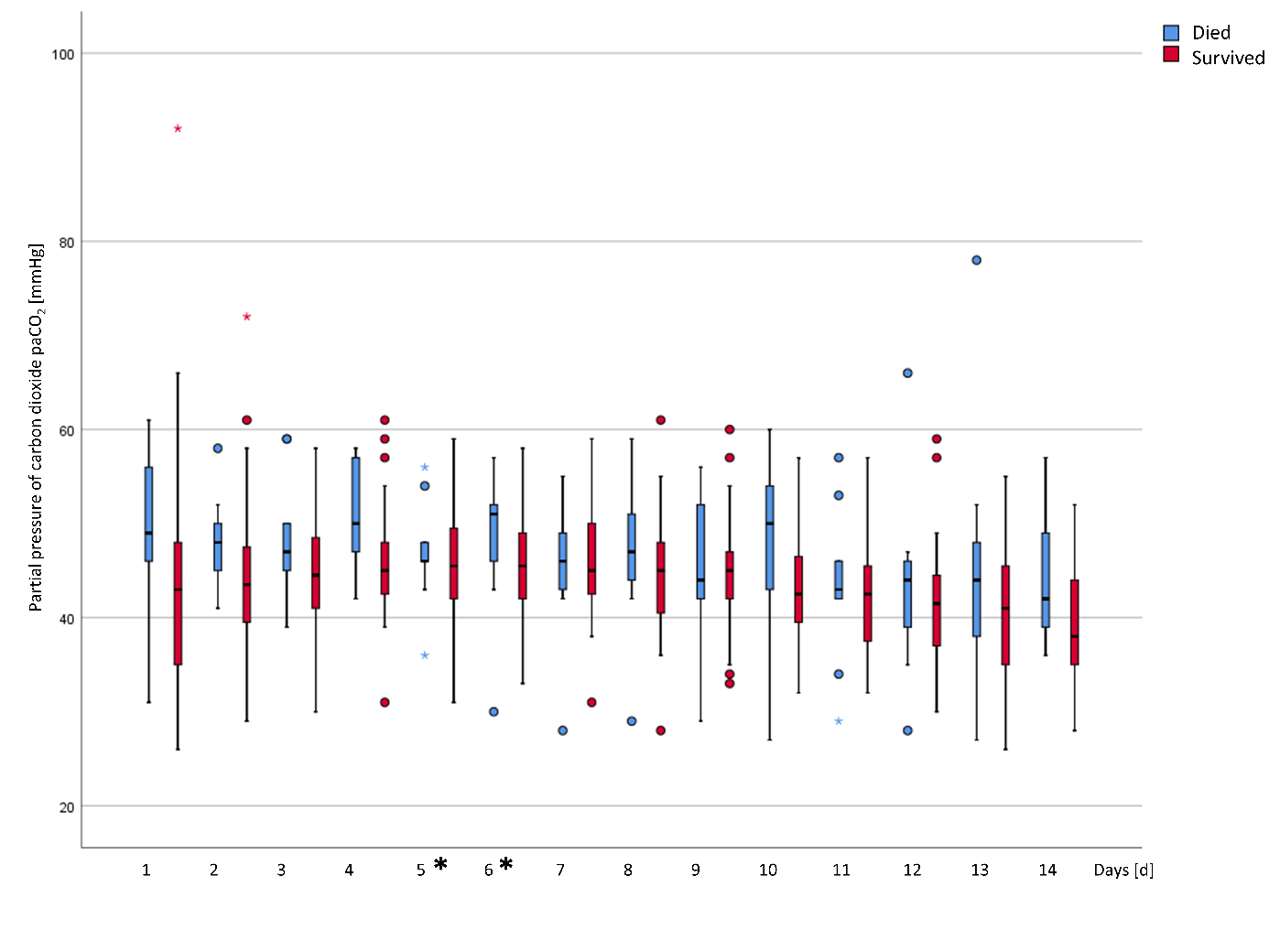


*Daily mean values for arterial partial pressure of carbon dioxide (paCO_2_). Significant differences between the two groups are marked with an asterisk in the legend of the x-axis.*


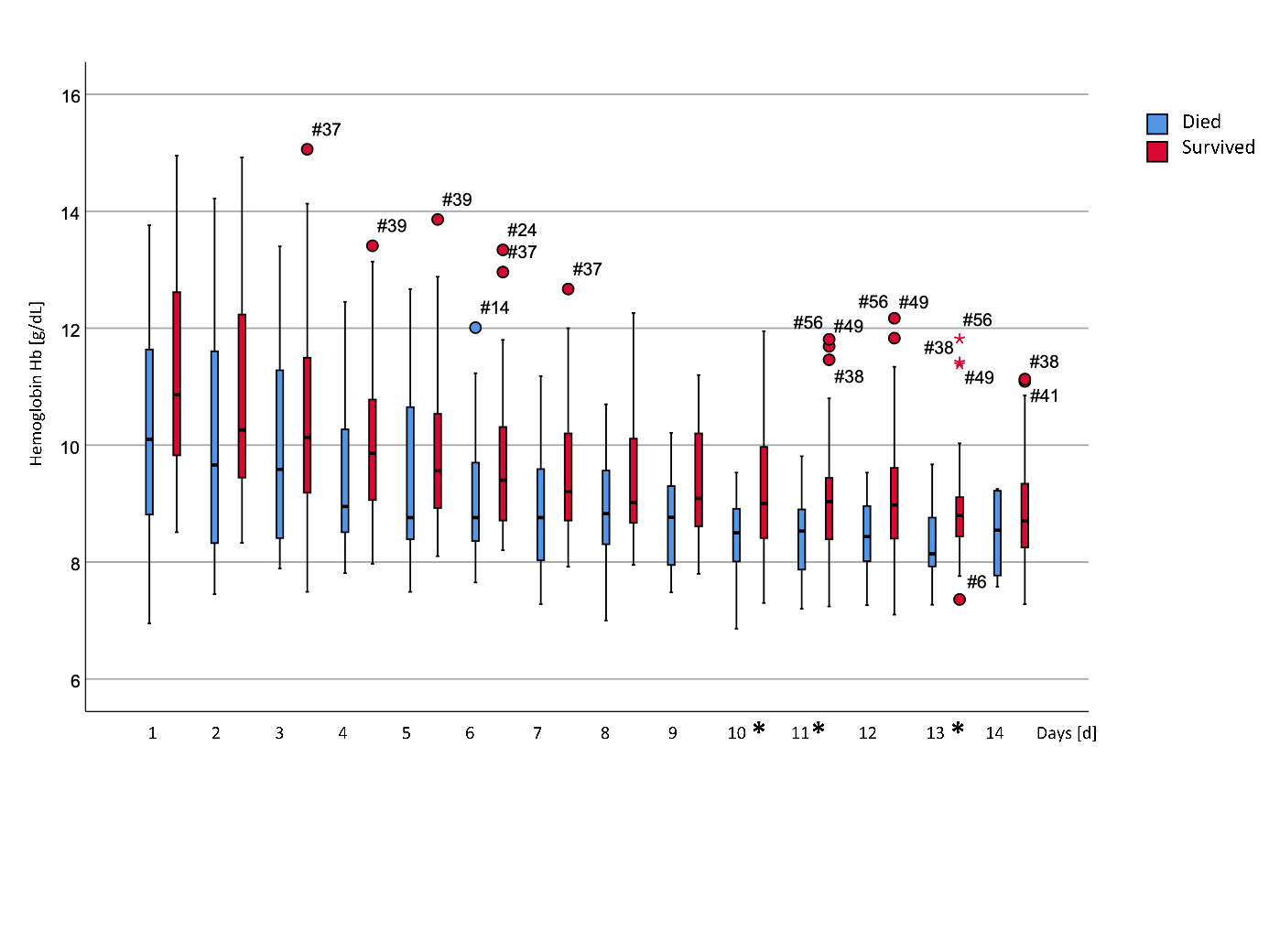


*Daily mean values for hemoglobin (Hb). Significant differences between the two groups are marked with an asterisk in the legend of the x-axis.*


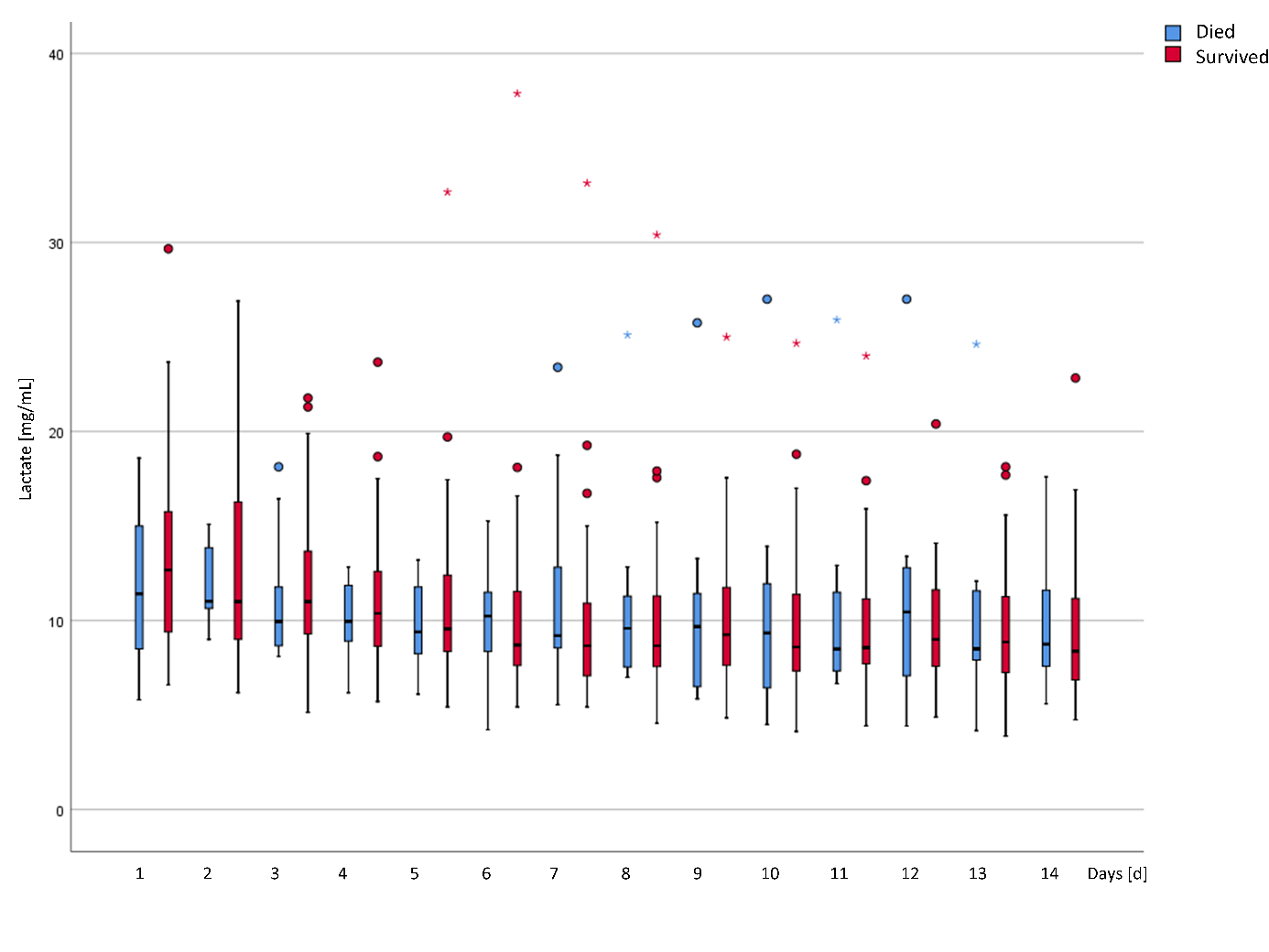


*Daily mean values for blood lactate. Significant differences between the two groups are marked with an asterisk in the legend of the x-axis.*


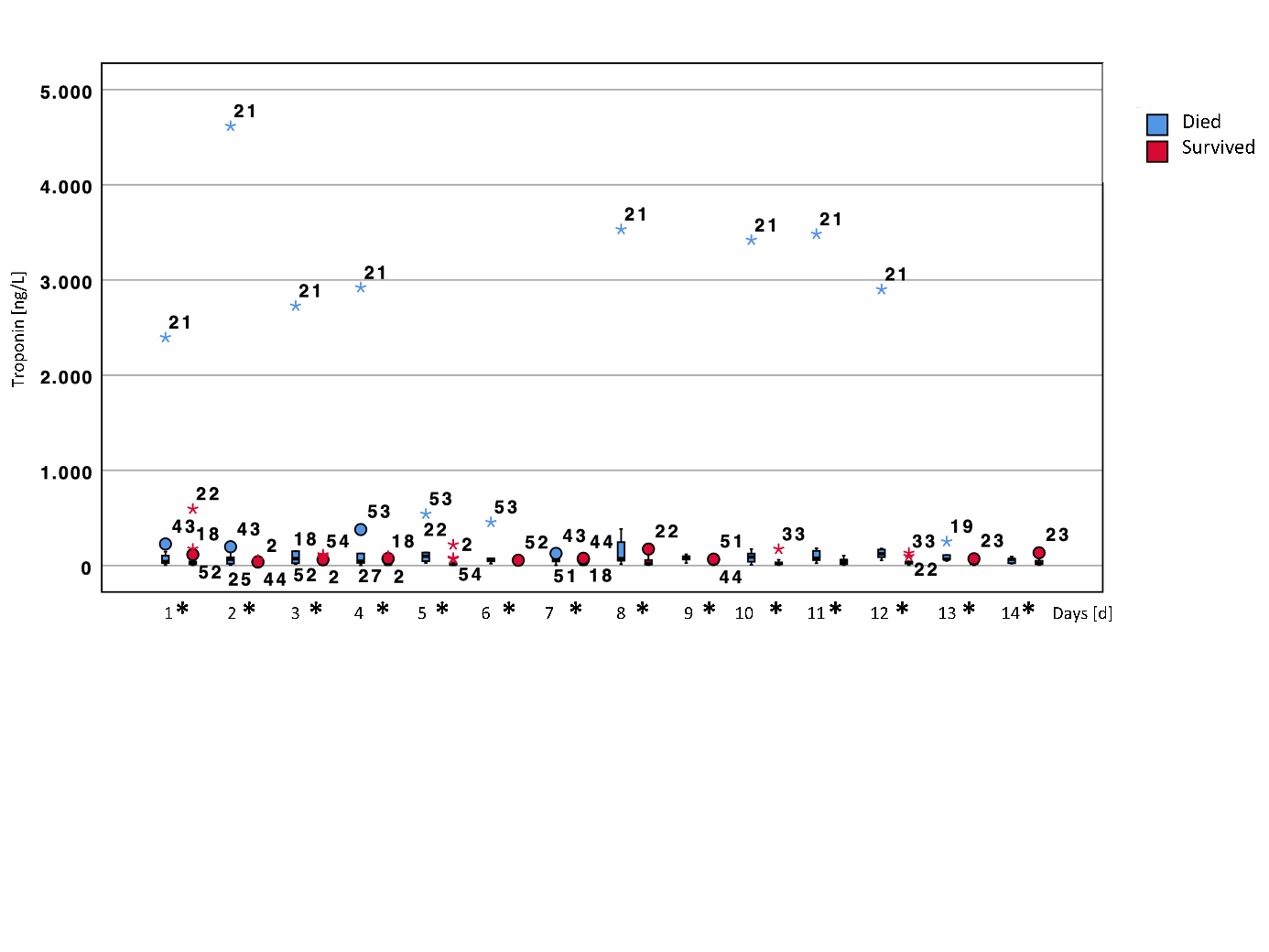


*Daily values for troponin T. Significant differences between the two groups are marked with an asterisk in the legend of the x-axis.*


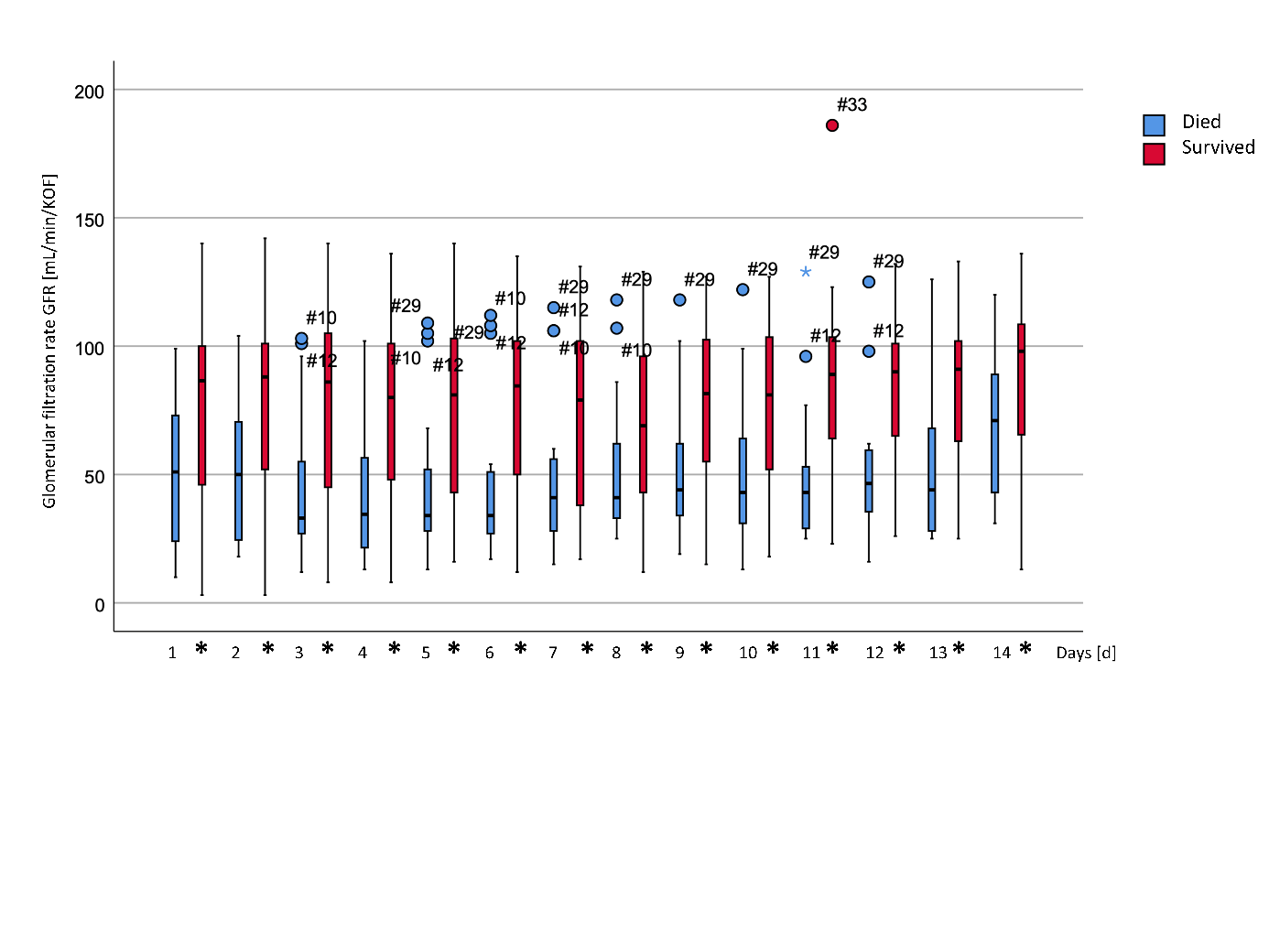


*Daily values for the troponin estimated glomerular filtration rate (eGFR). Significant differences between the two groups are marked with an asterisk in the legend of the x-axis.*


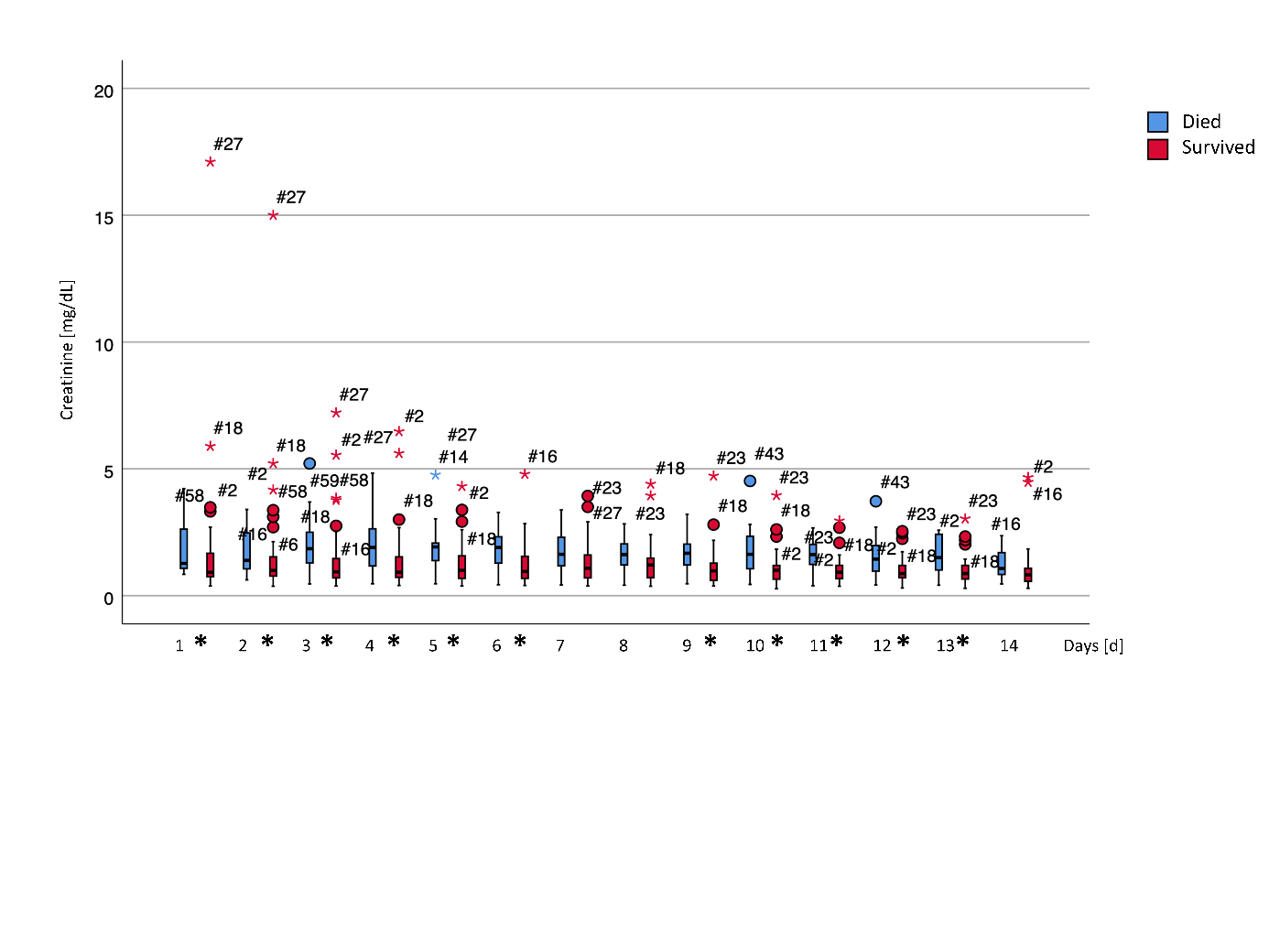


*Daily values for blood creatinine (Crea). Significant differences between the two groups are marked with an asterisk in the legend of the x-axis.*


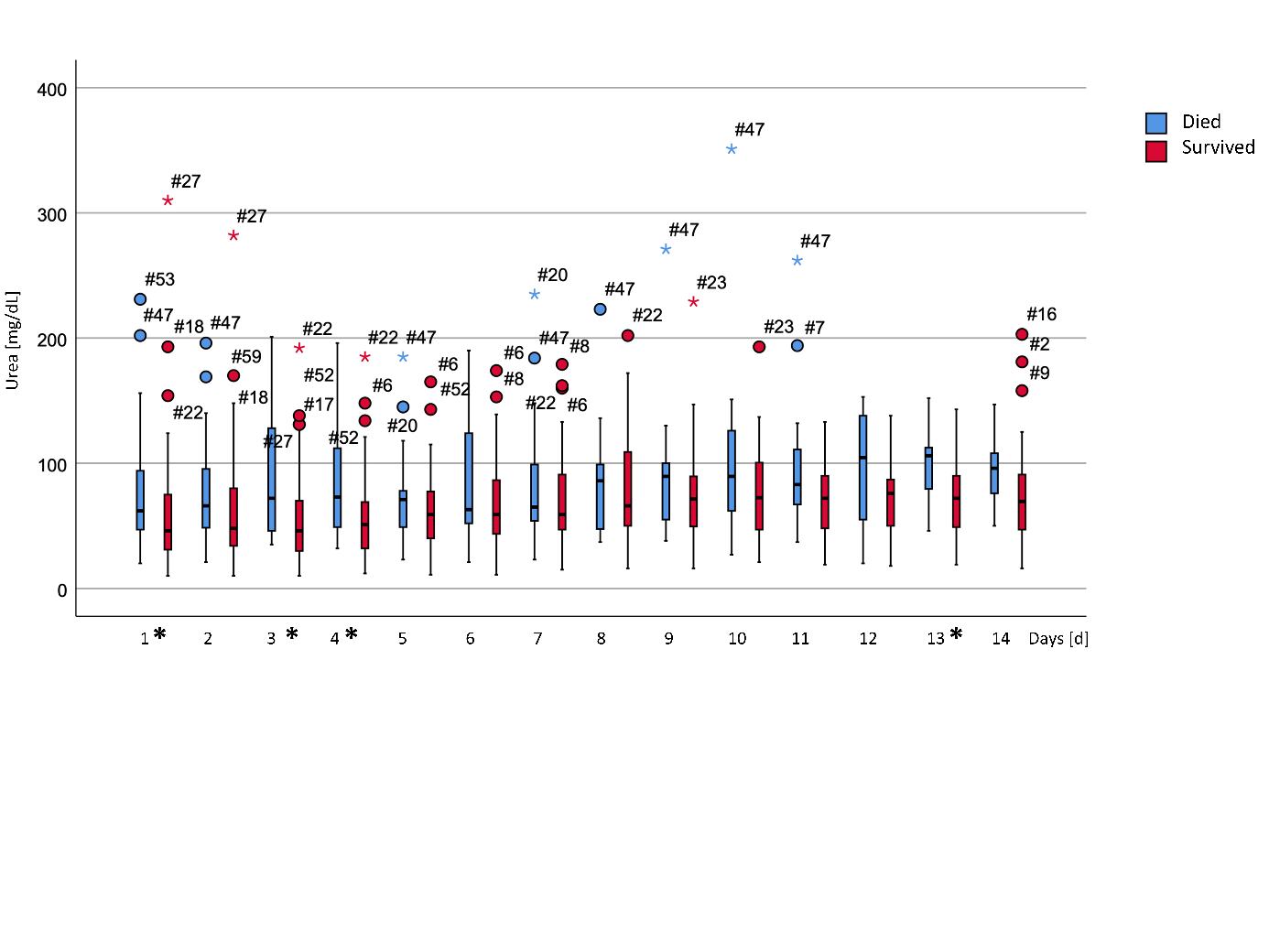


*Daily values for urea. Significant differences between the two groups are marked with an asterisk in the legend of the x-axis.*


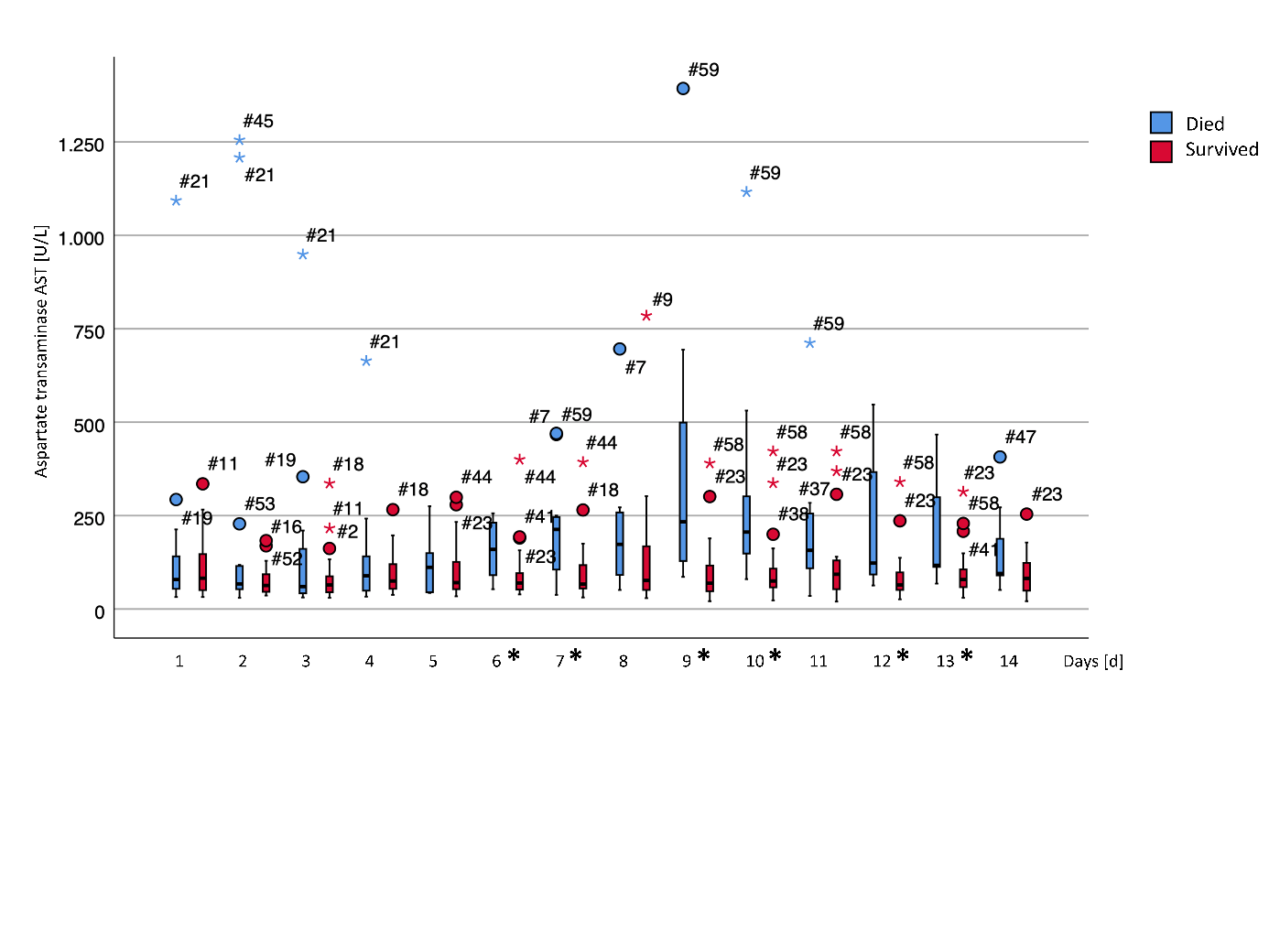


*Daily values for aspartate transaminase (AST). Significant differences between the two groups are marked with an asterisk in the legend of the x-axis.*


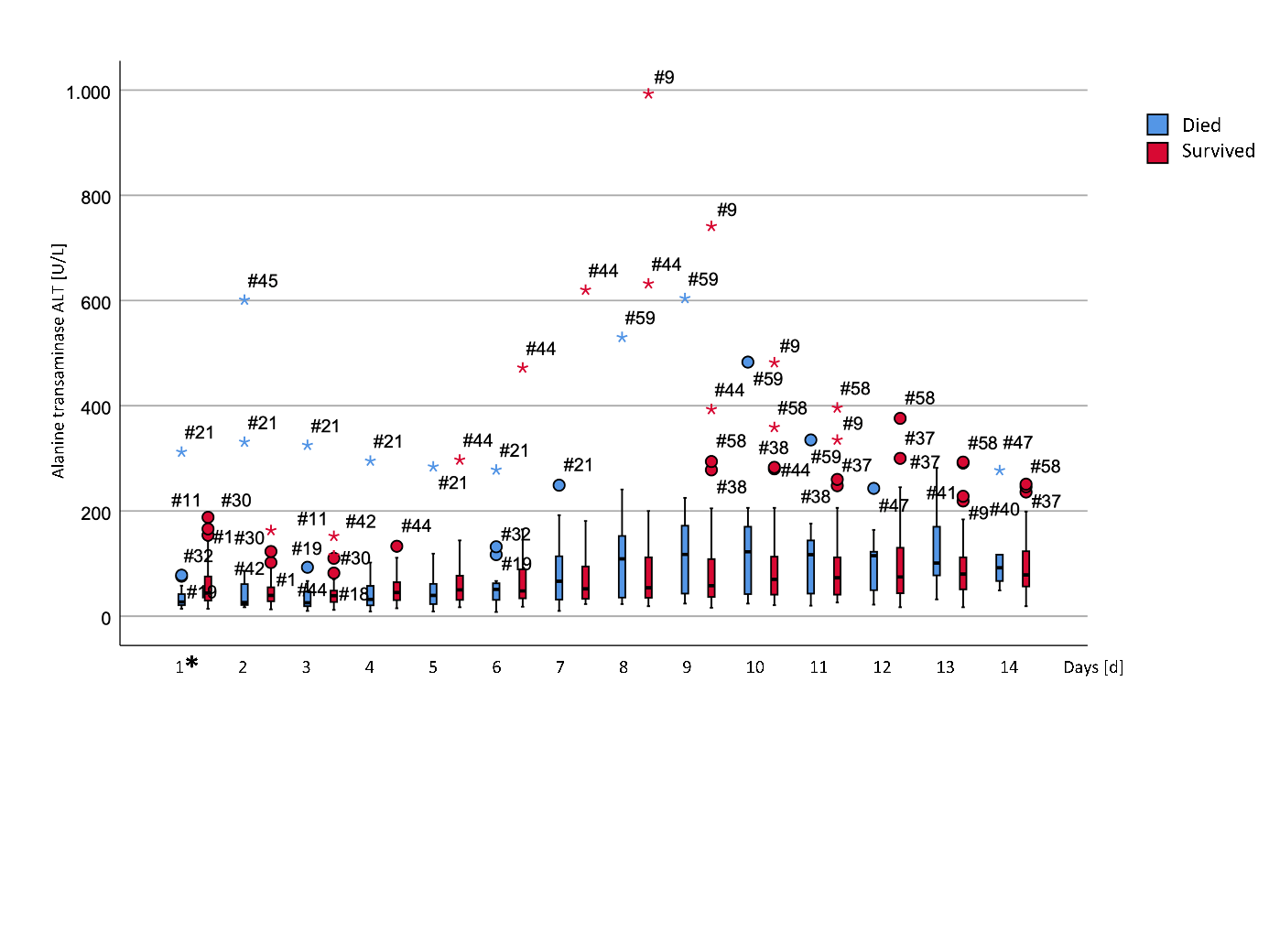


*Daily values for alanine transaminase (ALT). Significant differences between the two groups are marked with an asterisk in the legend of the x-axis.*


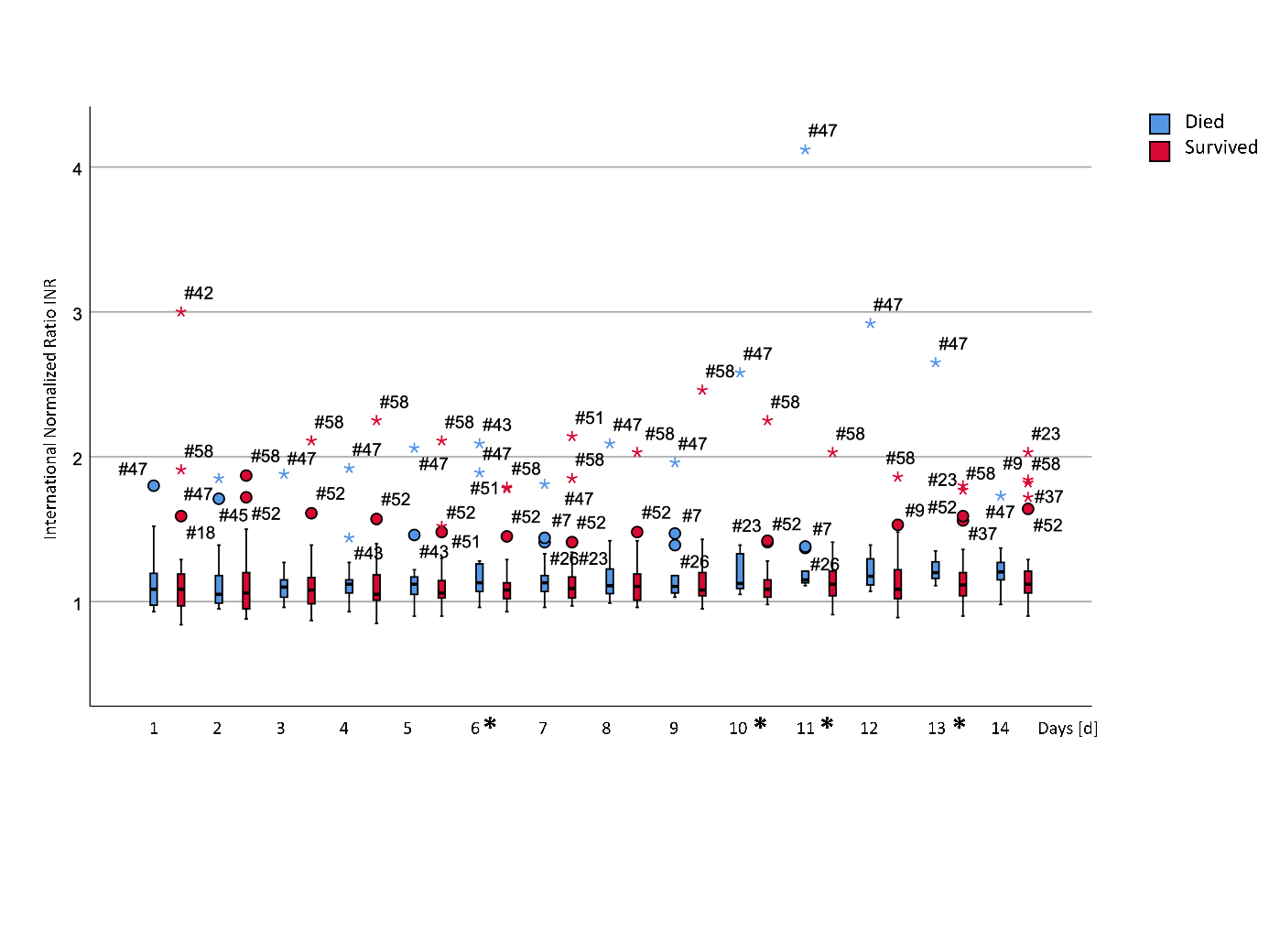


*Daily values for the international normalized ratio (INR). Significant differences between the two groups are marked with an asterisk in the legend of the x-axis.*


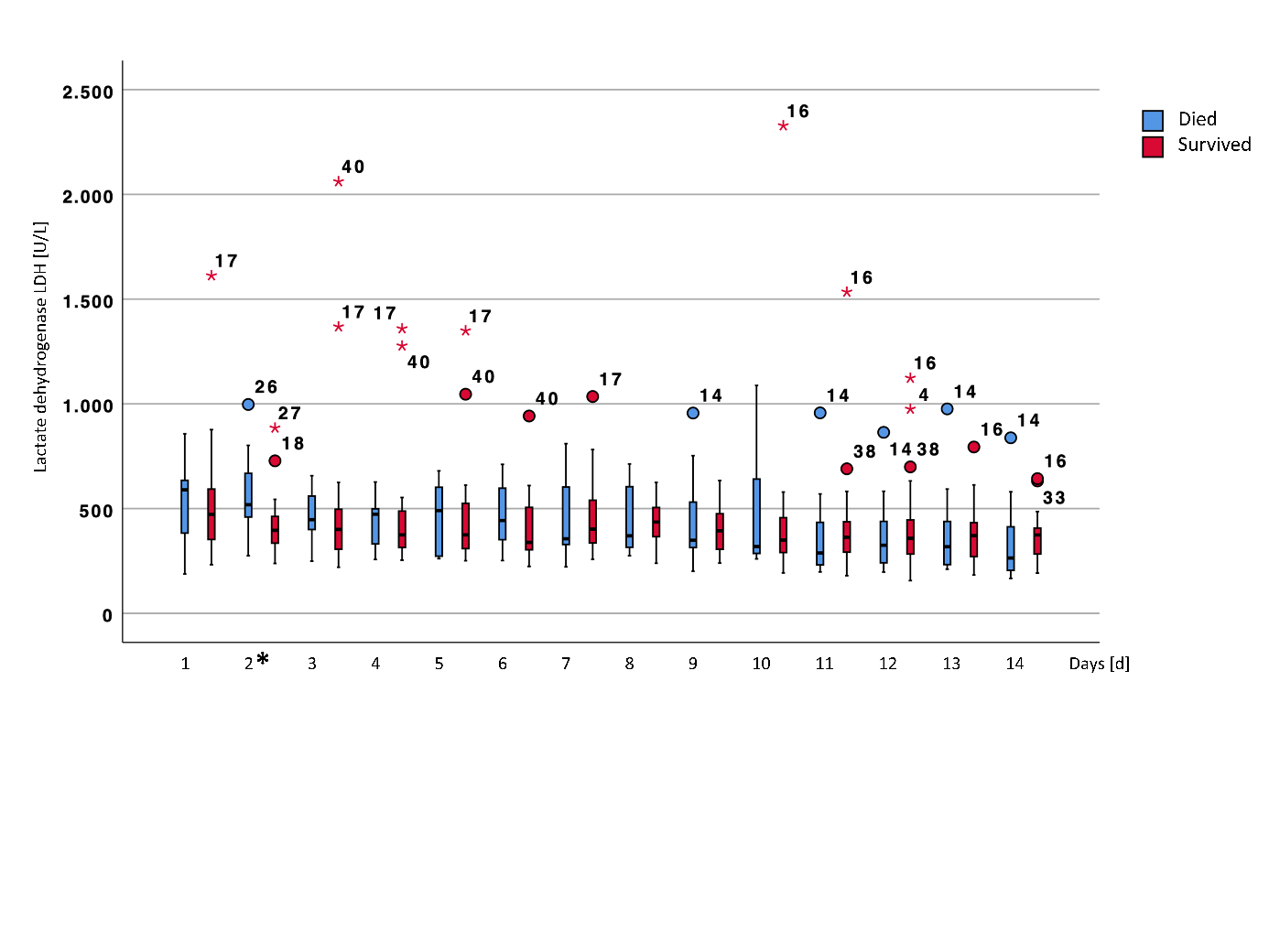


*Daily values for lactate dehydrogenase (LDH). Significant differences between the two groups are marked with an asterisk in the legend of the x-axis.*


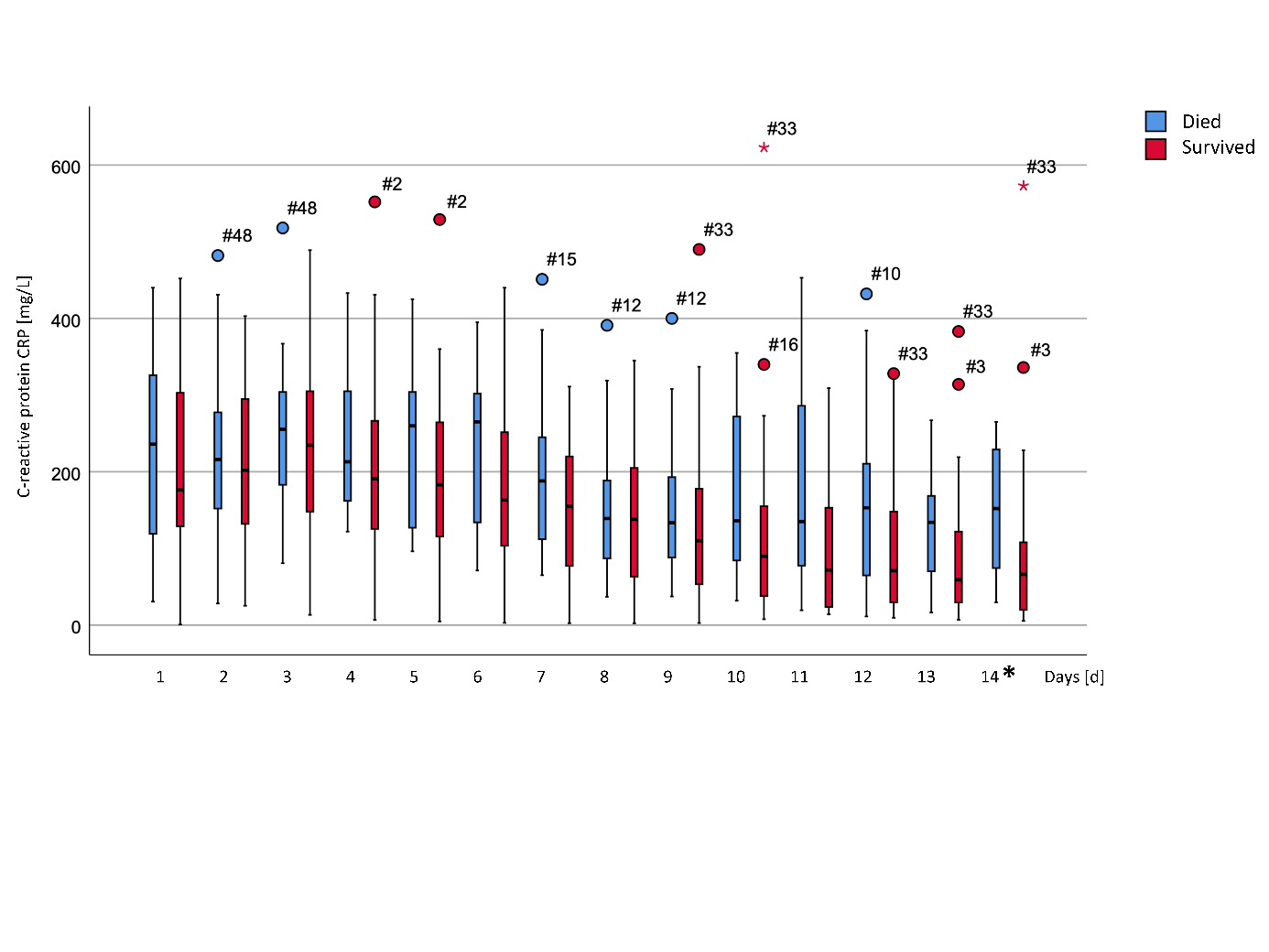


*Daily values for C-reactive protein (CRP). Significant differences between the two groups are marked with an asterisk in the legend of the x-axis.*

**
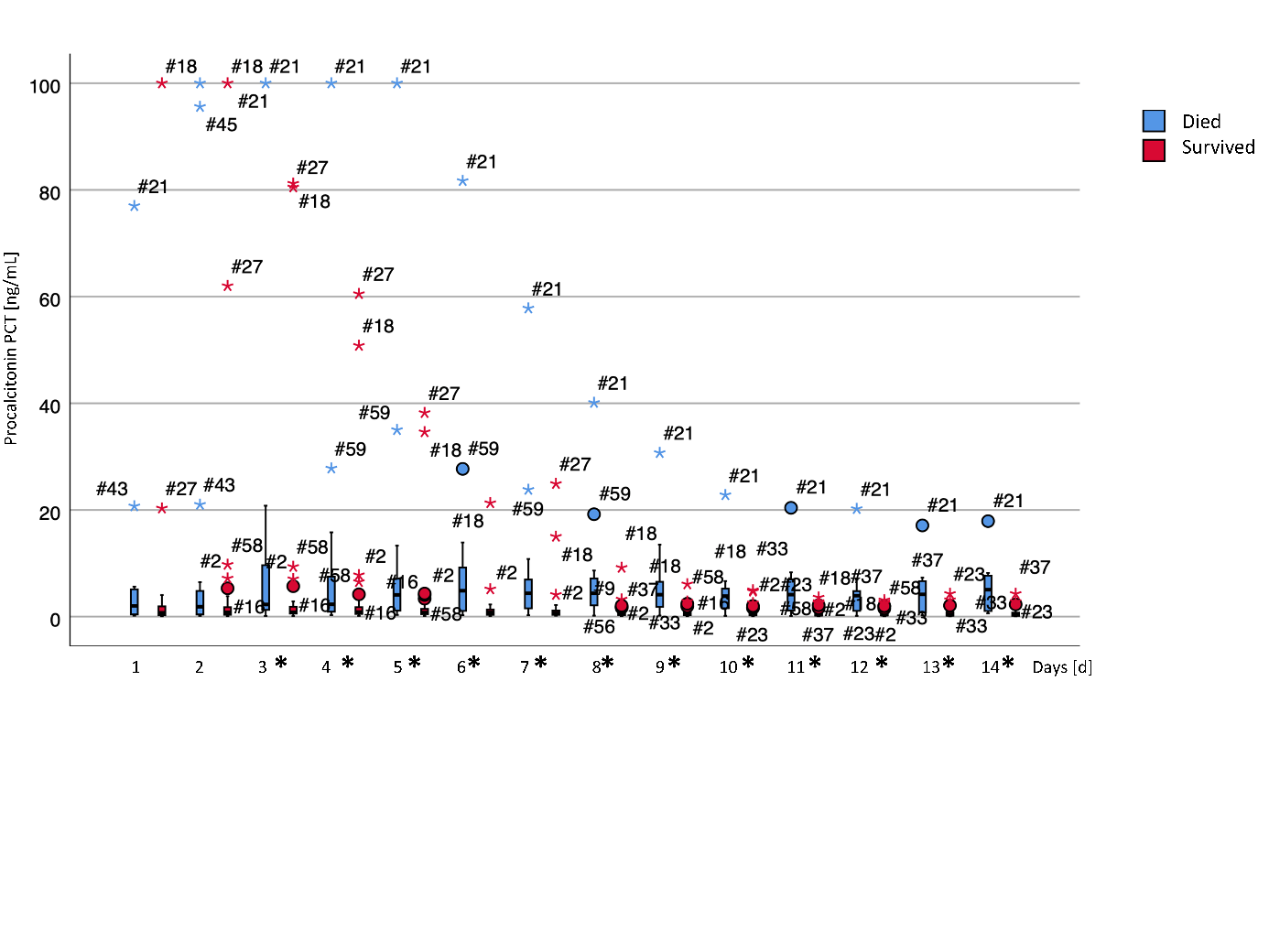
**

*Daily values for procalcitonin (PCT). Significant differences between the two groups are marked with an asterisk in the legend of the x-axis.*


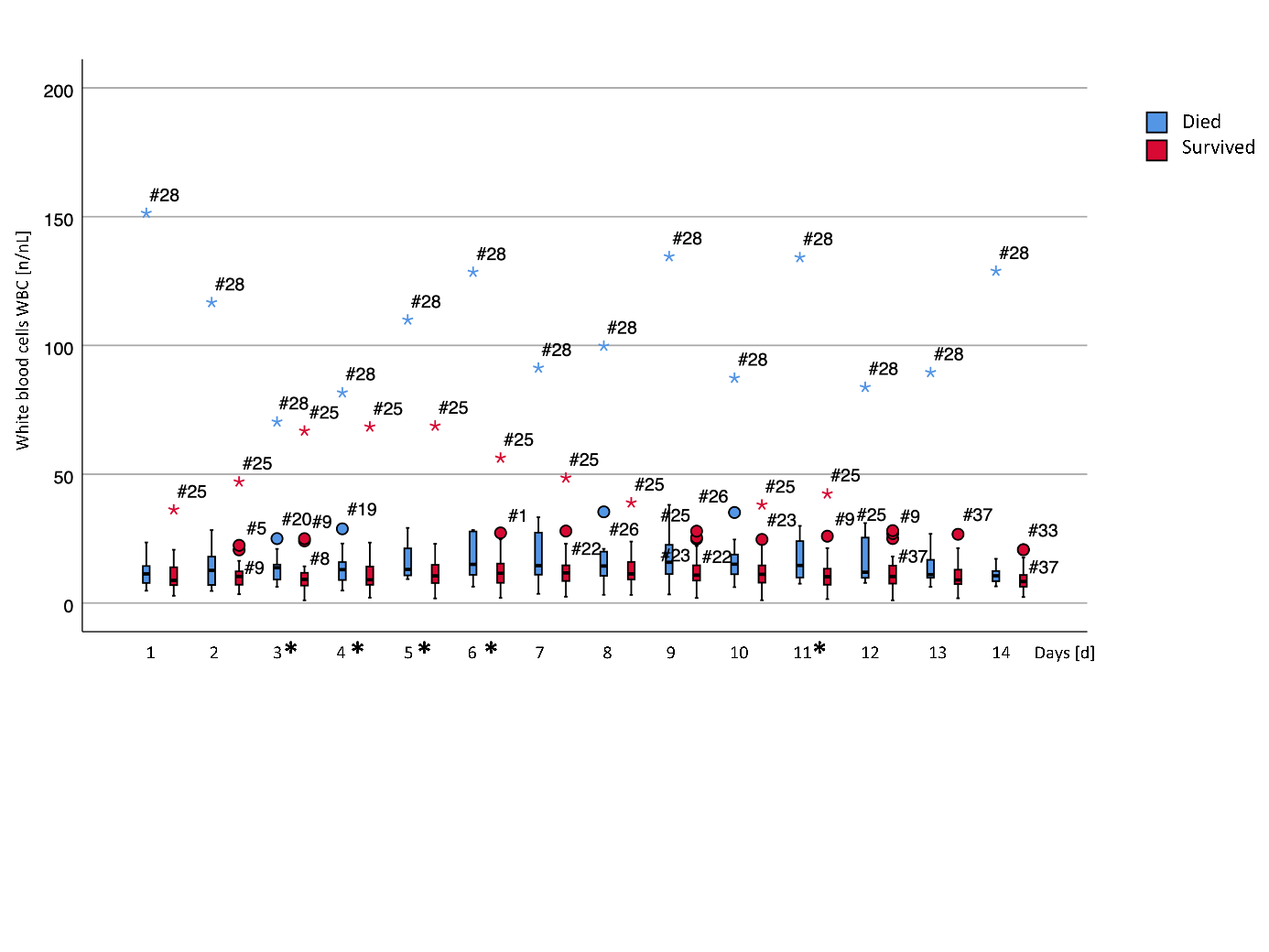


*Daily values for the white blood cell count (WBC). Significant differences between the two groups are marked with an asterisk in the legend of the x-axis.*


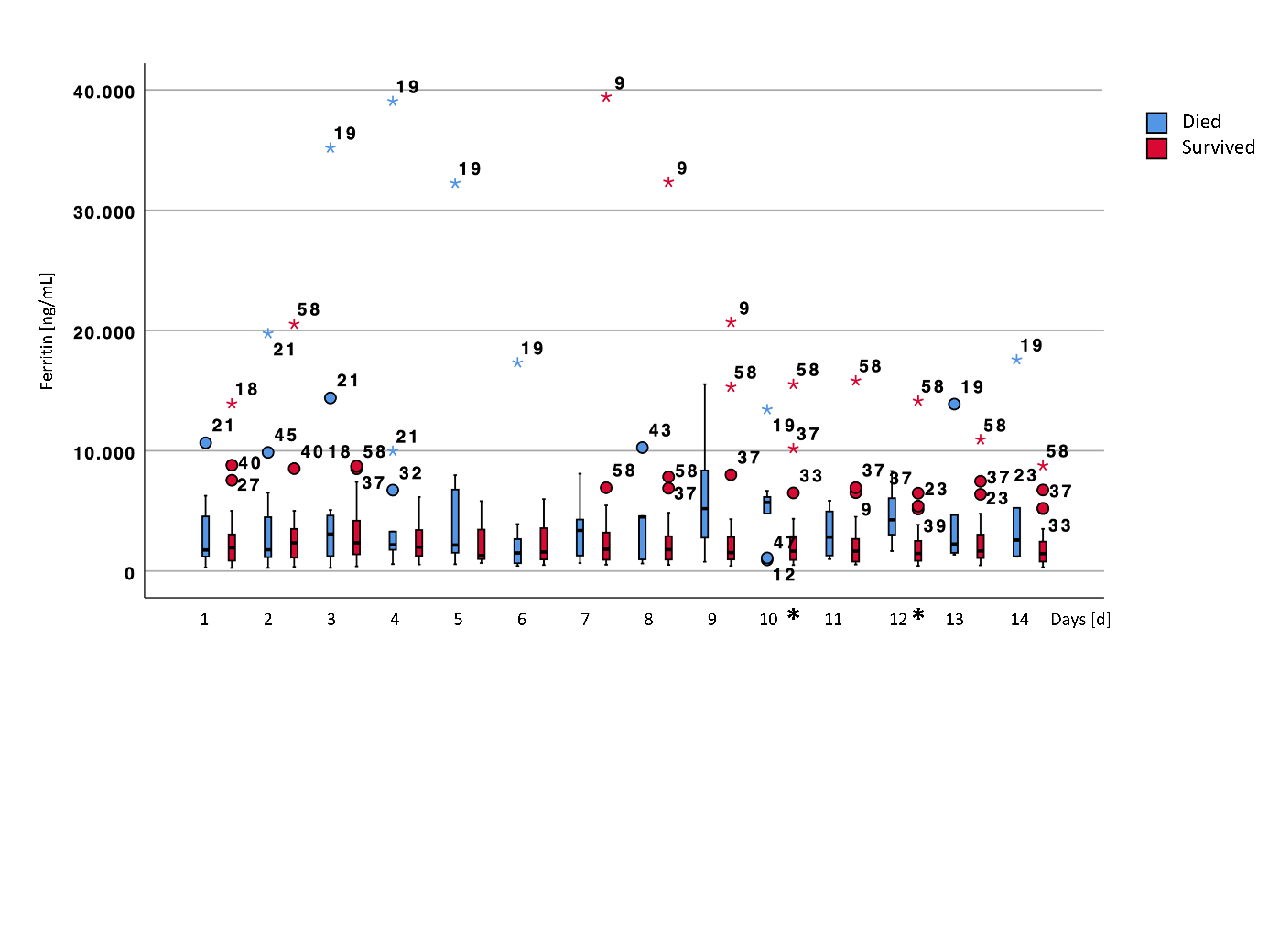


*Daily values for ferritin. Significant differences between the two groups are marked with an asterisk in the legend of the x-axis.*


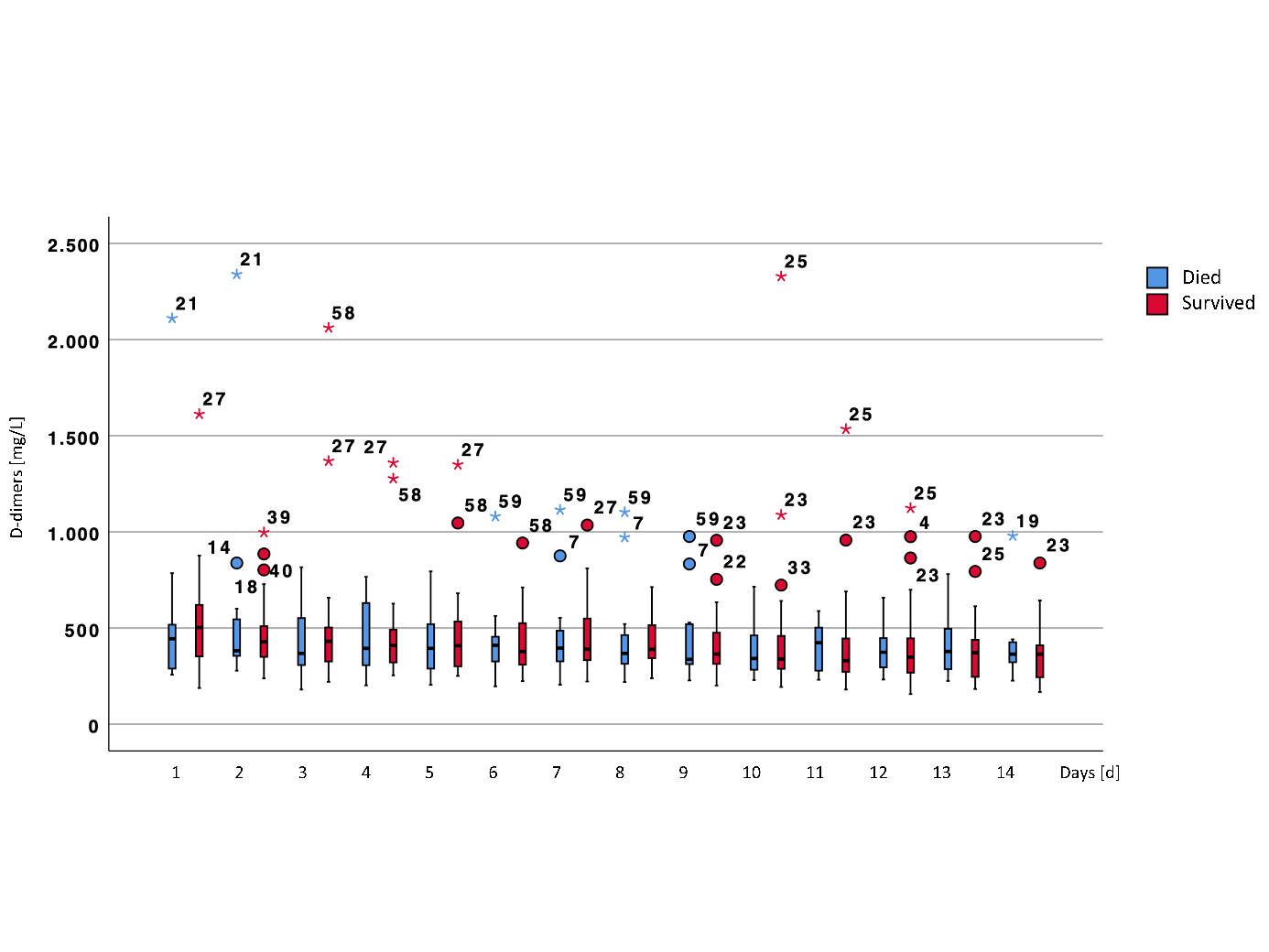


*Daily values for D-dimers. Significant differences between the two groups are marked with an asterisk in the legend of the x-axis.*


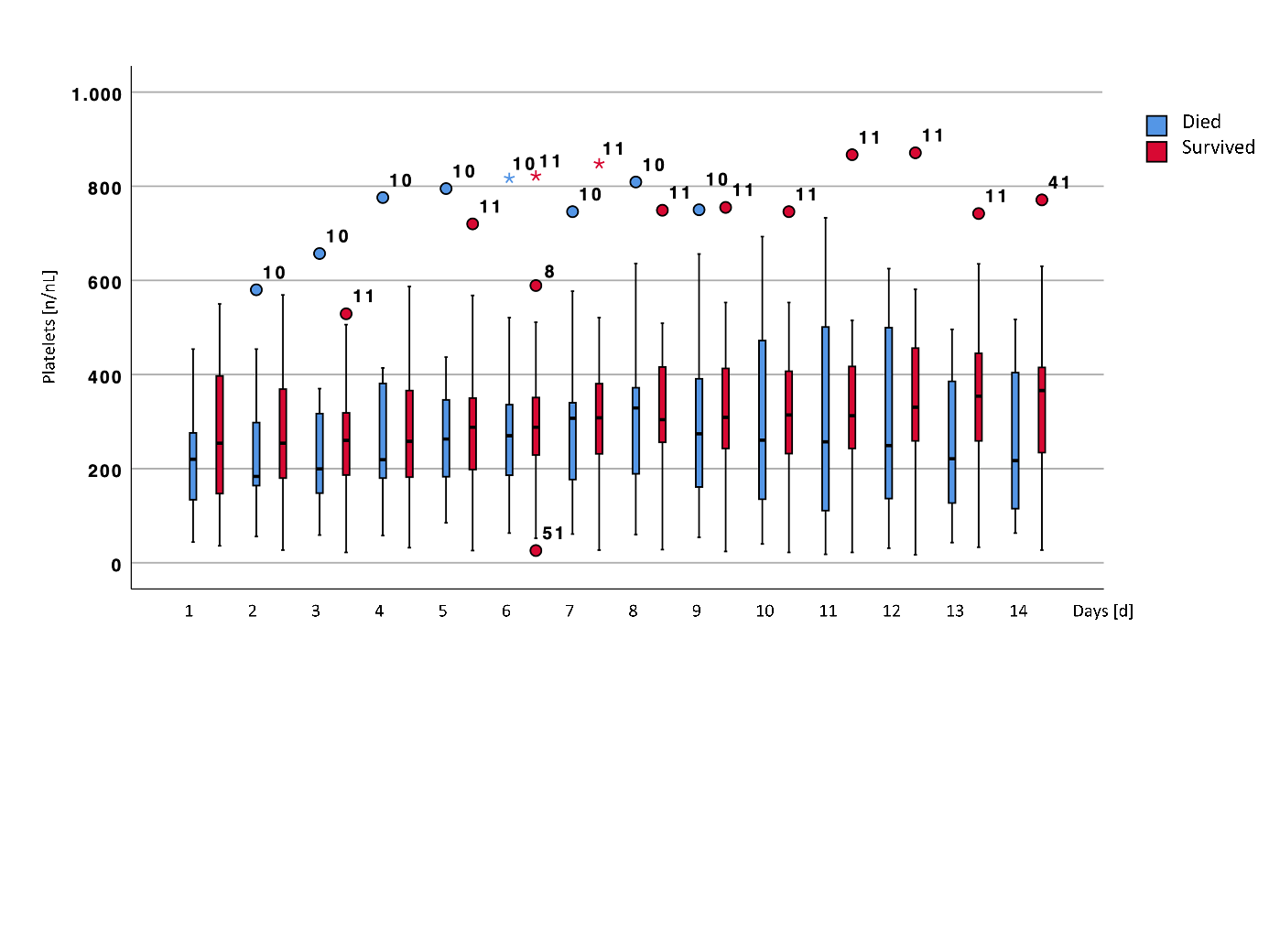


*Daily values for the platelet count. Significant differences between the two groups are marked with an asterisk in the legend of the x-axis.*


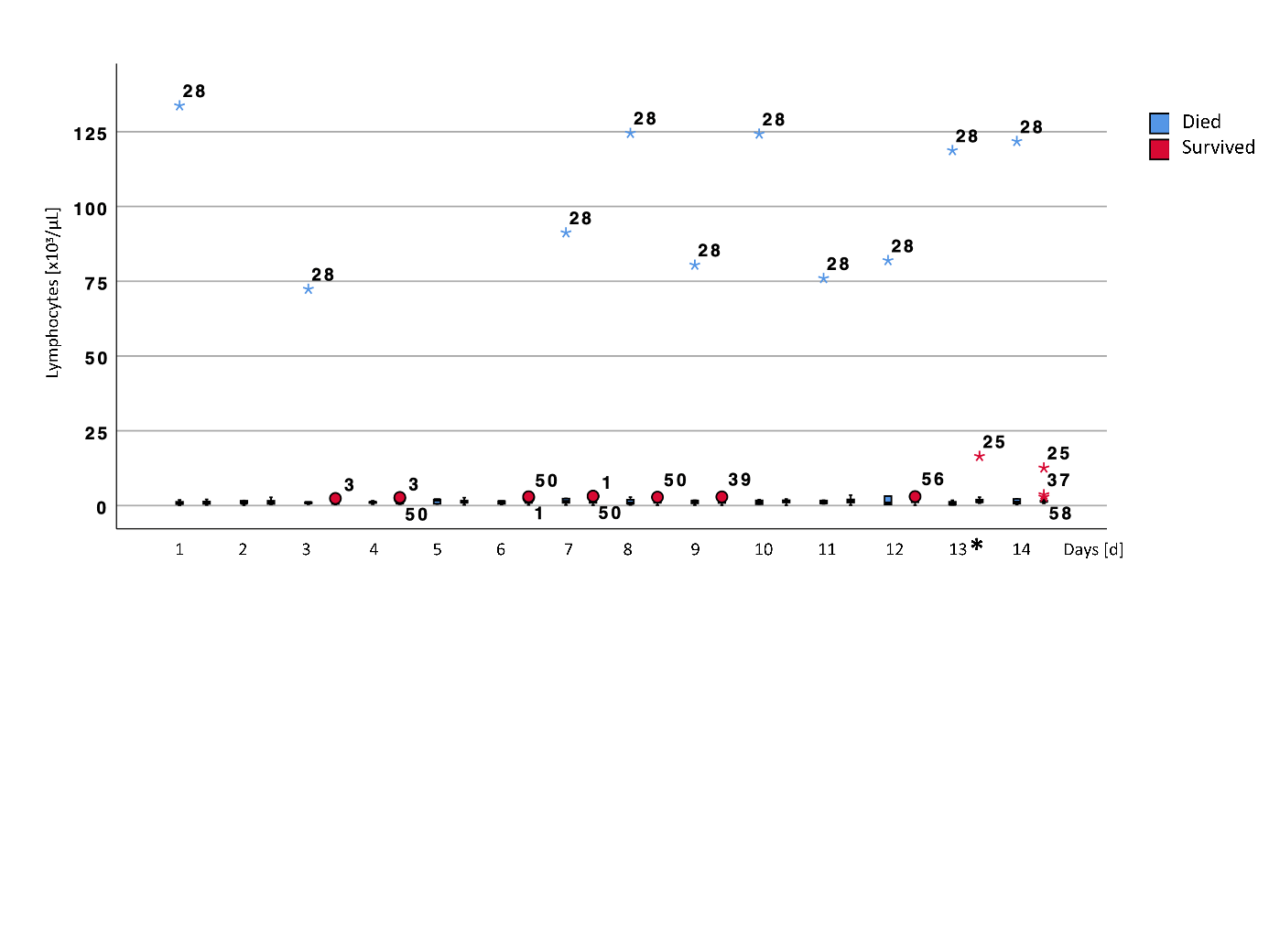


*Daily values for the lymphocytes count. Significant differences between the two groups are marked with an asterisk in the legend of the x-axis.*


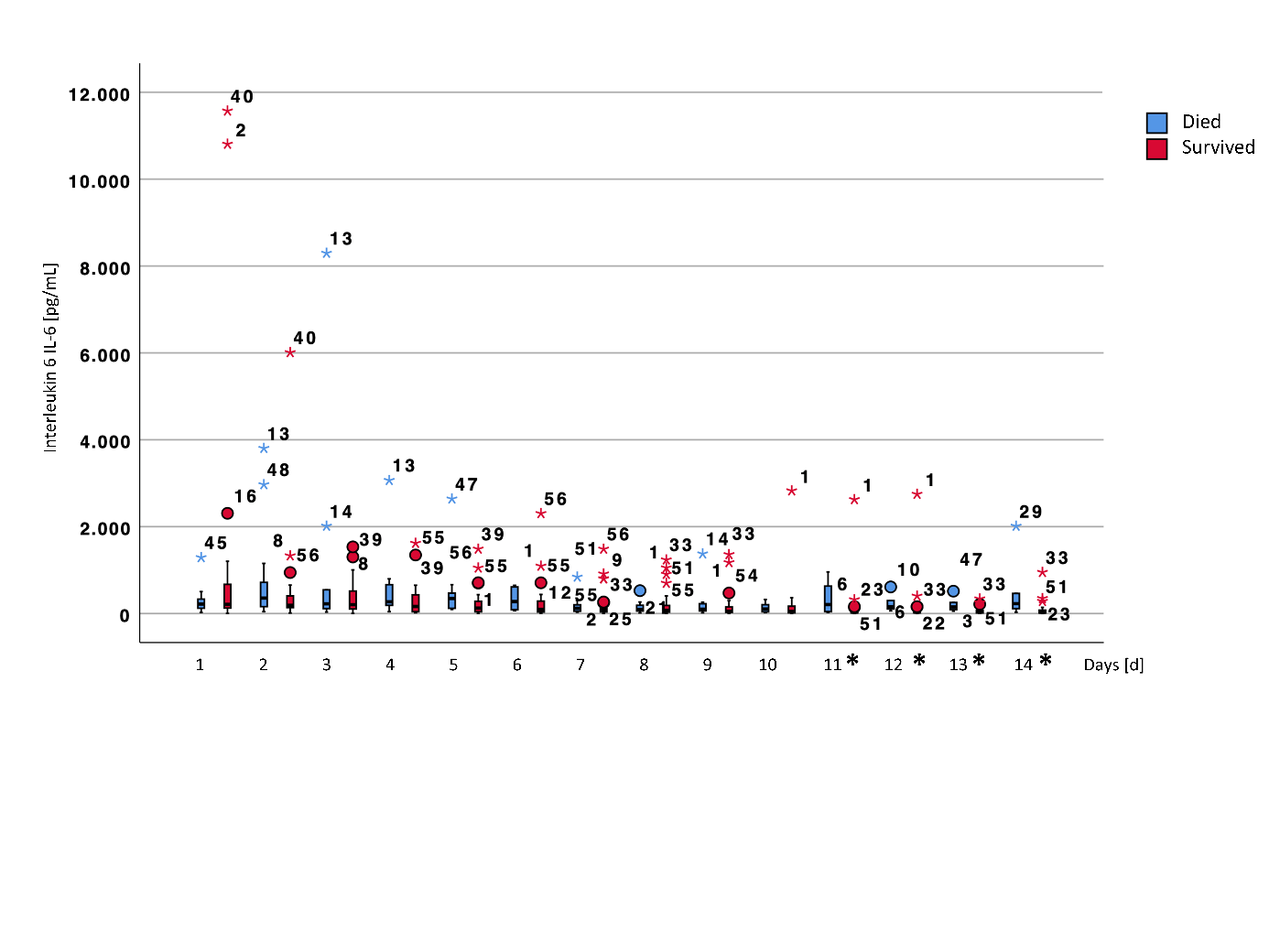


*Daily values for interleukin 6 (IL-6). Significant differences between the two groups are marked with an asterisk in the legend of the x-axis.*


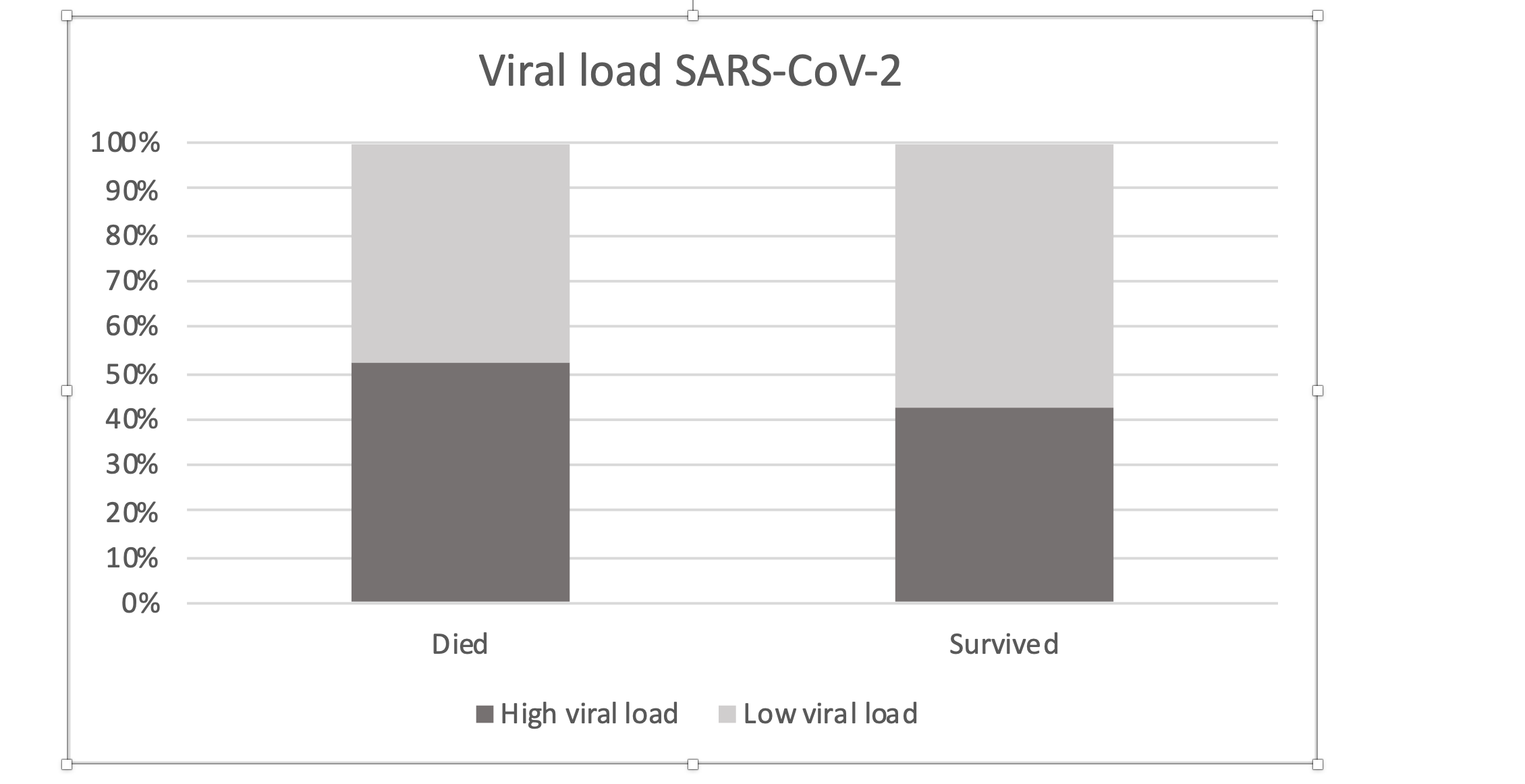


*Frequencies of patients with high viral load (>1*10^6^ copies at least in one probe during the observation period). There was no significant difference between survivors and non-survivors (p=0.579).*


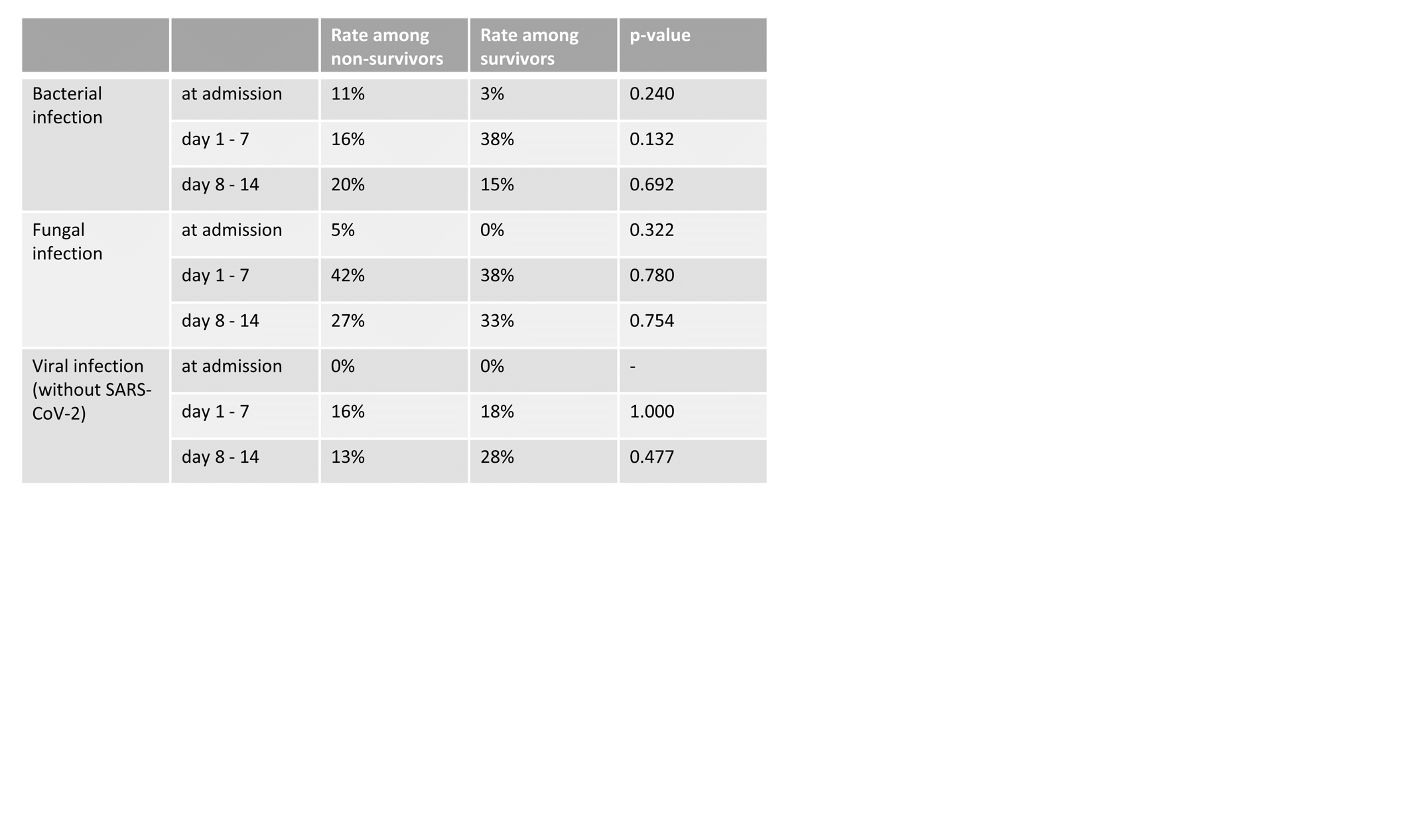


*Frequencies of bacterial, fungal, or additional viral infection.*
