# Supplementary material for: Lower blood pH as a strong prognostic factor for fatal outcomes in critically ill COVID-19 patients at an intensive care unit: A multivariable analysis
Source: PLoS One. 2021 Sep 29;16(9):e0258018. doi: 10.1371/journal.pone.0258018 (PMC8480873; doi:10.1371/journal.pone.0258018)
Supplement: S6 Fig — (DOCX) [file pone.0258018.s014.docx]

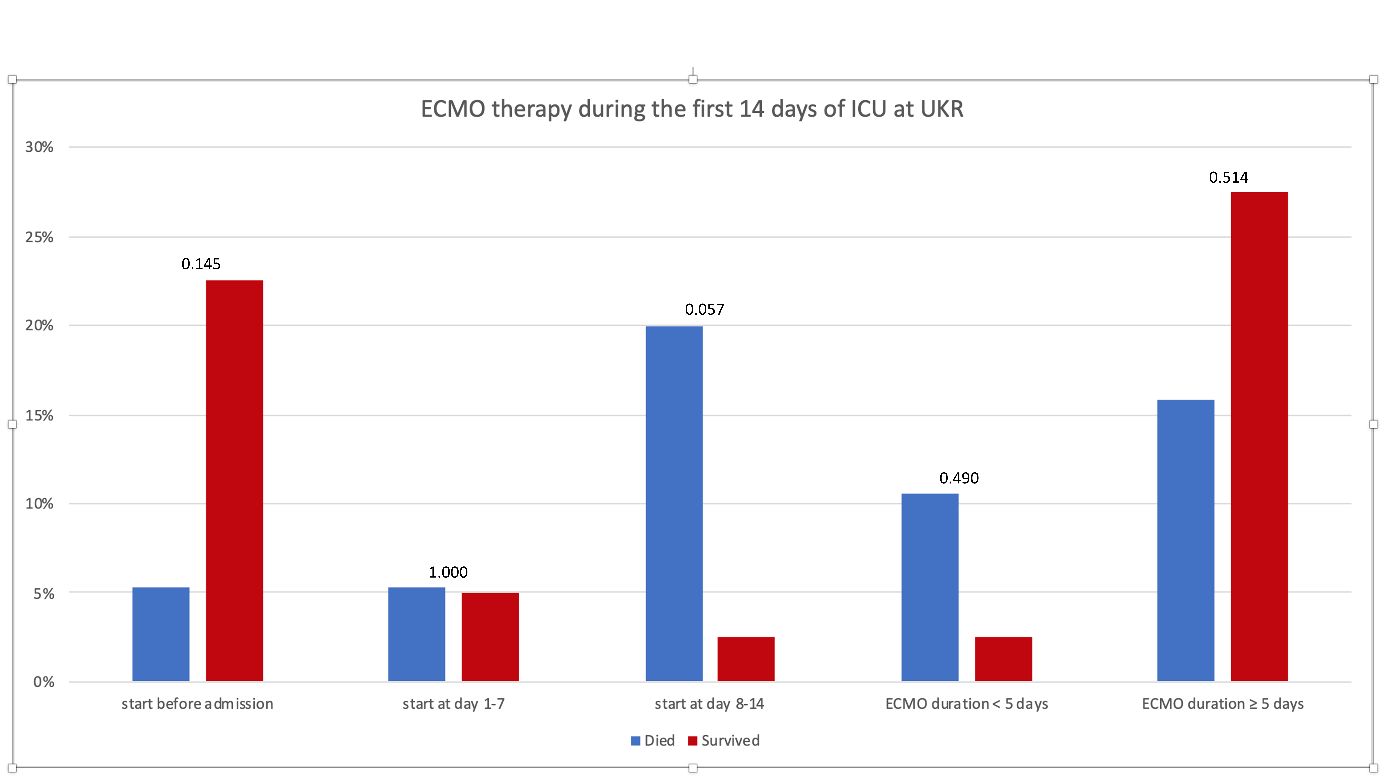


*Classification and frequencies of therapy with extracorporeal membrane oxygenation (ECMO); p-values for comparison between the two groups are stated above the bars.*


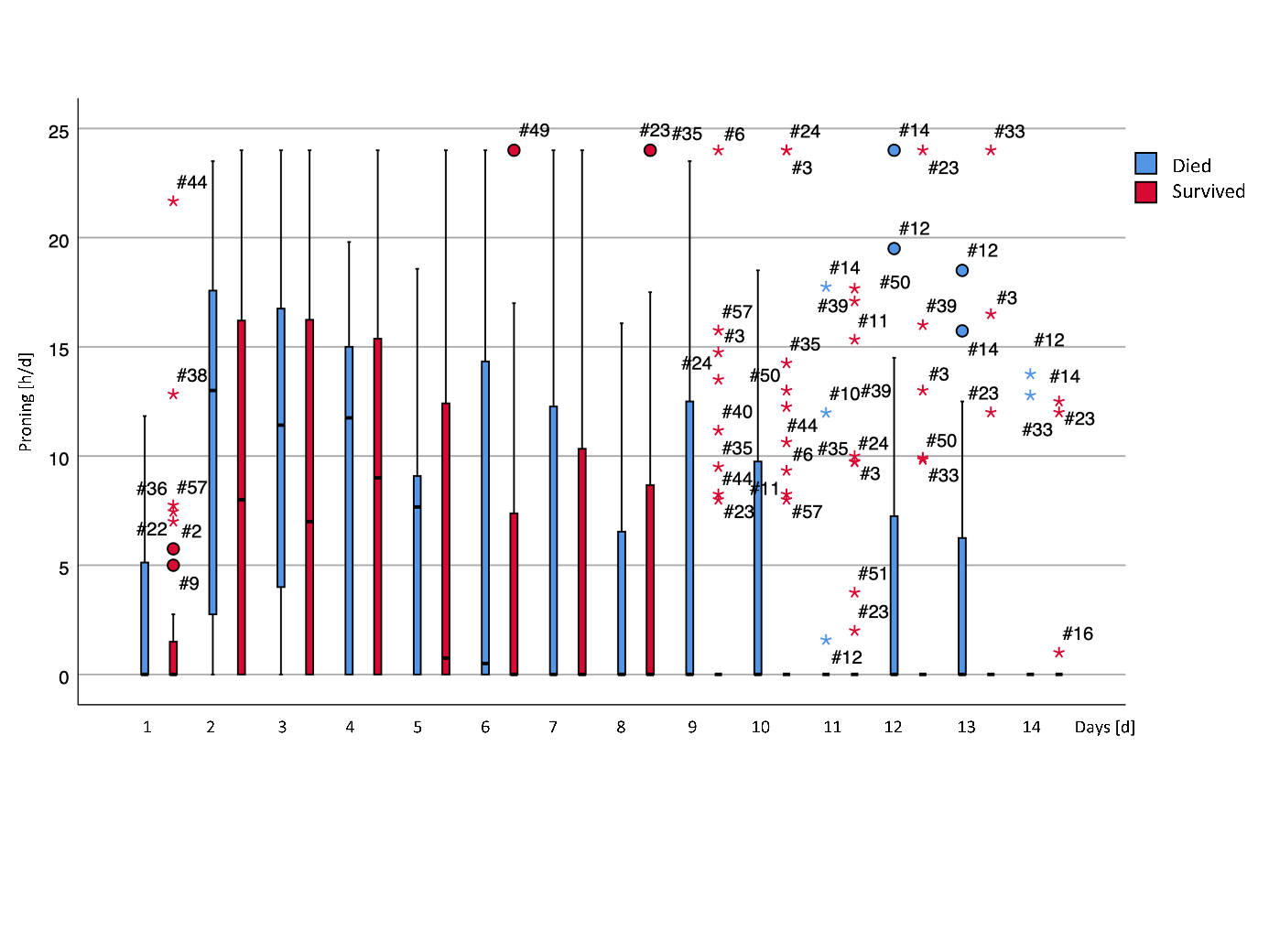


*Daily time of prone positioning (proning). Significant differences between the two groups are marked with an asterisk in the legend of the x-axis.*


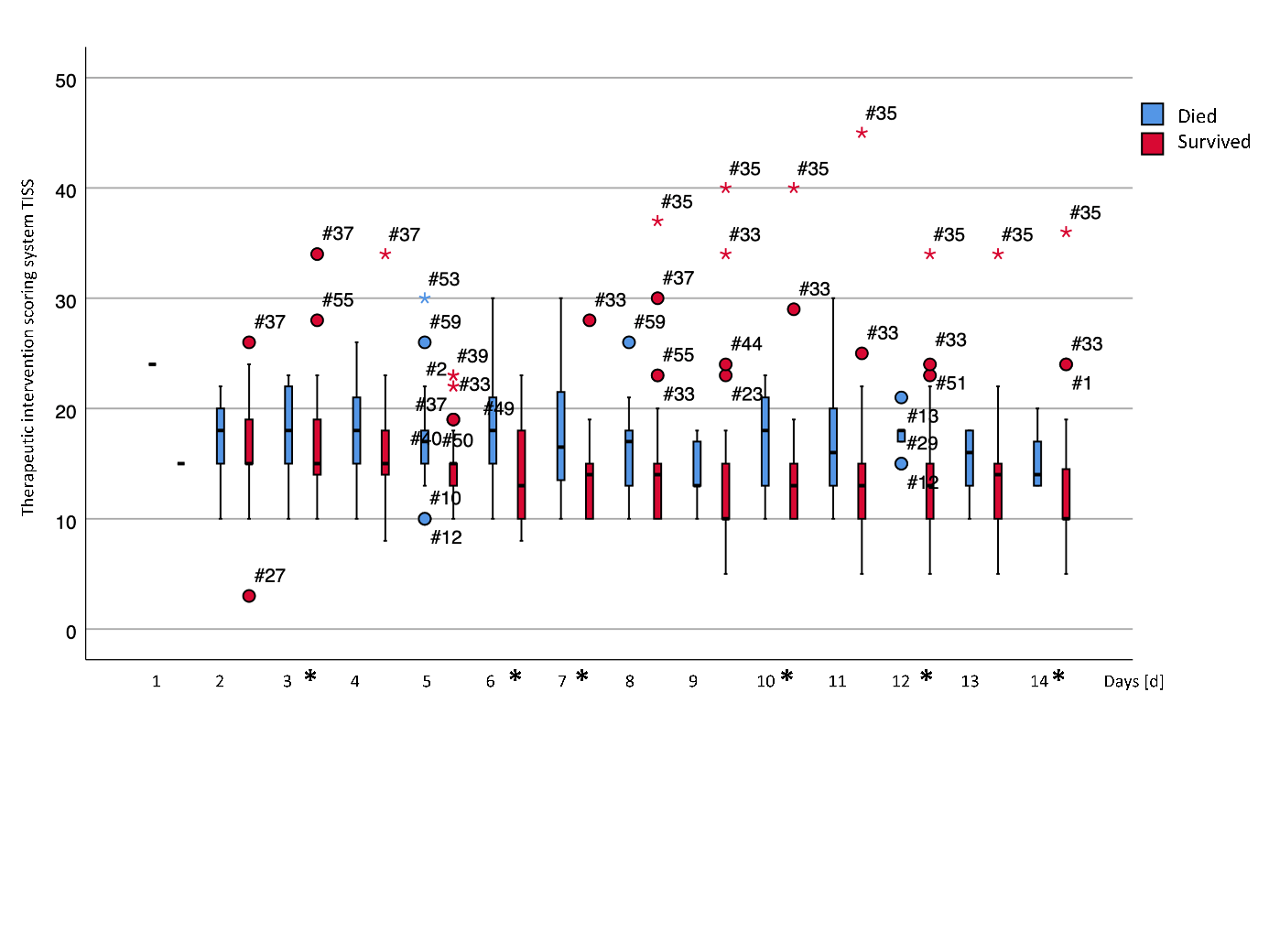


*Daily TISS (therapeutic intervention scoring system) values. Significant differences between the two groups are marked with an asterisk in the legend of the x-axis.*

***
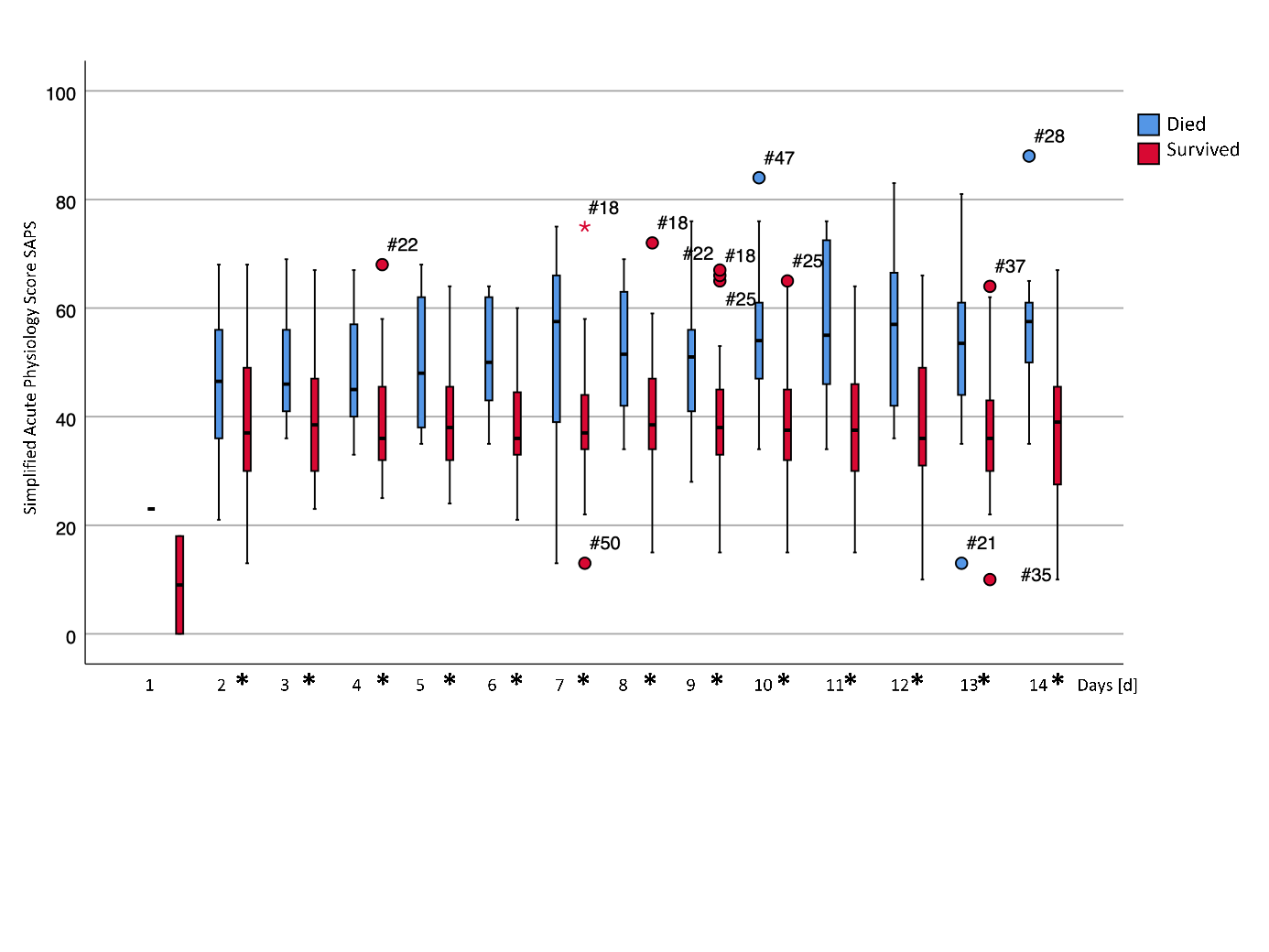
****Daily SAPS (simplified acute physiology score) values. Significant differences between the two groups are marked with an asterisk in the legend of the x-axis.*
