# Supplementary material for: Lower blood pH as a strong prognostic factor for fatal outcomes in critically ill COVID-19 patients at an intensive care unit: A multivariable analysis
Source: PLoS One. 2021 Sep 29;16(9):e0258018. doi: 10.1371/journal.pone.0258018 (PMC8480873; doi:10.1371/journal.pone.0258018)
Supplement: S7 Fig — (DOCX) [file pone.0258018.s015.docx]

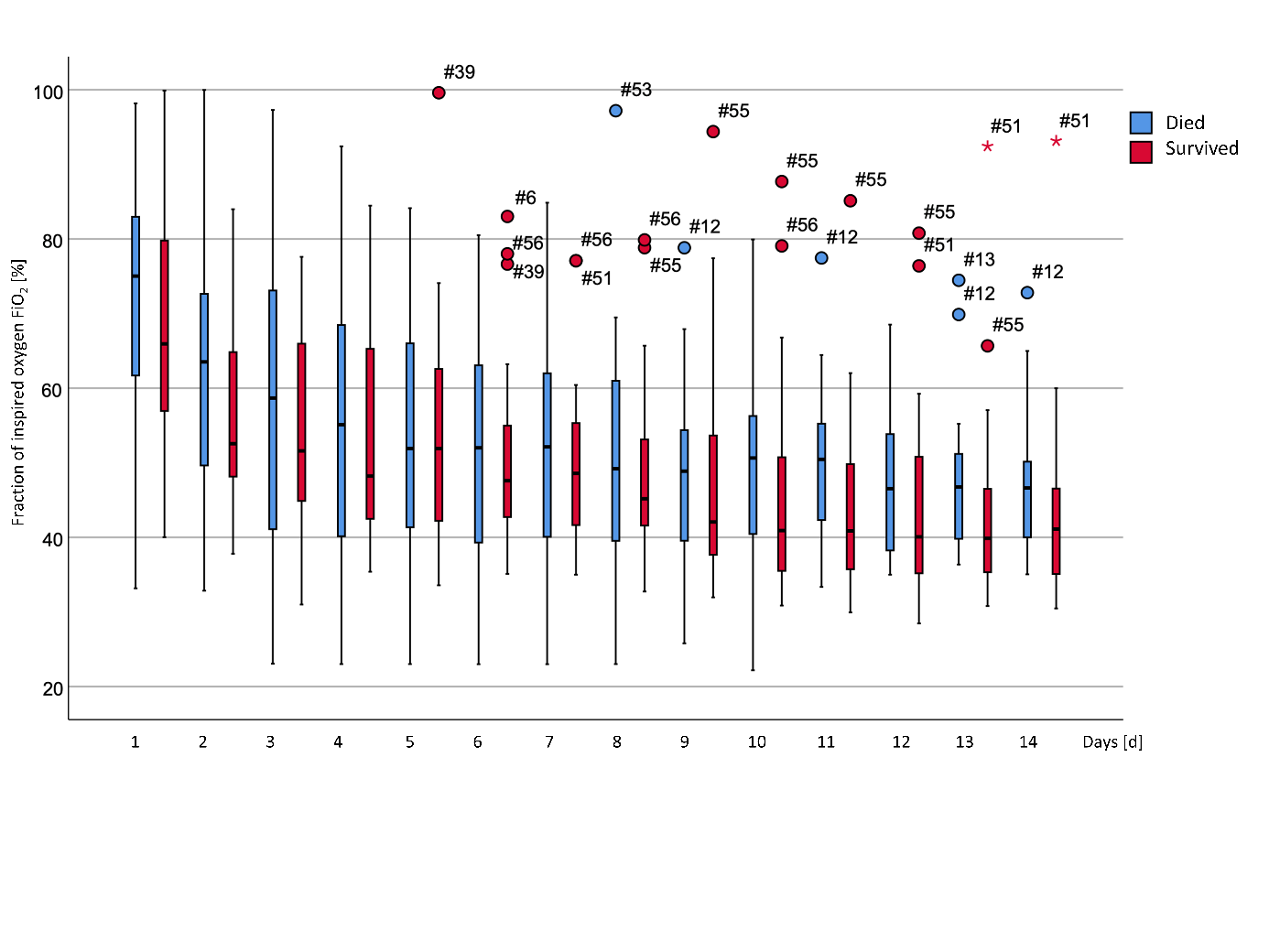


*Daily mean values for the fraction of inspired oxygen (FiO_2_). Significant differences between the two groups are marked with an asterisk in the legend of the x-axis.*


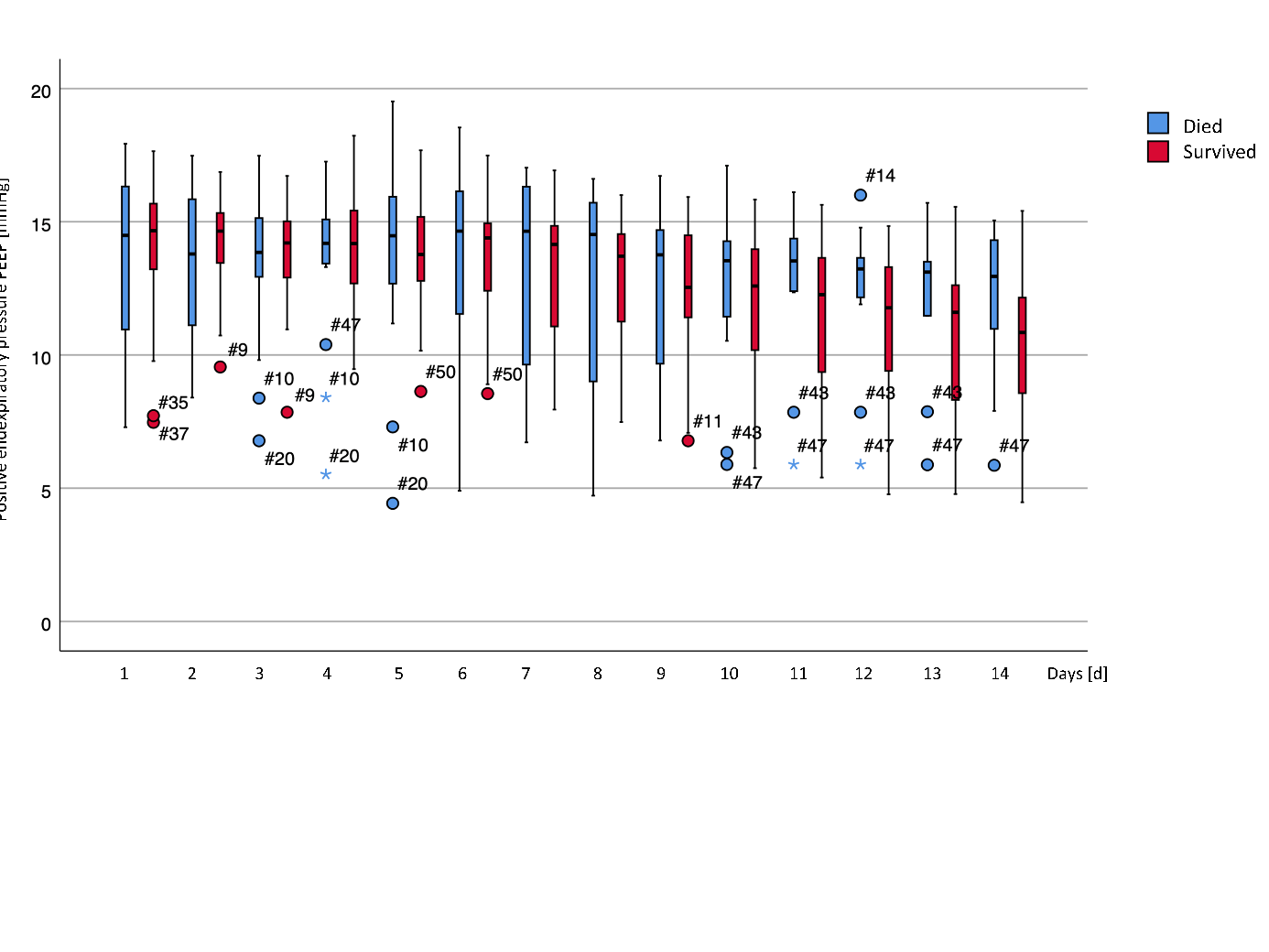


*Daily mean values for positive endexpiratory pressure (PEEP). Significant differences between the two groups are marked with an asterisk in the legend of the x-axis.*


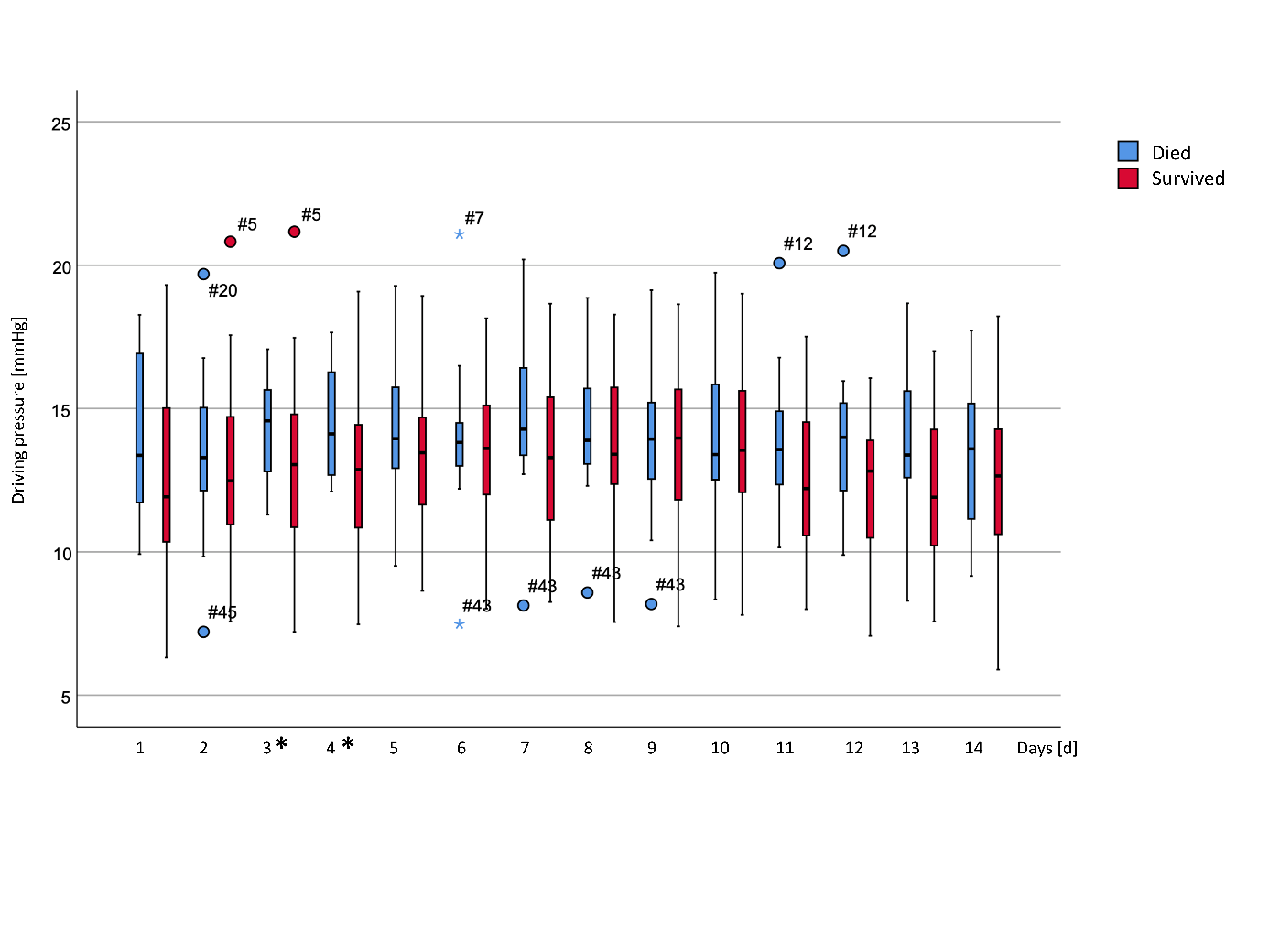


*Daily mean values for driving pressure. Significant differences between the two groups are marked with an asterisk in the legend of the x-axis.*


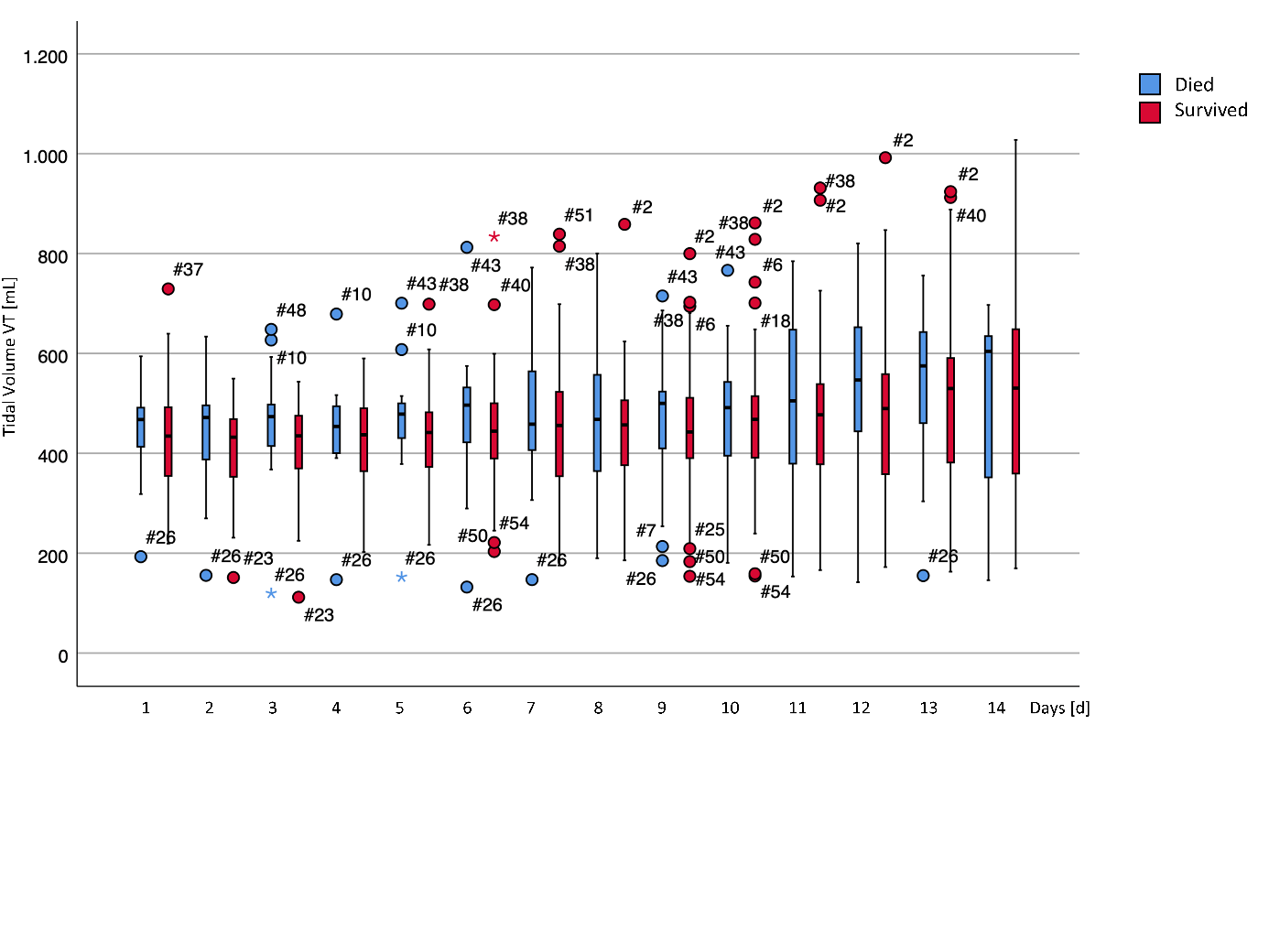


*Daily mean values for tidal volume (VT). Significant differences between the two groups are marked with an asterisk in the legend of the x-axis.*


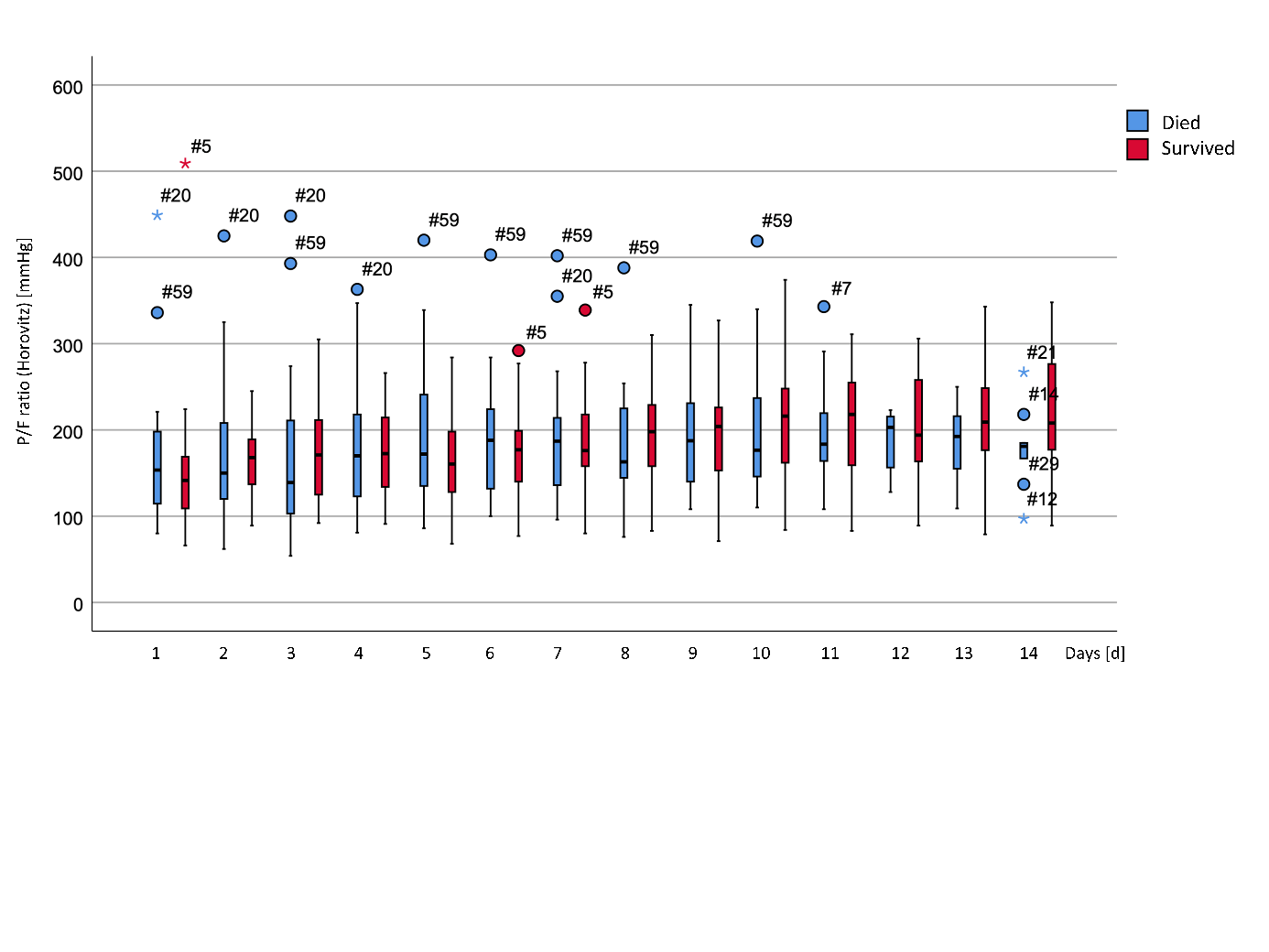


*Daily mean values for the paO_2_/FiO_2_ ratio (P/F ratio). Significant differences between the two groups are marked with an asterisk in the legend of the x-axis.*
