# Supplementary material for: Lower blood pH as a strong prognostic factor for fatal outcomes in critically ill COVID-19 patients at an intensive care unit: A multivariable analysis
Source: PLoS One. 2021 Sep 29;16(9):e0258018. doi: 10.1371/journal.pone.0258018 (PMC8480873; doi:10.1371/journal.pone.0258018)
Supplement: S8 Fig — (DOCX) [file pone.0258018.s016.docx]

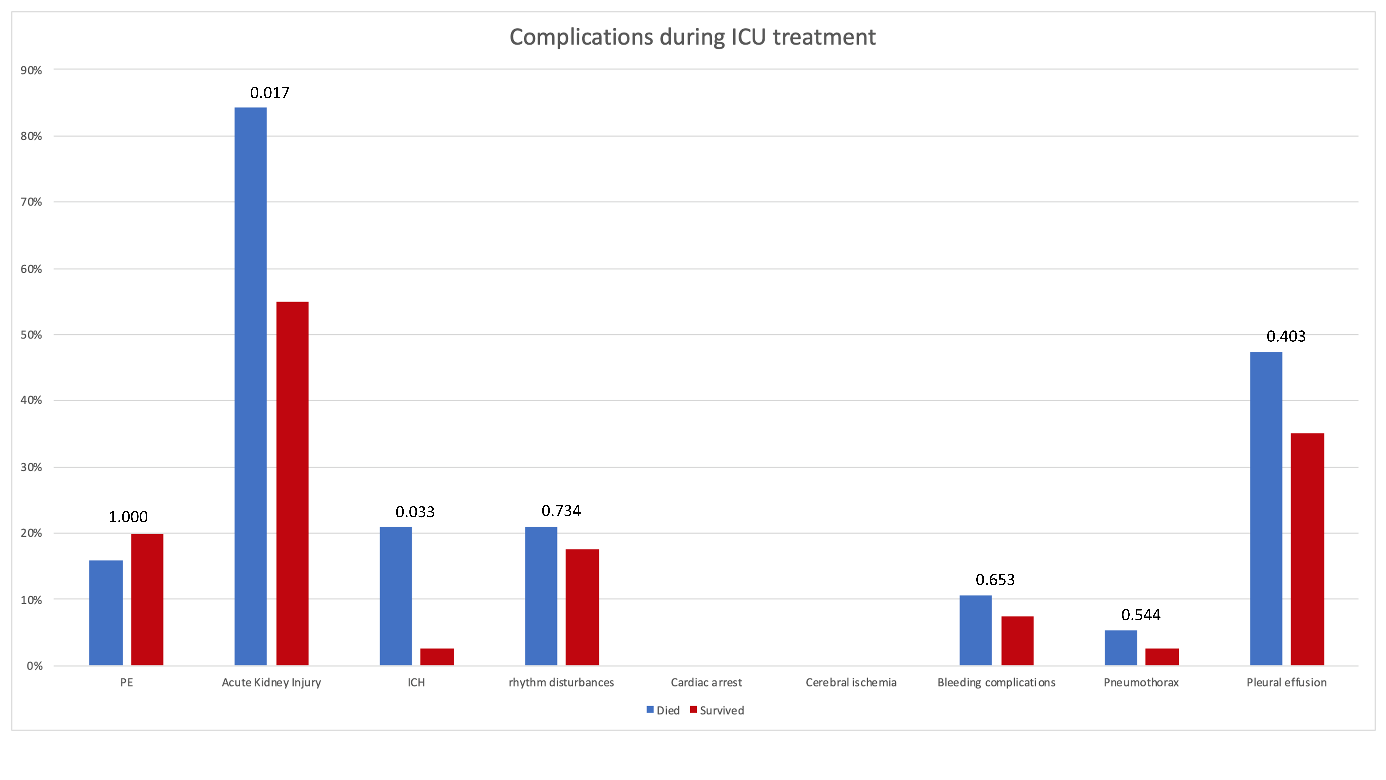


*Classification and frequencies of complications that occurred during the observation period. Pulmonary embolism, PE; intracerebral hemorrhage, ICH; p-values for comparison between the two groups are stated above the bars.*


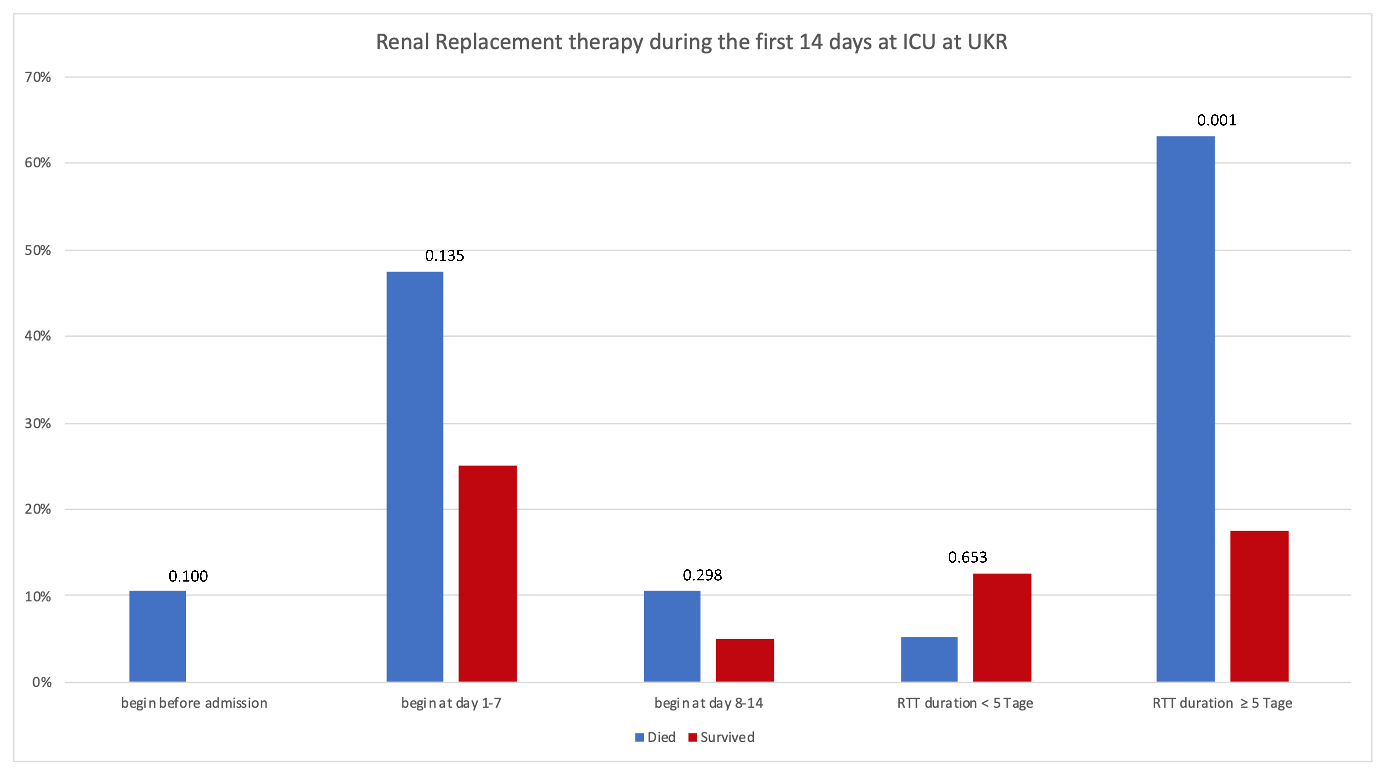


*Classification and frequencies of renal replacement therapy (RRT); p-values for comparison between the two groups are stated above the bars.*
